# Environmental and Health Impacts of Graphene and Other Two-Dimensional Materials: A Graphene Flagship Perspective

**DOI:** 10.1021/acsnano.3c09699

**Published:** 2024-02-13

**Authors:** Hazel Lin, Tina Buerki-Thurnherr, Jasreen Kaur, Peter Wick, Marco Pelin, Aurelia Tubaro, Fabio Candotto Carniel, Mauro Tretiach, Emmanuel Flahaut, Daniel Iglesias, Ester Vázquez, Giada Cellot, Laura Ballerini, Valentina Castagnola, Fabio Benfenati, Andrea Armirotti, Antoine Sallustrau, Frédéric Taran, Mathilde Keck, Cyrill Bussy, Sandra Vranic, Kostas Kostarelos, Mona Connolly, José Maria Navas, Florence Mouchet, Laury Gauthier, James Baker, Blanca Suarez-Merino, Tomi Kanerva, Maurizio Prato, Bengt Fadeel, Alberto Bianco

**Affiliations:** † CNRS, UPR3572, Immunology, Immunopathology and Therapeutic Chemistry, ISIS, University of Strasbourg, 67000 Strasbourg, France; ‡ Empa, Swiss Federal Laboratories for Materials Science and Technology, Laboratory for Particles-Biology Interactions, 9014 St. Gallen, Switzerland; § Nanosafety & Nanomedicine Laboratory, Institute of Environmental Medicine, Karolinska Institutet, 177 77 Stockholm, Sweden; ∥ Department of Life Sciences, University of Trieste, 34127 Trieste, Italy; ⊥ CIRIMAT, Université de Toulouse, CNRS, INPT, UPS, 31062 Toulouse CEDEX 9, France; # Facultad de Ciencias y Tecnologías Químicas, Universidad de Castilla-La Mancha (UCLM), 13071 Ciudad Real, Spain; ⊗ Instituto Regional de Investigación Científica Aplicada (IRICA), Universidad de Castilla-La Mancha (UCLM), 13071 Ciudad Real, Spain; ¶ International School for Advanced Studies (SISSA), 34136 Trieste, Italy; $ Center for Synaptic Neuroscience and Technology, Istituto Italiano di Tecnologia, 16132 Genova, Italy; ¥ IRCCS Ospedale Policlinico San Martino, 16132 Genova, Italy; Δ Analytical Chemistry Facility, Istituto Italiano di Tecnologia, 16163 Genoa, Italy; ▲ Département Médicaments et Technologies pour la Santé (DMTS), Université Paris-Saclay, CEA, INRAE, SIMoS, Gif-sur-Yvette 91191, France; ▽ Nanomedicine Lab, Faculty of Biology, Medicine and Health, University of Manchester, Manchester Academic Health Science Centre, National Graphene Institute, Manchester M13 9PT, United Kingdom; ▼ Instituto Nacional de Investigación y Tecnología Agraria y Alimentaria (INIA), CSIC, Carretera de la Coruña Km 7,5, E-28040 Madrid, Spain; ○ Laboratoire Ecologie Fonctionnelle et Environnement, Université de Toulouse, CNRS, INPT, UPS, 31000 Toulouse, France; ● TEMAS Solutions GmbH, 5212 Hausen, Switzerland; ◇ Finnish Institute of Occupational Health, 00250 Helsinki, Finland; ◆ Center for Cooperative Research in Biomaterials (CIC biomaGUNE), Basque Research and Technology Alliance (BRTA), 20014 Donostia-San Sebastián, Spain; □ Ikerbasque, Basque Foundation for Science, 48013 Bilbao, Spain; ■ Department of Chemical and Pharmaceutical Sciences, University of Trieste, 34127 Trieste, Italy

**Keywords:** 2D nanomaterials, carbon materials, exposure, environment, toxicity, hazard, safe-by-design, biodegradability, test guidelines

## Abstract

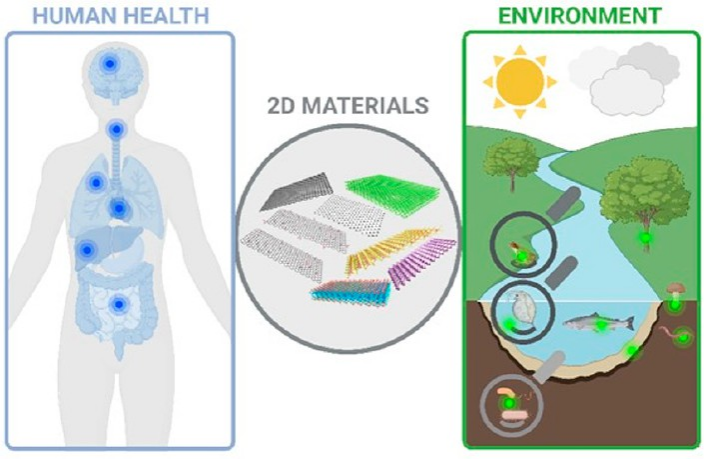

Two-dimensional (2D) materials have attracted tremendous interest ever since the isolation of atomically thin sheets of graphene in 2004 due to the specific and versatile properties of these materials. However, the increasing production and use of 2D materials necessitate a thorough evaluation of the potential impact on human health and the environment. Furthermore, harmonized test protocols are needed with which to assess the safety of 2D materials. The Graphene Flagship project (2013–2023), funded by the European Commission, addressed the identification of the possible hazard of graphene-based materials as well as emerging 2D materials including transition metal dichalcogenides, hexagonal boron nitride, and others. Additionally, so-called green chemistry approaches were explored to achieve the goal of a safe and sustainable production and use of this fascinating family of nanomaterials. The present review provides a compact survey of the findings and the lessons learned in the Graphene Flagship.

## Introduction

Two-dimensional (2D) materials have grown in importance ever since they were discovered to have properties different from their bulk form.^1^ The world of 2D materials spans from the well-known graphene-based materials (GBMs) to the up-and-coming 2D transition metal dichalcogenides (TMDs), 2D transition metal carbides and nitrides (MXenes), and 2D monoelemental materials (Xenes), as well as 2D clays (i.e., layered double hydroxides and layered silicates), metals and alloys. In a very recent assessment on the health and environmental impact of 2D materials, commissioned by the European Chemicals Agency (ECHA),^2^ the features of graphene and other 2D materials and specific toxicity effects of various 2D materials were reported, highlighting some gaps and a need for long-term/chronic studies, particularly for *in vivo* studies using repeated dose administrations. The latter report, which is complementary to the present review, contains an appendix in which the conclusions from each article (more than 650 articles) are summarized in tabular form, along with the physicochemical properties of the tested materials, and the model systems used (i.e., in vitro, in vivo, and/or environmental model systems).^2^

The European Commission’s Future and Emerging Technology (FET) Flagship Project, the Graphene Flagship (www.graphene-flagship.eu), is one of the biggest ever European research initiatives. The rapid development of the field of graphene and emerging 2D materials (i.e., molybdenum disulfide, tungsten disulfide and hexagonal boron nitride, hBN), as investigated in the Flagship (2013–2023), has culminated in the need for a comprehensive review of the findings, especially those related to exposure and hazard. The aim of the present review is thus to provide an update of our previous review published in 2018,^3^ where we focused mainly on graphene family materials. Here, we address GBMs as well as other 2D materials such as TMDs and hBN, both with respect to their (sustainable) synthesis, and their potential impact on the environment and human health, including a detailed survey of the literature published during the past 5 years concerning effects on the main target organs, and the principal environmental compartments. We also address some of the concerns that many researchers face when conducting safety assessments of existing and emerging 2D materials and provide a perspective on future developments in the field.

## Toward “Green” 2D Materials

In order to evaluate the human health and environmental impact of 2D materials and to correlate the effects with their physicochemical properties, it is of paramount importance to perform and provide a thorough characterization. An early description of the electrical characteristics of atomically thin carbon layers (named graphene) prepared by micromechanical exfoliation of highly ordered pyrolytic graphite was published 20 years ago (2004).^4^ Graphene consists of a single layer of monocrystalline graphite with sp^2^-hybridized carbon atoms organized in a honeycomb structure. This specific carbon nanostructure is heavily entering various industrial markets covering numerous applications in the field of materials science and biomedicine. Its properties are surpassing those of other materials making graphene an alternative choice in the development of advanced batteries, fuel cells, reinforced composites, electronic and optoelectronic devices, (bio) sensors, and many others.^5^ Following the seminal discovery of graphene, a myriad of research articles has exploited the advantages of GBMs. In 2014, the Graphene Flagship proposed a framework to eliminate naming inconsistency (e.g., inappropriate use of the term graphene) by classifying all GBMs depending on C/O ratio, lateral dimension, and thickness.^6^ Other 2D materials derived from many different elements were postulated to benefit from similar classification, with *in silico* studies identifying more than 5000 layered bulk compounds, 1825 of which are potentially exfoliable.^7^ In this context, the Graphene Flagship brought onboard the most promising 2D candidates such as transition metal dichalcogenides (TMDs) and hexagonal boron nitride (hBN), and the work package dedicated to the impact on health and environment focused on the assessment of the potential hazards and risks of such materials. In the following sections we highlight the latest advances in 2D materials synthesis that have been evaluated by the Health and Environment work package of the Graphene Flagship. We focus, in particular, on so-called “green” chemistry approaches, a key element of sustainable development of 2D materials, in line with the EU’s Chemical Strategy for Sustainability (2020).

### Graphene-Based Materials

It is challenging to select a synthesis method for GBMs that works in all scenarios, mainly due to the wide variety of potential applications. For example, the same graphene properties will not be required in field-effect transistor to detect coronaviruses (e.g., single/few use(s), low amount, protected from the external environment, reduced exposure to individuals),^8^ or in cementitious composites (e.g., large scale production, long-term use, long exposure to environmental conditions and individuals).^9^ A growing interest in 2D materials for wearable electronics and implantable sensors also requires in-depth consideration,^10,11^ as it is envisaged that these devices may interact with numerous organs and tissues (e.g., skin, brain, mouth, arteries, etc.). Strongly connected to the development and applications of 2D materials, the “safe and sustainable-by-design” (SSbD) concept plays an important role to encourage avoiding the use of hazardous chemicals for their synthesis (e.g., use of gallons of toxic organic solvents or strong acids to prepare graphene or graphene oxide from graphite). This concept goes hand in hand with the overall life cycle assessment (LCA) of GBMs, where the green chemistry principles are fundamental to reduce the environmental impact (and see section Life Cycle Perspective on 2D Materials).^12^

**Figure 1:**
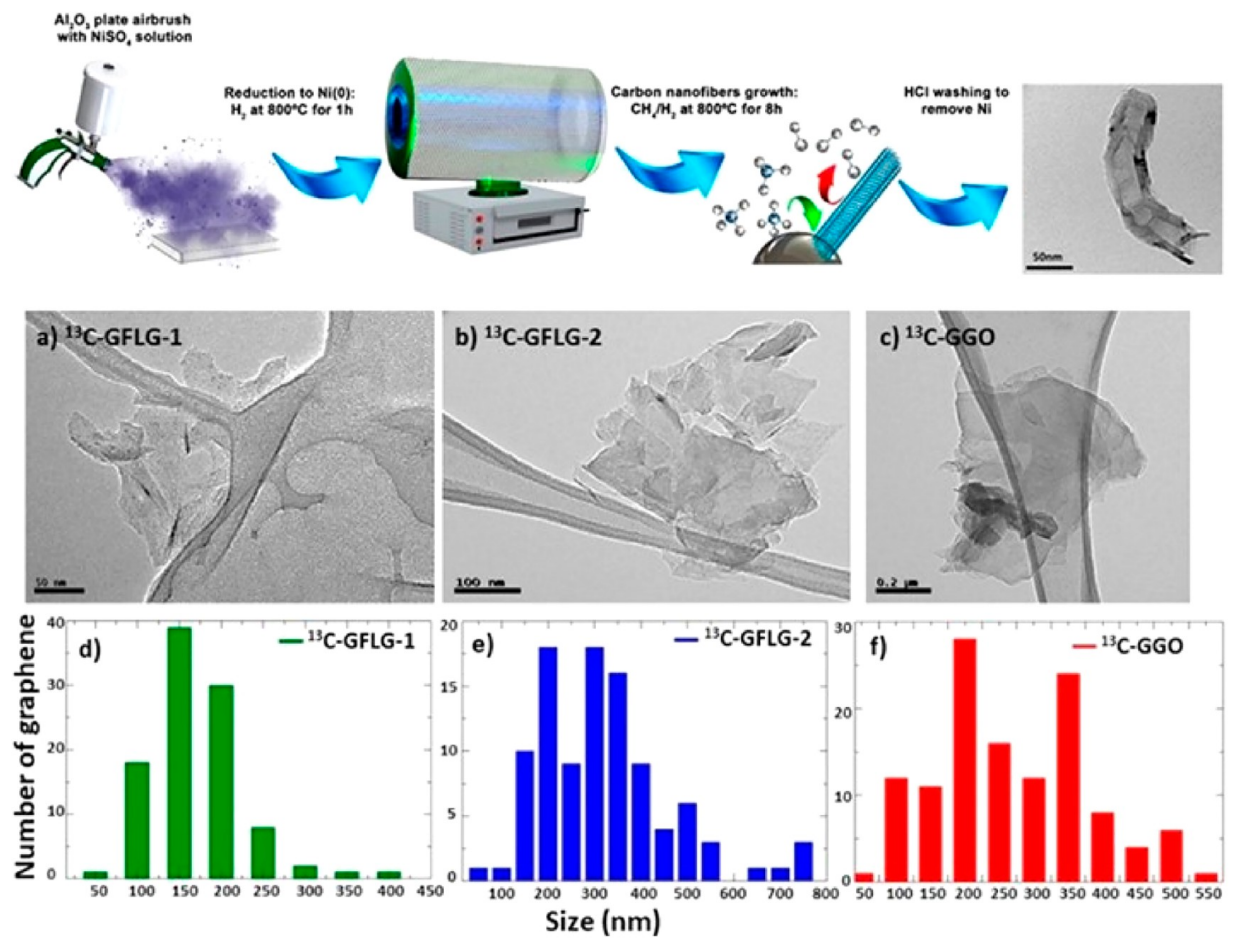
Synthesis of highly enriched ^13^C-graphene materials for biological and safety applications. Top row panels: schematic representation of the synthesis of carbon nanofibers. Top right panel: typical TEM image of the produced fibers. Middle and bottom panels: (a–f) TEM images and size distribution of graphene obtained by exfoliation of the ^13^C graphitized (G) carbon nanofibers: (a,d) ^13^C-GFLG-1, (b,e) ^13^C-GFLG-2, and (c,f) ^13^C-GGO.^13^ Reproduced with permission from ref (13). Copyright 2023, the American Chemical Society.

We have previously described the production methods with the focus on aqueous suspensions of GBMs, since they are usually preferred for *in vitro* and *in vivo* studies.^3^ However, there is a need to improve and develop up-to-date routes for GBM synthesis for even nondispersible forms. For example, we recently developed a synthesis of ^13^C-rich few-layer graphene (FLG) to facilitate the detection and quantification of the material by isotope-ratio mass spectrometry (IRMS) in different biological compartments (Figure 1).^13^

The field is also looking for sustainable production alternatives. In this context, a scalable method for producing large quantities of high-quality graphene flakes using a sugar-based edible wax via three roll millings was proposed.^14^ Co-crystals of graphene and sugar can be also prepared by ball milling of graphite in the presence of carbohydrates, which enables the formation of graphene dispersions in water with lower toxicity in skin cells than pure FLG.^15^ Ball-milling was combined with a viscous glucose syrup to prepare ultrathin (few layers) graphene, hBN, and TMD flake suspensions in water.^16^ These examples highlight the possibility of avoiding the use of organic solvent in the production of 2D materials. It is, however, important to underline that the final materials contain the exfoliating agents necessary to stabilize the suspensions that might not be desirable for a wide range of applications (e.g., in electronics). Many toxicological assessments conducted by the Graphene Flagship partners have also included additional controls to ascertain the impact of the additives present in the dispersions.

In fact, substantial research efforts are devoted to the sustainable production of graphene in powder form by chemical vapor deposition,^17^ which was initially conceived to produce graphene deposited onto a substrate. These approaches rely on the deposition of graphene on easily removable support such as soluble crystals (e.g., cubic NaCl).^18^ This methodology has also been exploited to prepare other 2D materials.^19^ The materials mentioned above will be addressed in the subsequent sections devoted to environmental and human health risks.

Graphene oxide (GO) is the single-layer oxidized form of graphene, where the carbon lattice of graphene is doped with oxygen-containing groups such as hydroxyls, epoxides, and carboxylates. GO is among the most studied GBMs due to its high dispersibility, particularly in water, and its easy access in large quantities obtained from graphite. The most widely used method for GO synthesis is based on the protocol described by Hummers in 1958,^20^ which has been significantly improved in the last 15 years^21,22^ However, this method uses harsh conditions (e.g., high temperature, strong acids, and oxidants). Toward a “greener” and more sustainable production of GO, in the last years, electrochemical conditions have been investigated to achieve GO with tunable properties (e.g., different oxidation levels) in an aqueous environment.^23^ We will not focus on the different methods for the synthesis of GO here as they were described in our previous review,^3^ and they have been recently reviewed by others.^24,25^ Interestingly, we demonstrated that the biodegradability of GO depends on the molecules grafted to its surface.^26^ This knowledge allowed us to prepare for example a biodegradable GO-based conjugate for targeted cancer therapy.^27^ The “degradation-by-design” concept developed in these studies is instrumental for future application of GO and other GBMs in different domains, as the biodegradability of such material is a key aspect to consider and implement in LCA.^28^

When the oxygenated groups on GO are partially removed, one can achieve so-called reduced GO (rGO), endowed with properties similar to graphene. The reduction of GO is obtained by many different physical or chemical methods, either in laboratory conditions or directly in the environment.^29,30^ Many of these methods are “green” as they exploit light irradiation, high temperatures, or natural reductants. The photothermal reduction of GO by laser irradiation was recently reviewed,^31^ but photoreduction occurs also in the environment under sunlight or using different UV wavelengths.^32,33^ Alternatively, the thermal reduction of GO is one of the most used approaches to prepare rGO because it is relatively easy and leads to high-purity material as no chemical reductants are involved.^34^ Microwave reduction is also another sustainable alternative.^35^ The chemical reduction of GO is mainly performed using classical, often toxic, reduction agents (e.g., hydrazine, sodium borohydride, sodium hydrosulfite). Electrochemical conditions have been investigated to achieve GO with tunable properties (e.g., different oxidation levels) in an aqueous environment.^23^ However, it has been shown that chemical transformations of GO may also occur using molecules from the environment. While reduction of GO by phytoextracts has been reported back in 2012,^36^ this has re-emerged in the recent years.^37^ The level of reduction and the purity of the obtained rGO strongly depend on the reducing effect of the plant extracts and on the experimental conditions (e.g., high temperature), as we mentioned above that temperature affects the reduction of GO. There is still a strong push to develop more hydrophilic graphene derivatives with selective functionalization capabilities. Graphene acid and cyanographene are two emerging GBMs that could be very beneficial for nanotherapeutics.^38^

Many challenges remain regarding the preparation of stable water dispersions of graphene using “green” procedures. One of the most exploited methods is the use of ultrasound-triggered mechanical exfoliation in the presence of surfactants that are able to intercalate between graphite layers. However, for biological applications and toxicological evaluations of graphene, it is compulsory to use nontoxic molecules. A derivative of vitamin B2, namely the sodium salt of riboflavin-5′-phosphate, resulted in a very effective means of exfoliating graphite in water leading to the formation of concentrated aqueous dispersions of FLG stable for months.^39^

### Transition Metal Dichalcogenides

The family of TMDs comprises a set of materials composed of a layer of metal atoms (e.g., Mo, W, or Re) sandwiched between two layers of chalcogenide atoms (e.g., S, Se, or Te). Similar to most 2D materials, TMDs can be obtained by bottom-up or top-down approaches. In general, the exfoliation approaches work similarly for the different members of the family. For example, MoS_2_, MoSe_2_, WSe_2_, and WTeS_2_ can be prepared by several methods including CVD, sonochemical reaction, micromechanical exfoliation, liquid phase exfoliation, etc.^40−42^ The selected method will define final material properties. For instance, liquid exfoliated MoS_2_ (e.g., ultrasonicated in water and/or organic solvents) is a semiconductor, while chemically exfoliated MoS_2_ (e.g., prepared via intercalation of organolithium compounds) is metallic. The inversion of the electronic properties is due to a phase transition from hexagonal (2H, semiconducting) to trigonal (1T, metallic) MoS_2_ crystal lattice. *In silico* calculations suggested that sulfur vacancies help to break the kinetic barrier for the 2H-1T transition, which would not take place on a perfect 2H phase.^43^ Besides, experimental studies consistently showed that this transition is attributed to the thermal activation and charge injection that occurs as a consequence of metal doping.^44^ An early protocol for the synthesis of 1T-MoS_2_ was reported in 1986.^45^ First, organolithium compounds were intercalated within the layered structure. Then, the intercalated MoS_2_ was ultrasonicated in water resulting in aqueous dispersions of 1T-MoS_2_. Most research articles reported in recent years still apply this protocol. Functionalized 1T-MoS_2_ was either used to selectively bind enzymes,^46^ or loaded with drugs to combine chemo- and photothermal therapies.^47^ However, the short-term stability is one of the major disadvantage of 1T-MoS_2_. The aging of 1T-MoS_2_ dispersions evidenced that the material oxidizes from Mo(IV) to Mo(VI) in the form of molybdate ions. Light, alkaline pH, and water dissolved oxygen accelerate the degradation of 1T-MoS_2_.^48^ This work is instrumental as it guides proper storage of 1T-MoS_2_ dispersions. This evanescent characteristic of 1T-MoS_2_ might be problematic for long-term applications in electronic devices, but it can be a desirable property to prevent its accumulation in the organisms and the environment. On the other hand, 2H-MoS_2_ sheets are far more stable over time. The production of high quality and large flakes of single-layer 2H-MoS_2_ generally relies on synthetic routes starting from precursors (e.g., bottom-up approach) or on micromechanical exfoliation of MoS_2_ crystals, which are very expensive, difficult to scale up, and have a very low yield. Therefore, the typical method for generating 2H-MoS_2_ dispersions involves liquid phase exfoliation.^49^ The process starts with the addition of bulk material to a solvent. The mixture is then ultrasonicated to break the structure into smaller flakes, then purified by centrifugation. Producing single-layer flakes using these methods results in significantly lower yields. In most studies, a trade-off is made between flake quality and yield, often at the expense of flake quality.

With the aim of improving exfoliation methods, alternative approaches have been developed. Analogously to graphene, MoS_2_ and WS_2_ were successfully exfoliated using ball milling.^50,51^ This process involves the intercalation of a compound within the layers of the materials, aiming to absorb the energy upon ball collision. These collisions result in normal and shear forces that break and exfoliate the bulk materials. Exploiting this methodology, glycine was used as exfoliating agent to prepare aqueous dispersions of MoS_2_ and WS_2_ endowed with long stability.^50^ Notably, the obtained nanosheets can be freeze-dried and stored as powders, which are easily redispersed upon a brief ultrasonication. These ball milled MoS_2_ nanosheets were tested on primary human basophils showing low inflammatory responses.^52^ The results were analogous for MoS_2_ nanosheets produced by the wet-jet milling technique.^53^ The latter work exploits the shear forces produced when a material dispersion passes through a nozzle of adjustable size. This procedure significantly reduces the production times. The production of 2H-MoS_2_ nanosheets was performed using less conventional approaches such as microwave irradiation.^54,55^ Exfoliation of TMDs can be also achieved by combining more than one approach. Ball milling of bulk MoS_2_ in the presence of bile salts was associated with the ultrasonication-assisted exfoliation in water at 0 °C.^56^ This avoids the use of toxic chemicals and solvents. Alternatively, electrochemical methods are commonly combined with ultrasonication-assisted exfoliation. Moreover, large 2H-MoS_2_ crystals (ca. 50 μm) were prepared by electrochemical exfoliation.^57^

### Hexagonal Boron Nitride

In hBN, three atoms of boron are covalently bonded to three atoms of nitrogen forming a honeycomb lattice similar to graphene; indeed, hBN is sometimes referred to as “white graphene”. hBN has excellent thermal conductivity and stability, it is transparent in the UV and visible regions, it is hard and is considered an insulator since it has a band gap around 5.97 eV,^58^ modifiable by various techniques (e.g., doping or functionalization).^59^ All these properties make single-layer hBN a promising material in (opto) electronics, composites, drug delivery, biosensing, gas separation and storage. Like other 2D materials, large-scale production of high-quality hBN flakes is a major challenge. The bottom-up approaches by chemical^60^ or physical^61^ vapor deposition have been used to obtain films of single- to few-layer hBN with applications in electronics. However, biological applications and toxicological studies usually require aqueous dispersions of hBN. Pyrolysis of compounds containing B and N atoms (e.g., boron oxide and urea) has successfully resulted in nontoxic aqueous dispersions of hBN.^62,63^ Exfoliation of bulk hBN applying methods inspired by the Hummers’ method,^64^ using reactions with long-chain amines,^65^ fluorinated molecules,^66^ among others, have been explored. However, these approaches lead to hBN with low yields and quality. Exfoliated hBN was also obtained chemically in the form of ribbons using BN nanotubes as starting material, although the method requires harsh chemicals (e.g., K metal) to obtain mono- and few-layer hBN nanoribbons dispersible in isopropanol.^67,68^

On one hand, the methods mentioned above are specific for BN since they rely on its chemical reactivity. On the other hand, thanks to the structural similarity with graphene, most exfoliation methods developed to exfoliate graphene work also for hBN.^62^ hBN suspensions were successfully prepared by liquid exfoliation assisted by ultrasonication,^49^ microfluidization,^69^ ball-milling,^50^ electrochemical production,^70^ and wet-jet milling.^53^ All these methods can be optimized to achieve 2D hBN suspensions in aqueous media for biological and toxicological studies.^71^

This overview of “green” synthesis approaches provides a basis to better understand the health and environmental impacts of 2D materials, discussed in the following sections.

## Environmental Impact of 2D Materials

**Figure 2:**
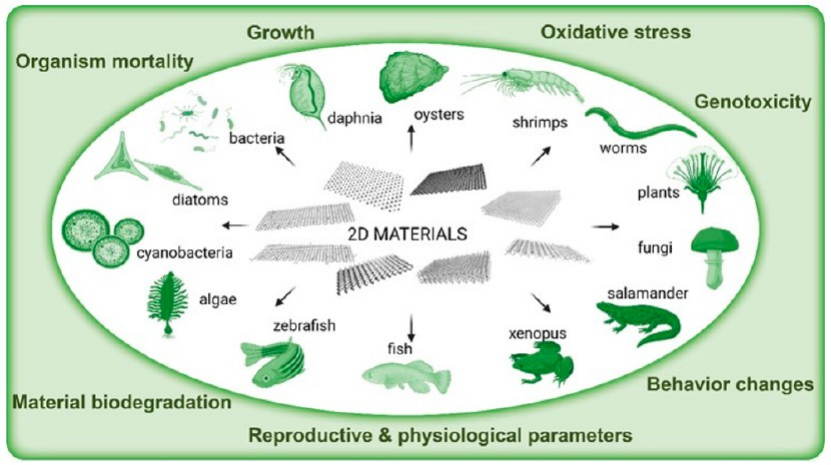
Illustration of 2D materials that can potentially come in contact with living organisms within the diverse range of ecosystems and their possible effects.

Environmental impact assessment is a process starting from scoping to monitoring followed by analysis and reporting. It includes both human health risk assessment and ecological risk assessment (ERA). In this section we will focus on the latter, referring to the evaluation of potential risks arising from 2D materials released by human activities into the environment, with emphasis on studies published during the past 5 years. Figure 2 displays the different types of 2D materials (i.e., GO, rGO, FLG, graphene, MoS_2_, hBN, MXene, and black phosphorus) and the invertebrate and vertebrate aquatic and organisms, fungi, and plants that they can encounter in the different ecosystems, described in this section, together with their possible effects. The assessment aims to identify the impact of these substances on all living organisms within the diverse range of ecosystems. One way to address the challenge of obtaining toxicity data for all organisms in an ecosystem is to select representative species from major taxonomic groups and use them as substitutes for the entire system. The ERA process requires not only a good understanding of the exposome, but also the use of biological tools to assess the hazards posed to organisms by chemical pollution or malfunctions that disturb normal functioning of natural ecosystems. Fish have traditionally been used as indicators to evaluate water quality in aquatic environments, but other groups of organisms such as invertebrates, worms, molluscs, and insect larvae have been shown to be equally or more relevant than fish due to their crucial role in ecosystems. Among vertebrates, amphibians are of particular interest for ecological and physiological reasons, as discussed below.

## 2D Material Impact on Invertebrates

The invertebrates are important organisms in ecotoxicological studies. They are valued for their relatively short life cycle, rapid reproduction, high reproductive rates, and high sensitivity toward pollutants. Moreover, their central position in the food chain, and the fact that they are intermediate consumers (feeding on primary producers such as algae and bacteria and thereafter consumed by larger organisms such as fish and amphibian) puts them in a key position.

**Figure 3:**
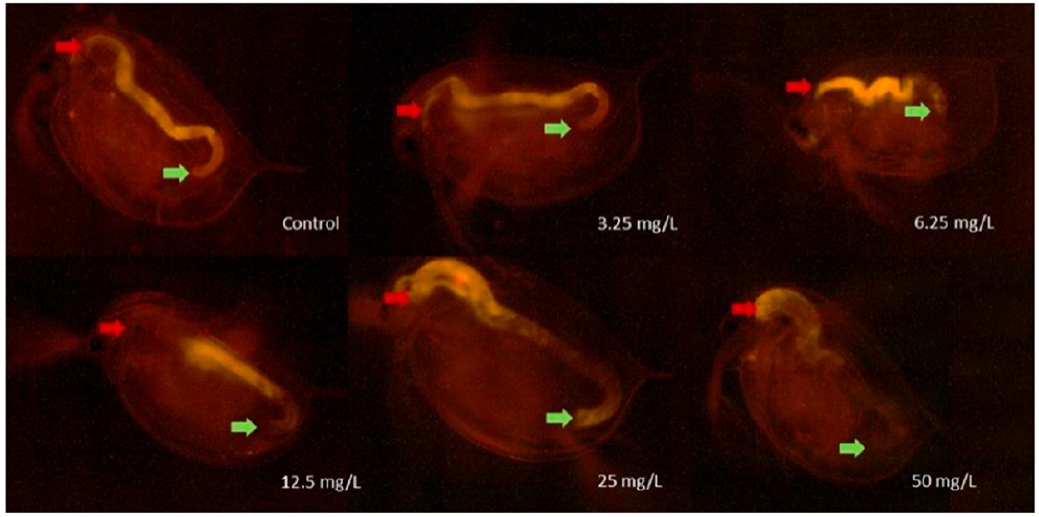
Images of *D. magna* individuals after 48 h of exposure to different concentrations of GO and after feeding on fluorescent microbeads visualized by fluorescence microscopy. The beginning of the digestive tract is marked by red arrows, while the end is marked by a green arrow.^76^ Reproduced in part with permission under a Creative Commons CC BY 4.0 License from Fekete-Kertesz, I.; Laszlo, K.; Terebesi, C.; Gyarmati, B. S.; Farah, S.; Marton, R.; Molnar, M. Ecotoxicity Assessment of Graphene Oxide by *Daphnia magna* through a Multimarker Approach from the Molecular to the Physiological Level including Behavioral Changes. *Nanomaterials (Basel)* 2020, *10*, 2048. Copyright 2020, MDPI, Basel.

One of the most studied invertebrates to evaluate ecotoxicity is the crustacean *Daphnia*, which is widely distributed in freshwater ecosystems and is easy to culture, making it convenient to use in controlled environments. The daphnia species *Daphnia magna* and *D*. *pulex* as well as *Ceriodaphnia dubia*, are largely used in standardized toxicity tests regulated by the Organisation for Economic Co-operation and Development (OECD) and International Standardization Organization (ISO). *Daphnia spp*. is used in test guidelines OECD TG 202 and ISO 6341 to determine acute toxicity following 48 h exposure to young daphnids (aged <24 h). The test end point is usually immobilization (loss of ability to move within 15 s under soft agitation) or mortality.^72−74^ Calculated effective concentration EC_50_ values at 48 h refer to concentration levels that result in immobilization (or mortality) of 50% of daphnids at the end of the exposure period. A recent review^75^ reported acute effects after short-term exposure (48 and 72 h) of GBMs in daphnia under standardized OECD TG 202, ISO 6341 and under modified tests. Chronic assays focused on reproductive capacity of *D. magna*(72) and *C. dubia*(73) and involves exposure to various concentrations of the test substance over a 7- or 21-day period. The test is performed in a static but renewable water system. Mortality rate of parents, time to produce initial brood, number of live offspring produced by exposed organisms (parents) are compared to those of control organisms to determine potential impact on reproduction. Less conventional end points are also considered, including physiological and oxidative stress parameters and sublethal end points such as heartbeat rate, feeding activity, reactive oxygen species (ROS)^73,76^ accumulation, oxidative stress and enzyme activities.^74^ Recent daphnid studies have included functionalized GO (0 to 50 mg/L, and some >140 mg/L) as extensively described in recent reviews^75^ with immobilization and mortality seen after 48 or 72 h of GO. GO from various suppliers exhibited EC_50_ 48 h values that ranged from 21 mg/L using GO (0.5–3.0 μm)^77^ to 44.3 mg/L with GO (200–300 nm).^74^ Similar values of EC_20_ of 50 mg/L GO were established for mortality, while for physiological and behavioral end points, values ranged from 8.1 mg/L (feeding activity) to 14.8 mg/L (immobilization) (Figure 3).^76^ Interestingly, functionalization of GO with carboxyl, imidazole, or poly(ethylene) glycol (PEG) reduced acute toxicity.^78^ The authors used a GO modified by linking chloroacetic acid to the hydroxyl groups, imidazole to the carboxylic groups, or diaminotriethylene glycol to the epoxides.^78^ These moieties changed the chemical structure of GO and likely reduced the cytotoxic effects of the abundant oxygenated groups as GO contains a basal level of stabilized radicals responsible for triggering cellular toxicity; indeed, it is notable that GO may cause the depletion of glutathione (GSH), the main antioxidant molecule in the cell, thus evoking oxidative stress in cells.^79,80^ Moreover, if further functionalities are able accelerate the catalytic activity of the enzymes involved in the degradation of GO, then the degradation could also consequently be enhanced.^26^

Changes in superoxide dismutase (SOD) and lipid peroxidation (LPO) of *Daphnia* suggested elevated GO-mediated oxidative stress and damages.^74^ In a 21-day study, the mortality rate of *Daphnia* parents (F0 generation) increased,^72^ but another similar study reported no effect on daphnid survival with 1 mg/L of GO for F0 and F1 generations, with no effect on F0 reproduction.^78^

Other invertebrates have been used, but to a lesser extent than daphnids. Oysters are also valued due to being filter feeders.^81^ They are particularly sensitive to changes in water quality and pollution levels. Moreover, they are easily cultured in laboratory settings. Among them, *Crassostrea virginica* (Eastern oysters) is valuable for nanotoxicity assessment thanks to their filtering capacities.^82^ As a bivalve, oysters have mechanisms for internalizing both nano- and microscale particles, such as endocytosis and phagocytosis, respectively.^83^ Few-layer GO (FLGO) can affect oyster health.^84,85^ After 72 h,^85^ oysters exposed to 10 mg/L of FLGO showed increased lipid peroxidation, indicating oxidative stress. Oysters exposed to 1 and 10 mg/L showed reduced total protein levels in digestive gland tissues. Epithelial inflammation was observed in gills as loss of mucous cells, hemocytic infiltration, and vacuolation. In a similar 14-day study on *C. virginica*, elevated lipid peroxidation, ROS induction and changes in glutathione-S-transferase (GST) in tissues of gills and digestive gland were reported with 2.5 and 5 mg/L of FLGO. A recent 24 h study on *C. gigas* (pacific oysters) demonstrated the paradoxical effects of various types of GO. GO (e.g., 0.2–8 μm and 30% oxygen content) at 0.1 mg/L was found to worsen copper-mediated embryo-larval toxicity while rGO (with similar dimensions to GO but with 16.8% oxygen content) comparatively mitigated the effects by way of decreasing copper bioavailability.^86^

*GSTThamnocephalus platyurus* is also used as described in the standardized protocol ISO 14380 to determine lethal effects of toxicants after 24 h exposure. *Heterocypris incongruens* can also be used according to ISO 14371 for the determination of lethal and sublethal effects of contaminated sediments after 6 days of exposure.^87^ The results showed that the benthic crustacean *H. incongruens* was more resilient than the planktonic *Thamnocephalus* in the case of GO under different oxidation states from 0.39 mg/L to 25 mg/L as measured from viability tests. This difference in sensitivity was due to contrasting shell composition (robust calcified carapace for *Heterocypris* possesses versus outer shell of poly saccharide chitin for *Thamnocephalus*). Notably, acute toxicity was more pronounced with highly hydrophobic GO, allowing direct interaction with crustacean filtration apparatus and mechanical damage resulting in higher mortality.^87^

The common shrimp *Palaemon pandaliformis* has also been used in the 96 h acute toxicity assay to determine lethal concentrations of GO.^88^ Even if 5 mg/mL GO did not present acute ecotoxicity, interaction of GO with trace elements increased toxicity as seen from decreased lethal concentration LC_50_. It was suggested that coexposure of GO with trace elements impaired routine metabolism of *P. pandaliformis*. *Hydra attenuate*, *Artemia salina*, *Chironomus sancticaroli*, and *Caenorhabditis elegans*, primary or lower-level consumers, which are also used in ecotoxicology studies. Another study showed that GO induced no toxicity after 96 h at concentrations up to 100 mg/L in *H. attenuate* (mortality as end point), *A. salina* (body growth as end point), and *C. elegans* (recovery, fertility, reproduction, and growth).^89^ Similarly, weak toxic effects induced by GO up to 72 h exposure were noticed also in *Artemia franciscana* nauplii and adults. Mortality and activation of the xenobiotic detoxifying and antioxidant enzyme GST were observed only for the latter at the highest dose (100 mg/L).^90^ In addition, *Chironomus larvae* exposed to GO for 7 days did not exhibit any mortality or teratogenic effects despite reduction in final larvae length (from 4.4 to 10.1%), even at low concentrations of EC_50_ 38.74 mg/L.^91^

Regardless of invertebrate model organism, natural organic matter (NOM) can increase the toxicity of graphene-based nanoparticles to organisms by enhancing nanomaterial stability.^72^ Toxicity of GO was, however, lower with NOM, using mortality as the end point, decreasing from 111.4 mg/L to 84.3 mg/L in acute investigations and from 3.3 mg/L to 9.7 mg/L in the chronic one.^72^ Reproductive capacity end point as well as oxidative status followed the same pattern.^72^ Functional attachments such as carboxyl, imidazole, and PEG were also found to alleviate 48 h daphnid toxicity (immobilization) of GO in daphnid survival, growth, and reproduction.^78^

With regard to nongraphene 2D materials, results are similarly variable. Molybdate was found to have no effects on *Daphnia* acetylcholinesterase inhibition *in vitro*, but effects were observed *in vivo* at concentrations under the 48 h LC_50_ value of 2847.5 mg/L, also inhibiting reproduction and growth.^92^ Molybdenum toxicity in aquatic systems is highly dependent on the form of molybdenum salts and is also influenced by background water quality. The toxicity of common molybdate ions was reported as ammonium molybdate being the most toxic, followed by molybdenum trioxide, then hexavalent molybdate.^93^ Sodium tungstate was found to exhibit low toxicity in *Daphnia* and is not considered an aquatic toxicant^94^ although long-term exposure of tungsten carbide resulted in increased time to initial reproduction and, resuspended particles found to impact survival and reproduction.^95^ No data exist for hBN, to our knowledge, but boron alone was found to be the least toxic among 36 metals and metalloids in an ostracod *Cypris subglobosa* aquatic system.^96^ A recent study in Italy showed no ecological hazard effects of boron at concentrations detected in groundwaters, using *Daphnia* as an ecotoxicology readout.^97^ In contrast, a three-year study conducted in a Canadian oil end pit lake predicted that boron posed very high toxicological risk to aquatic organisms.^98^

## 2D Material Impact on Vertebrates

Amphibian models are important in ecotoxicology in the study of emerging contaminants such as nanomaterials. Toads, frogs, newts, and salamanders are of interest because of their potential to investigate the mechanisms of toxicity of pollutants on global health. In particular, due to their ability to easily breed and develop in captivity, measurement sensitivity and reproducibility as well as ease with which to conduct genomic analysis, *Xenopus laevis*, and the salamanders *Pleurodeles waltl* and *Ambystoma mexicanum*, have been used in ecotoxicology studies. Regardless of amphibian organism, intestinal absorption of carbon-based nanomaterials appears to be limited after oral administration, and the materials are quickly excreted. It has been demonstrated that growth inhibition observed in amphibians is due to physical blockage of the gills and/or digestive tract, limiting exchange surfaces between the gills and/or gut lumen and the internal wall, leading to a decrease in absorption of nutrients and/or gas, resulting in anoxia. The role of oxidation degree and surface functions of GO in toxicity was demonstrated in *X. laevis* by subjecting GO to thermal reduction at 200 and 1000 °C to produce rGO with different chemical surface functions.^99−101^ Using the standard ISO 21427-1 exposure of 12 days to 0.1 to 50 mg/L of GO and rGO, GO caused disruptions in the erythrocyte cell cycle, leading to cell accumulation in the G0/G1 phase. Low concentrations of GO (0.1 mg/L) induced genotoxicity in exposed larvae through oxidative stress. However, the reduction of GO eliminated genotoxicity at low concentrations. Some genes involved in oxidative stress response and inflammation were significantly overexpressed. However, some detoxification processes also occurred as supported by the induction of *cyp1a1*. On the contrary, no significant modulation of gene expression was noted with rGO. Surface analysis suggested that epoxide groups may be responsible for genotoxic effects of highly oxidized GO. The result obtained using the *Xenopus* model proposes that thermal reduction of GO could be a safer alternative for developing environmentally friendly materials.

**Figure 4:**
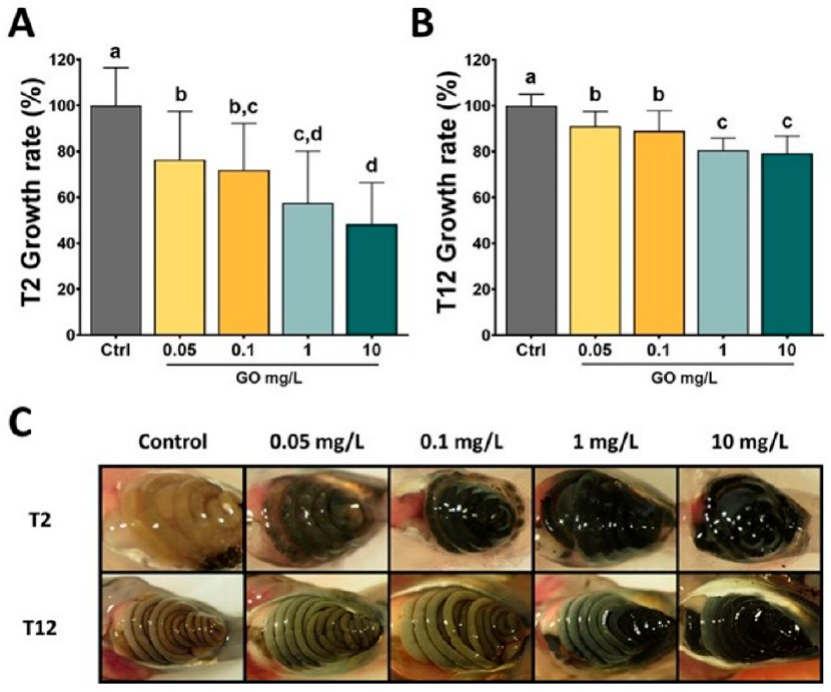
Ecotoxicology of 2D materials: evaluating the effects of GO on *Xenopus laevis* tadpoles. Normalized growth rate determined after 2 days (A) or 12 days (B) of exposure to increasing GO concentrations. (C) Pictures of GO intestinal accumulation in tadpole larvae after 2 days or 12 days of exposure.^101^ Reproduced with permission from Evariste, L.; Mouchet, F.; Pinelli, E.; Flahaut, E.; Gauthier, L.; Barret, M. Gut Microbiota Impairment following Graphene Oxide Exposure is Associated to Physiological Alterations in *Xenopus laevis* Tadpoles. *Sci. Total Environ.* 2022, *857*, 159515. Copyright 2022, Elsevier.

Endocrine disruption of the same GO and rGO using a triiodothyronine (T3)-induced *Xenopus* metamorphosis (adapted) assay was also investigated.^102^ Previously observed effects described in *X. laevis* tadpoles were associated with toxicity rather than to thyroid endocrine disruption. The results indicated that GO and rGO (after 96 h of exposure to increasing concentrations) potentiated the effects of exogenous T3 with a more marked effect of GO compared to rGO. T3 quantifications in the exposure media indicated adsorption of this hormone on GBMs, increasing its bioavailability because GO and rGO accumulated in the gut and the gills. GO and rGO did not disrupt the thyroid pathway in amphibians but that adsorption properties of these nanomaterials may increase the bioavailability and toxicity of other pollutants.^103^ The potential link between gut microbial communities and host physiological alterations induced by GO at low concentrations of up to 10 mg/L was also investigated (Figure 4).^101^

Larvae did not exhibit significant differences in intestinal weight compared to unexposed larvae after 2 days. However, after 12 days with GO at 10 mg/L, significant decrease in intestinal weight was observed, indicating impairment of intestinal development. No developmental stage delay was observed, but GO exposure led to a dose-dependent growth inhibition. Genotoxic effects observed at 0.1 mg/L were associated with gut microbiota remodelling characterized by an increase in the relative abundance of *Bacteroides fragilis*. Growth inhibitory effects was associated with a shift in the *Firmicutes/Bacteroidetes* ratio, while metagenome inference suggested changes in metabolic pathways and upregulation of detoxification processes. These findings implicate gut microbiota as an important biological compartment that should be considered in ecotoxicological studies, as structural or functional impairments could lead to host fitness loss. To date, very few studies focused on other 2D materials such as MoS_2_, WS_2_, or hBN. However, it has been reported that free boron present in tested boron-containing nanomaterials is beneficial for *Xenopus* tadpole metabolism.^104^

## 2D Material Impact on Fish

**Figure 5:**
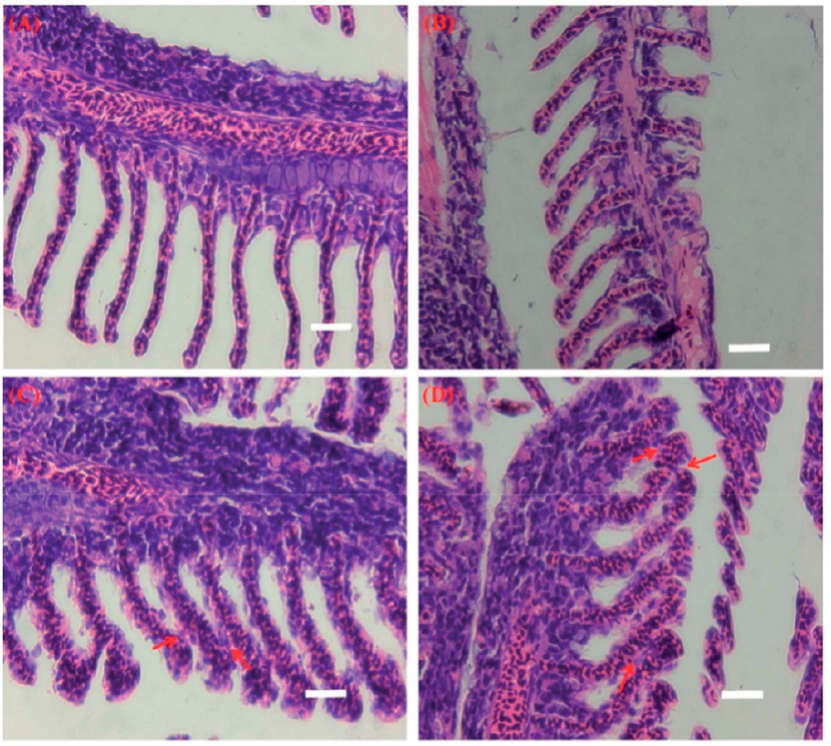
Light micrographs of gill tissue samples from (A) control and chitosan functionalized CS-MoS_2_ at (B) 2 mg/L (C) 10 mg/L and (D) 20 mg/L) Scale bar = 400 μm.^107^ Reproduced with permission from Yu, Y.; Yi, Y.; Li, Y.; Peng, T.; Lao, S.; Zhang, J.; Liang, S.; Xiong, Y.; Shao, S.; Wu, N.; Zhao, Y.; Huang, H. Dispersible MoS_2_ Micro-Sheets Induced a Proinflammatory Response and Apoptosis in the Gills and Liver of Adult Zebrafish. *RSC Adv.* 2018, *8*, 17826–17836. Copyright 2018, the Royal Society of Chemistry.

With a projected increase in production volumes and uses of 2D materials, the risk of exposure of fish to these materials becomes a reality. The most studied material in this respect is GO,^105,106^ while limited studies on fish following exposure to TMDs (e.g., MoS_2_) are available (Figure 5).^107−109^ According to the literature, no dose-dependent acute toxicity of GBMs in adult fish was reported.^106^ However, this must be interpreted with caution as standardized test guidelines for fish acute toxicity assessment (e.g., OECD TG 203) are not always followed.^75^

Taking into consideration only the studies that have measured the actual exposure concentration maintained in the water column throughout the exposure period, the highest concentration of GBMs (in this case GO) tested using fish (zebrafish, *Danio rerio*) was 6.4 mg/L,^111^ which did not result in any fish mortalities, even following 14-day exposures. Due to instability of the tested material, testing of higher concentrations will likely require addition of appropriate protocols (e.g., agitation, use of dispersants, or renewals), which are currently under evaluation for their use in the applicability of OECD TG 203 for acute toxicity testing in fish for GO-based materials within the Graphene Flagship. This is likely to enable the generation of more reliable data on the acute toxicity of GBMs to fish and can be used also in testing other emerging 2D materials.

Investigations on a direct intraperitoneal injection, which is not representative of a natural exposure route, have provided LD_50_ values of 175.39 μg/g for males and 2,901.2 μg/g for females for GO in adult fish (e.g., Japanese medaka, *Oryzias latipes*).^112^ The maximum body burden reported in fish (zebrafish, *Danio rerio*) following aqueous exposure to 50 μg/L GO for 2 days was 8 μg/g.^113^ However, it is likely that lethal concentrations were not reached explaining the lack of mortality. A similar zebrafish study on FLG showed maximum body burdens of 48 μg/g after 2 days with no acute toxicity (at 250 μg/L).^114^ Zebrafish can excrete materials during a depuration phase, albeit with different efficiencies according to material sizes (e.g., 30% and 95% for small and large sized FLG, respectively).^114^ However, more studies are needed to fully understand the toxicokinetics due to specific material properties that are not well understood due to a lack of quantitative techniques in fish tissues. With regards to the potential trophic transfer of GO, a body burden of 16 μg/g was reported in *Daphnia* fed with zebrafish-exposed GO. Much higher levels of GO accumulation were evidenced in lower trophic level aquatic organisms with low potential for biomagnification.^113^ Despite a lack of evidence of acute toxicity in fish to date, clear sublethal effects associated with GBM exposure in multiple species including zebrafish, *Danio rerio*,^115−118^ climbing perch, *Anabas testudineus*,^119^ common carp, *Cyprinus carpio*,^110^ geophagus, *Geophagus iporangensis*,^120^ and tilapia^121^ were reported, including pathological damage in tissues (e.g., liver, gills, intestine), metabolic disturbances, changes in oxidative stress parameters at enzymatic and genetic levels, inflammatory responses, effects on neurotransmission, as well as alterations in predator avoidance behavior. In addition, changes in the gut microbiota leading to a decrease in the abundance of beneficial bacteria and dysbiosis of bacterial community, was evidenced^122^ following chronic exposure (25 days) to GO at relatively high concentrations (0.05, 0.5, and 5 mg/L), and following a 7-day exposure to GO both at low and high doses (50 or 500 μg/L).^123^ The latter study also revealed that the aryl hydrocarbon receptor (AhR) plays a significant role in modulating the gut microbiota composition in adult zebrafish.

Fish embryos, at a sensitive life stage, have also been used to assess developmental effects following GBM exposure.^75^ In particular, the zebrafish model bridges the gap between *in vitro* models and mammalian models.^124^ In fact, significant embryo mortality was evidenced following exposure to distinct GOs even at low concentrations of 0.001 mg/L^125^ with LC_50_ values of 63 mg/L reported for other tested GO materials.^77^ These models have also provided information on behavioral abnormalities and effects on neurotransmission caused by GBM exposure^117,118^ that require further investigation. Such fish embryo models have also been used to test MoS_2_ with hatching delays, malformations and oxidative stress evidenced at 5 mg/L,^109^ and mortalities in embryos exposed to aged materials (40 mg/L).^108^ This was attributed to the release of Mo ions during the oxidative-dissolution process of MoS_2_. Thus, attention must be drawn to potential transformation processes particularly for 2D materials such as MoS_2_ that may be susceptible to dissolution and O_2_ generation. Considering the increasing production volumes of 2D materials, more information is needed on the potential effects on aquatic organisms.^126^

More recently, the utility of fish cells *in vitro* as test systems has been promoted as a predictive tool in fish acute toxicity assessment as per the OECD TG 249 RTgill-W1 fish cell line assay,^127^ and their use is likely to expand in the future for testing GO.^128^ To date, fish cell lines such as the hepatoma cell line from the topminnow fish, PLHC-1,^129^ as well as the carp leukocyte cell line, CLC^128^ and bluegill sun fish *Lepomis macrochirus* BF-2 cell line^130^ have been used for testing GBMs with cytotoxicity reported at concentrations ≥40 mg/L and IC_50_ values of 122 mg/L for GO. Early studies provided evidence of uptake of GO in PLHC-1 and mechanistic information on the effects at the cellular level.^129^ A recent publication has evidenced a strong stimulation of the AhR-dependent cytochrome P4501A (Cyp1A) in rainbow trout liver cells (RTL-W1 cell line) after exposure to GO, providing clear evidence of a role of the AhR and Cyp1A system in the cellular metabolism of GO and that GO could modulate the toxicity of environmental pollutants.^131^ Subsequent studies have expanded the use of cells *in vitro* to primary cultures (e.g., hepatocytes isolated from rainbow trout, *Oncorhynchus mykiss*); however, GO was not taken up.^132^ The authors attributed this to differences in culture conditions, highlighting the requirement for harmonized test protocols. Indeed, efforts are needed to develop or adapt standardized test protocols for nanomaterials including 2D materials, and this issue is addressed in further detail in the section Regulatory Perspectives on 2D Materials.

## Impact on Cyanobacteria and Algae

**Figure 6:**
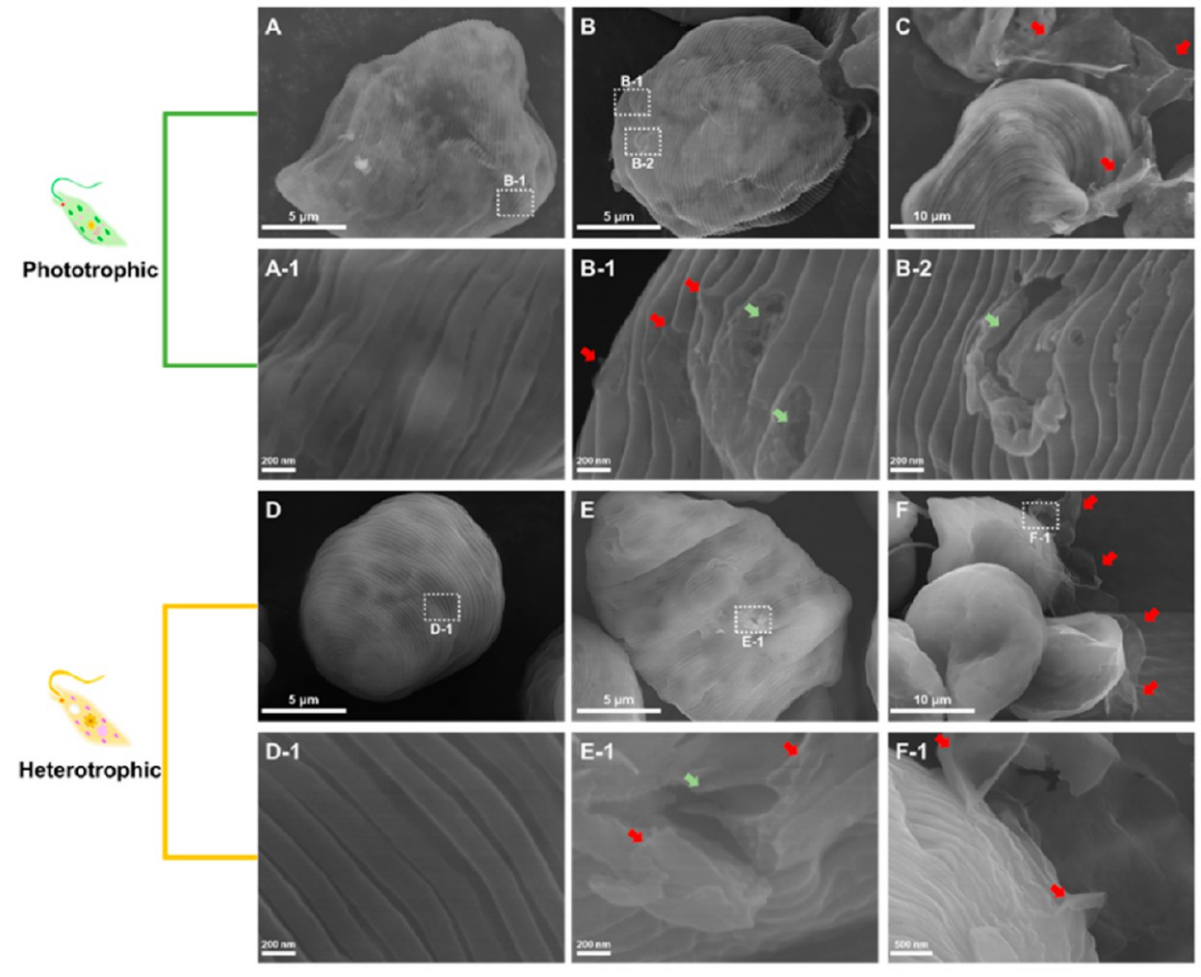
SEM images of GO-exposed *E. gracilis* cultivated under phototrophic (A–C) or heterotrophic (D–F) conditions. (A) and (D) are control cells (without GO), (B) and (E) are nanosize GO-exposed cells, and (C) and (F) are microsize GO-exposed cells. Red arrows indicate GO, and gray arrows indicate the damage to the pellicle structure.^135^ Reproduced with permission from Kim, K. Y.; Kim, S. M.; Kim, J. Y.; Choi, Y. E. Elucidating the Mechanisms Underlying the Cytotoxic Effects of Nano-/Micro-Sized Graphene Oxide on the Microalgae by Comparing the Physiological and Morphological Changes in Different Trophic Modes. *Chemosphere* 2022, *309*, 136539. Copyright 2022, Elsevier.

Photoautotrophic organisms are at the base of trophic webs, being a major source of oxygen and organic matter in both aquatic and terrestrial environments, and for this reason, particular attention was paid to verify the potential adverse effects of 2D nanomaterials. Freshwater cyanobacteria, unicellular green algae, and diatoms have received most attention because they are the target organisms of standard guidelines (e.g., OECD), used to test the potential effects of chemicals on the aquatic environment. From the initial work addressing the environmental hazards of GBMs,^133^ attention was gradually narrowed down to GO as it is considered the most toxic GBM due to its reactivity and (relative) stability in aqueous suspensions. To predict the effects of GBMs under conditions more similar to those in the natural environment, the copresence of GBMs and other natural or anthropogenic substances and contaminants was verified and tested. 2D nanomaterials alternative to GBMs have also been considered. Recent literature on cyanobacteria and freshwater microalgae confirms previous findings^133,75^ on the effects of GBMs, particularly GO at concentrations of 5 to >50 mg/L. The effects include induction of oxidative stress due to internalization of the flakes into the cell,^134^ physical damage to cell membranes due to the extreme hardness and low thickness of flakes,^135,87^ and shading due to agglomeration of 2D nanomaterial particles with the cells,^136,137^ although agglomeration is not a toxicity mechanism. Despite the large consensus on these effects, they were not always confirmed even when similar GO concentrations were applied.^138,139^ These differences could be due to different exposure modalities or physiological characteristics of the target organisms. Because of the latter factor, effects may vary. For example, *Chlamydomonas reinhardtii*, *Microcystis aeruginosa*, and *Cyclotella sp*. were less susceptible to exposure to GO at 10 mg/L than *Chlorella vulgaris* and *Scenedesmus obliquus*.^136^ The authors suggested that the lower susceptibility depended on the ability of the species to move in the water column conferred by flagella (*C. reinhardtii*) or buoyancy organelles (*M. aeruginosa* and *Cyclotella sp*.), which attenuate the shading effect due to GO agglomeration. In addition, cell wall composition^136,140^ and cell wall thickness^138^ can significantly affect the chances of direct physical damage as well as internalization of small flakes. Trophic lifestyle can also influence GBM toxicity. Some unicellular green algae can pass from an autotrophic to a heterotrophic lifestyle depending on the environmental conditions. It was shown that the green alga *Euglena gracilis* was more susceptible to GO-induced oxidative stress when it grew under photoautotrophic conditions than when it grew under heterotrophic conditions.^135^ The authors also found size-dependent effects insofar as nanosized GO was internalized by cells via endocytic activity/piercing, whereas micron-sized GO attached to the cell surface but did not enter the cells (Figure 6).

The conditions in nature are much more complex than those adopted in the laboratory using test guidelines or well-established protocols. Freshwater environments can be potentially rich in dissolved and suspended NOM. The use of these substances is rarely considered, or even discouraged, in test guidelines for testing the toxicity of substances to algae. However, studies using standard humic acids (HA) to simulate the presence of NOM in water found that HA consistently mitigated the toxic effects of GO,^72,141,142^ amine- and carboxy-functionalized GO,^142^ rGO,^141^ and graphene^141^ on various species of freshwater green algae, such as *Chlorella pyrenoidosa*,^141^ and *S. obliquus*.^72^ These studies revealed that the mechanism underlying the mitigation effect involves decreased ROS production and mechanical damage by GBM flakes, which is due to a decreased interaction between the organisms and the materials. HA can be adsorbed on the surface of GBMs, which could alter the surface charge of the material. Moreover, HA significantly increased micronutrients availability, particularly Mg and P, with promoted algal growth.^141^ In addition to natural substances, freshwater environments can be also contaminated by substances or nanoparticles of anthropogenic origin. Mitigation of the toxic effects was observed when *C. reinhardtii* algae were simultaneously exposed to GO and wastewater containing antibiotics, derived metabolites, and sweeteners^143^ in the concentration range of ng/L or μg/L. Compared with exposure to GO or wastewater, the production of ROS and membrane peroxidation were significantly decreased. In this case, adsorption of contaminants on the surface of GO as well as enhanced aggregation of GO were considered to be the main factors causing an antagonistic effect leading to toxicity mitigation.^144^ Mixture effects (coexposure to more than one toxicant) should also be considered. *Chlorella pyrenoidosa* and *S. obliquus* were exposed to mixtures of nano-ZrO_2_ particles and graphene nanoplatelets (GNPs) or rGO,^145^ or Zn-NPs and GO,^77^ respectively. The mixtures almost always had a stronger toxic effect than the individual particles, which was mainly due to increased oxidative stress via the accumulation of ROS. Only rGO was more toxic than the mixture with nano-ZrO_2_.^145^

The interaction of 2D materials with multiple organisms simultaneously (e.g., reconstructed fractions of trophic webs) has been rarely investigated but could be useful in predicting the consequences of the effects observed in individual species at a larger organization scale. With respect to freshwater algae, a biofilm of the diatom *Nitzschia palea* and a bacterial community were exposed to suspensions of GO and rGO.^102^ Interestingly, the materials had a different effect on the alga in respect to the bacterial community, and algal growth was not negatively affected by the materials. The bacterial community suffered from strong growth inhibition by GO, and to a lesser extent, by rGO. Furthermore, the bacterial community structure was altered only by GO at a concentration of 10 mg/L, with a significant decrease in *Protobacteria* and increase in *Bacteroidota*, which constituted more than 99% of the bacterial community. The authors suggested that extracellular polymeric substances (EPS) produced by the diatom and forming the biofilms could change the interaction modalities of the materials with the organisms. Another study in *Nostoc flagelliforme* found that GO, graphene and two types of multiwalled carbon nanotubes (MWCNTs) altered monosaccharide compositions and functional groups of exosaccharides, and improved cellular superoxide dismutase and catalase activities.^146^

The study of the potential impacts of 2D materials on freshwater algae has also been extended to TMDs. MoS_2_ tested on *C. vulgaris* at 1 mg/L resulted as toxic, as it inhibited its growth by causing a decrease in chlorophyll *a* content and an increase in oxidative stress and membrane damage.^147^ However, in a more recent study, the same authors showed that the two phases of MoS_2_ (i.e., 1T-MoS_2_ and 2H-MoS_2_) had different toxicities to the algae, with the former being more toxic than the latter.^148^ Similar results were obtained for WS_2_.^149,150^ The metallic phase is characterized by a higher electron conductivity and a higher electron separation efficiency leading to a higher ability to generate oxygen radicals when irradiated with visible light.^149^ Similar to GBMs, the size of MoS_2_ also matters: single-layer MoS_2_ with maximum dimensions in the range of nanometres were less toxic than those in the micrometres because they degraded more rapidly in the growth medium with algae.^148^ Another factor affecting the toxicity of MoS_2_ was the presence of S vacancies in the lattice, which can be engineered or result from dissolution and biodegradation processes.^151^ It has been demonstrated that the presence of S vacancies in single-layer 2H-MoS_2_ increases the toxicity of the material to algae, as it could harvest proteins with a high content of thiol groups, such as those involved in the antioxidant machinery, the photosynthetic apparatus, and the cytoskeleton.^152^ Again, toxicity of MoS_2_ may be influenced by compounds naturally present in the environment. Similar to GBMs, HA could mitigate the toxic effects of MoS_2_, while natural nanocolloids and EPS could enhance its toxicity, although all natural compounds increase photodegradation of the material upon irradiation with visible light.^153,154^ Importantly, there was no toxicity of the degradation byproducts of MoS_2_, such as MoO_4_^2–^.^154^ These results show that these emerging 2D materials can have similar toxic effects on algae as carbon-based materials, but they also reveal different mechanisms resulting from their different chemical composition and consequent behavior in the environment. Importantly, they also show that the potential effects of 2D materials in freshwater environments could be very different from those reported using standard test guidelines such as OECD TG 201, for ecotoxicity testing on algae.

## 2D Material Impact on Plant Reproduction

The effects of 2D materials on photoautotrophs have also been studied in seed plants, mainly using GO, although a few studies have explored TMDs. The results are largely consistent with those of previous studies^3,133^ although different life stages, exposure modalities, and concentration ranges were used. The effects of 2D materials on seed germination and seedling development have often been tested using simple standard protocols. At the highest GO concentrations tested, (i.e., 2000 mg/L and 10 mg/L) reduced germination rate and increased frequency of mitotic events and DNA aberrations in seedling meristems were observed in wheat (*Triticum aestivum*)^155^ and rice (*Oryza sativa*).^156^ Root development also appeared to be negatively affected by certain GBMs. GO (10 mg/L) negatively affected root growth of *O. sativa*,^156^ and resulted in ion loss and oxidative imbalance, possibly due to an endocytosis process of GO, in roots of pea seedlings (*Pisum sativum*) treated with ^13^C-labeled GO up to 2000 mg/L.^157^ The interaction between GO and plant roots was also studied^158^ by growing wheat seedlings over sponges impregnated with GO dispersions (10 to 800 mg/L). GO flakes were observed in the vacuoles of the cells of the root tip, meristem, and elongation zone. Notably, GO was associated with a decrease in nitrate content in shoots and especially in roots. These results are consistent with apple (*Malus domestica*) plants in GO-enriched medium, where GO at the highest concentration of 10 mg/L inhibited lateral root formation and reduced adventitious root elongation. Expression of genes related to lateral root and root hair formation and auxin response were stimulated at 0.1 mg/L and inhibited at 10 mg/L.^159^ It is difficult to envision that the root system of a plant is exposed only to 2D materials without being coexposed to other organic and inorganic xenobiotics. Several studies have investigated the adverse effects on plants of GO in combination with cadmium, a known phytotoxic element. One study highlighted an increased cellular oxidative imbalance due to higher influx of Cd^2+^ into the roots, whereas GO alone showed very low toxicity at concentrations higher than 10 mg/L.^160^ The GO-enhanced influx of Cd^2+^ into the plant has also been observed in other studies with different species (e.g., rice seeds and duckweed *Lemna turionifera*), GO types, and concentrations.^160−162^ Interestingly, all these studies showed increased membrane permeability at the root level when GO and Cd^2+^ were present simultaneously. In contrast, another study^163^ showed that GO at concentrations up to 200 mg/L did not increase Cd^2+^ influx at the root level in rice seedlings, whereas at 400 mg/L it could inhibit influx, likely depending on downregulation of genes encoding Cd^2+^ plasma membrane transporters.^158,163^ The different results seem to depend on the GO, which was at least three times thicker than that used in previous studies.

Passive translocation from roots to leaves through the vascular tissue (xylem) of seed plants is a known process.^164^ Recently, translocation of rGO to leaves was observed in seedlings of *P. sativum* where gradual inhibition of photosynthesis occurred with increasing rGO concentration.^157^ A decrease in chlorophyll *a* content of wheat (*Triticum aestivum*) leaves was also observed in plants cultured with GO suspensions at 2000 mg/L.^155^ Simultaneous exposure to 2D materials and other potentially toxic elements may lead to enhanced adverse effects. For instance, decreased efficiency of photosystem II and decreased levels of chlorophylls, carotenoids, and ribulose-1,5-bisphosphate carboxylase/oxygenase, with a subsequent decrease of net photosynthesis, was observed in wheat seedlings exposed to GO at 5, 10, 20, and 40 mg/L enriched with Cd^2+^.^160^ TMDs were also tested for their potential effects on plants. Six-day-old seeds were nebulized with MoS_2_.^165^ Seed germination was not affected by MoS_2_, while seedlings showed a concentration-dependent increase in root and shoot growth, chlorophyll content in leaves, and overexpression of a gene encoding an aquaporin in roots. MoS_2_ was also found in leaves. Potential effects of WS_2_ were tested on rice seedlings by exposing them to soil enriched with nanomaterial at different concentrations (10 and 100 mg/kg).^150^ The highest affected root development and induced an oxidative imbalance that caused membrane peroxidation and reduced overall antioxidant capacity of the seedlings. WS_2_ also altered the chemical and bacterial microflora of the soil, lowering soil pH and increasing the bioavailability of extractable phosphorus and micronutrients such as Cu, Fe, and Zn.

The study on potential effects of 2D materials has been extended to sexual reproduction. This biological process is of central importance to most terrestrial ecosystems, but also to human society, as the yield of crops, largely consisting of fruits, seeds, and their derivatives, is fully dependent on it. This process takes place in flowers and is controlled by the interaction between the pollen and the pistil. The effects of FLG, GO, and rGO on pollen of hazel (*Corylus avellana*, anemophilous) and tobacco (*Nicotiana tabacum*, entomophilous) plants were studied.^166^ Pollen was exposed to increasing GBM dispersions and pollen germination and pollen tube elongation were evaluated. GO had a dose-dependent negative effect on pollen performance, mainly due to its acidic properties. GO also adsorbed Ca^2+^ from the germination medium, which further affected pollen performance. FLG caused only reduced pollen germination, while rGO had no effect, possibly due to the very limited dispersibility of this GBM in aqueous media.

**Figure 7:**
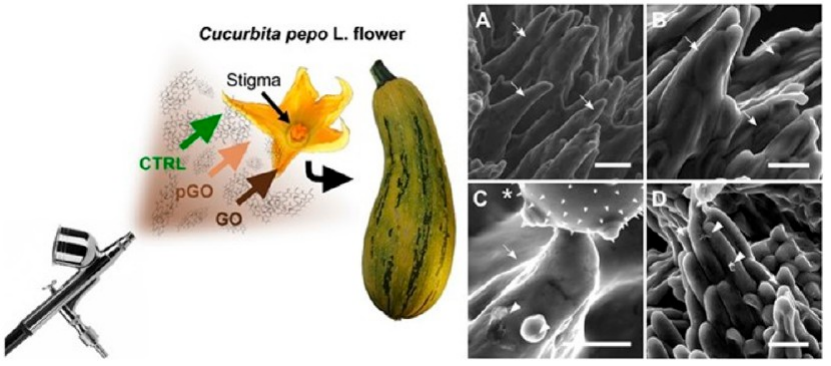
Ecotoxicology of 2D materials: interactions of GO with the sexual reproduction of a model plant (summer squash). Experimental design (left) and SEM micrographs (right) of stigmas of *Cucurbita pepo* flowers treated with dry depositions of 0 (CTRL) (A,B) or 22 μg/mm^2^ of GO (C) or purified GO (pGO) (D) and pollinated after 3 h. Stigmatic papillae, GO flakes/nanoparticles, and pollen grain are indicated with arrows, arrowheads, and asterisk, respectively. Scale bars = 100 μm.^169^ Reproduced with permission from Zanelli, D.; Candotto Carniel, F.; Fortuna, L.; Pavoni, E.; Jehová González, V.; Vázquez, E.; Prato, M.; Tretiach, M, Interactions of Airborne Graphene Oxides with the Sexual Reproduction of a Model Plant: When Production Impurities Matter. *Chemosphere* 2023, *312*, 137138. Copyright 2023, Elsevier.

The success of sexual plant reproduction relies on several key events. One of them is the correct interaction between pollen and stigma (i.e., the surface at the tip of the pistil that provides optimal conditions for pollen germination). The potential effects of GBMs deposited on the stigma surface was verified by treating the stigma of female flowers of squash marrow (*Cucurbita pepo*) with FLG, GO, and pGO (a GO purified from production process residues), and muscovite (MICA), a naturally occurring nanoparticle.^167,168^ All GBMs and MICA reduced pollen adhesion to the stigma and pollen germination without significantly affecting stigma integrity,^167^ suggesting that GBMs are as hazardous to the pollen-stigma system as nanoparticles commonly found in soil dust. However, both GO and pGO also affected fruits developed from treated flowers,^167^ although the concentrations tested were too high to be realistic. This negative effect was therefore further verified on *C. pepo* flowers using an exposure method that allowed obtaining dry air depositions of GO and pGO in the same range as daily depositions of particulate matter in highly polluted environments (5.5–22 ng/mm^2^) (Figure 7).^169^

Neither material affected the pollen-stigma system nor fruit development. However, deposition of GO significantly reduced the density and germination of seeds developed from treated flowers. Elemental analysis of GO and pGO dispersions revealed that GO contained high levels of residues from the production process that are potentially phytotoxic, especially Mn. This suggests that the impairment of seed formation may be due to the GO-enhanced influx of production residues, with subsequent damage at the cellular level, as discussed previously.

Based on the assumption that GBMs can be dispersed in the air due to their low weight and geometry, wind-pollinated plants would be most affected by airborne GBMs. This is because female flowers are morphologically adapted to intercept airborne pollen and, indirectly, other particles. Moreover, wind-pollinated flowers can remain exposed to the air for days or even weeks without losing their full receptivity. For these reasons, the uptake of GO and pGO from the air by flowers of wind-pollinated plants and if it could affect their sexual reproduction.^170^ The authors exposed female flowers of hazel, holm oak (*Quercus ilex*), walnut (*Juglans regia*), and maize (*Zea mais*) to air with a GBM concentration of 3.7 ng/m^3^ in a simulated gravity deposition. The stigma surfaces of all species were able to capture and retain the flakes. The presence of GO or pGO reduced pollen adhesion only in the flowers of *Q. ilex* and *J. regia*. In all cases, no damage to the stigma surface or reduction in pollen germination was observed, even when stigmas were wetted.

The aforementioned work on the potential effects of GBMs on seed plant sexual reproduction highlights an unexplored area of environmental impact of nanomaterials and demonstrates that GO could have adverse effects at environmentally relevant concentrations. Considering that these impacts are most likely from GO production residues, mitigation strategies could be adopted, such as applying a safe by design approach that includes increased efforts to produce cleaner materials. Regarding the more general effects of 2D materials on plants, it should be noted that most of the adverse effects stem from exposure to GBMs at very high concentrations. These are unlikely to occur under real-world conditions when predictive models^171^ and recent results on GBM biodegradability are considered (see next section). Therefore, GBMs will be safe for plants at expected emission levels, although more data need to be collected on the process of sexual reproduction and on testing 2D materials alternative to GBMs.

## Fungal and Bacterial Degradation of 2D Materials

The biodegradability of engineered (nano) materials is central to predict environmental fate, regardless of voluntary release^172^ during their life cycle. Some 2D materials are indeed resistant and reactive and could be harmful to biota, as in the case of some GOs and rGOs.^133^ Thus, understanding the extent of biodegradability, and how 2D materials and their byproducts interact with biota can help predict short- and long-term impacts and propose mitigation strategies. Biodegradation of 2D materials in mammalian systems is discussed under “Biotransformation of 2D Materials”.

Primary decomposers such as fungi and bacteria break down organic material releasing complex mixtures of oxidizing enzymes and molecules. The mixtures released by wood-degrading fungi, such as white rot fungi, are even capable of degrading lignin, one of nature’s most complex and recalcitrant molecules, as well as persistent pollutants such as polycyclic aromatic hydrocarbons, polychlorinated biphenyls, and dioxins.^173^ Fungi are considered the most effective decomposers because their mycelia can penetrate substrates and degrade them from the inside out.^174^ The biodegradability of 2D materials has been studied *in vivo*, using fungal cultures. rGO was incubated in cultures of the white rot fungus *P. chrysosporium* for up to 28 days.^175^ The authors used a culture medium that stimulated the production of Mn-peroxidase (MnP) but not lignin peroxidase (LiP), and characterized enzyme activity. After incubation, rGO was enriched in oxygen and the flakes had a statistically significant higher amounts of defects as well as holes in the graphene lattice. Both MnP and laccase were active during the incubation period, suggesting that the fungus was able to oxidize graphene by enzyme-generated radicals. More recently, FLG was incubated for four months in liquid cultures of two white-rot fungi, basidiomycetes *P. chrysosporium* and *Bjerkandera adusta*, and one saprotrophic fungus, the ascomycete *Morchella esculenta*.^176^ The two white rot fungi produce LiP, laccase and other enzymes, while the ascomycete does not produce LiP. FLG was found to be oxidized to a GO-like material. The results were fully compatible with environmental conditions created by the fungi during incubation: fungi acidified the medium to levels optimal for activity of the degradative enzymes and released H_2_O_2_, the main substrate of the degradative enzymes. Importantly, this work showed that the ascomycete was also capable of oxidizing FLG, albeit to a lesser extent than the other two fungi. The authors concluded that laccases, which are released by bacteria, fungi, and plants, may play an important role in FLG oxidation. In a recent study, the same authors exposed GO to liquid cultures of *P. chrysosporium*. Raman spectroscopy characterization of GO flakes after 1, 2, and 4 months of incubation showed a consistent increase of oxidation of the graphene lattice. Interestingly, LiP (but not laccases) was inactive during incubation in all cultures enriched with GO. This result was verified by incubating GO with LiP according to a previously developed *in vitro* approach.^177^ Both the lack of accumulation of byproducts and the absence of graphene lattice oxidation alluded to possible inactivation of LiP by GO by nonspecific adsorption. Conversely, there was evident GO-degradative activity and signs of GO oxidation with laccase.^178^

The role of bacteria in biodegradation processes of 2D materials has also been investigated. GO was incubated for 20 days in liquid cultures of a bacterial strain of *Labrys sp.*, selected for its ability to use graphitic materials as its sole carbon source for nutrition.^179^ GO gradually lost oxygen functional groups, and holes were observed in the graphene lattice. The authors concluded that degradation was due to reductive processes caused by the bacteria. Importantly, the degradation products were characterized as aromatic compounds with the structure of benzoic acids and phenols. Gene expression analysis revealed that 644 genes were up- or down-regulated in the bacterial culture incubated with GO. Most of these genes coded for proteins that were components of specific pathways for benzoate, naphthalene, caprolactam, and xylene degradation, as well as pathways leading to oxidative reactions and carbon ring cleavage. In addition, bacteria from the gut microbiome of detritivores have been implicated in the biodegradation of 2D materials. Larvae of the insect *Tenebrio molitor* (mealworm) were put in contact with a 1.5 × 1.5 mm GO film deposited on the bottom of the growth vessels.^180^ After 15 days, the larvae had consumed the GO film, and residues of GO with holes, defects and higher oxygen content were found in their feces. Remnants of GO were still present in the larval gut. The main cause of GO degradation was the gut microbiome, as shown by incubating GO with larval homogenates. The authors demonstrated the presence of degradation products such as 5-formyl-2-hydroxybenzoic acid and 2-(naphthalene-1-ylmethylene)-succinic acid.

Thus, evidence to date suggests that GBMs, regardless of their physicochemical properties such as C/O ratio and shape, can slowly be degraded by different microorganisms sharing similar degradative mechanisms/pathways, as well as by detritivores (indirectly via the microbiome). This likely precludes long-term accumulation of GBMs in the environment by accidental or direct release of graphene-containing compounds at least at the amounts predicted so far.^171^ However, nothing is known about the effects and fate of the degradation products of GBMs in the environment.^133,3^ GBMs could also interact with organic matter (e.g., humic substances) to form a so-called eco-corona, which can alter surface reactivity of the materials toward organisms. In this context, a recent study on the 2D material MoS_2_ showed that EPS released by algae promoted the formation of sulfur vacancies and pores in the MoS_2_ lattice under simulated visible-light irradiation.^154^ Compared to pristine MoS_2_, MoS_2_ with a “corona” of EPS exhibited stronger developmental inhibition and photosynthetic toxicity on the microalgae, *Chlorella vulgaris*.

## Human Health Impact of 2D Materials

**Figure 8:**
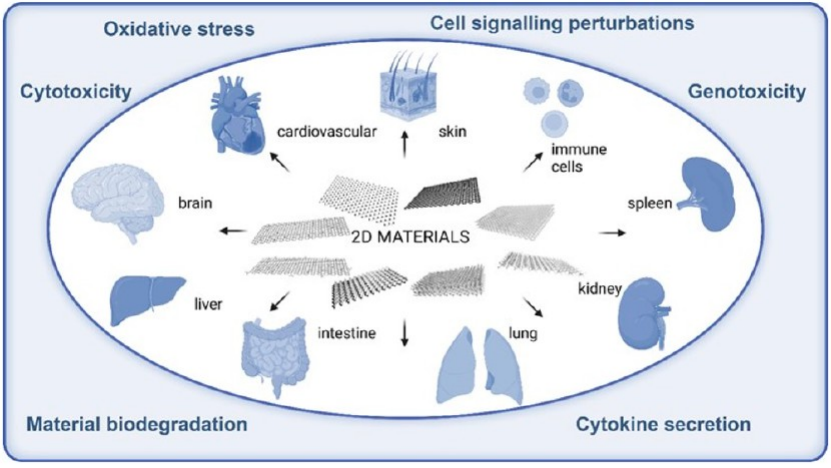
Illustration of 2D materials that can potentially come in contact with the different barriers and organs of living organisms and their possible effects.

Given the potentially wide-range effects of 2D materials on human health,^3,181^ a detailed assessment of the impact on key target organs in the body following oral, dermal, inhalation, or parenteral exposure is required. Figure 8 displays the different types of 2D materials (i.e., GO, rGO, FLG, graphene, MoS_2_, hBN, MXene, and black phosphorus) and the organs and the biological barriers that they can target or encounter in a living body, described in this section, together with their possible effects. Here, we discuss the findings related to the skin, the lungs, the immune system, the gastro-intestinal tract, the liver, spleen, and kidneys, the cardiovascular system, the reproductive and developmental systems, and the central nervous system. Our previous review^3^ focused on GBMs, while the present discussion provides an update on GBMs as well as other 2D materials, including TMDs and hexagonal boron nitride (hBN), with emphasis on results published during the past 5 years.

## Impact on the Skin

Safety issues of 2D materials for human health are mainly associated with occupational exposure during manufacturing, and cutaneous contact is certainly one of the most important exposure routes.^182^ In addition, technological applications implying skin contact are already available for some GBMs, e.g., skin-mountable biosensors and for skin regeneration purposes.^183^ Beyond GBMs, several other 2D materials are currently being explored such as TMDs (MoS_2_ and WS_2_), MXenes, and black phosphorus. These materials can be incorporated into skin-mountable devices, such as tactile and touch sensors, electrophysiological and/or electrochemical sensors, implantable biosensors, and advanced displays to improve their performance.^184^ Moreover, the application of 2D materials in tissue engineering and regenerative medicine has been gradually developed, including at the skin level.^185^ Overall, cutaneous contact is probably the most underestimated exposure route, both with respect to occupational and/or voluntary (wearable and implantable devices) scenarios.^183^ For GBMs, a good knowledge has been gained regarding their safety at the skin level.^3^ However, there is currently a paucity of data for other 2D materials, limited to hBN, MoS_2_, and black phosphorus.

### Graphene-Based Materials

**Figure 9:**
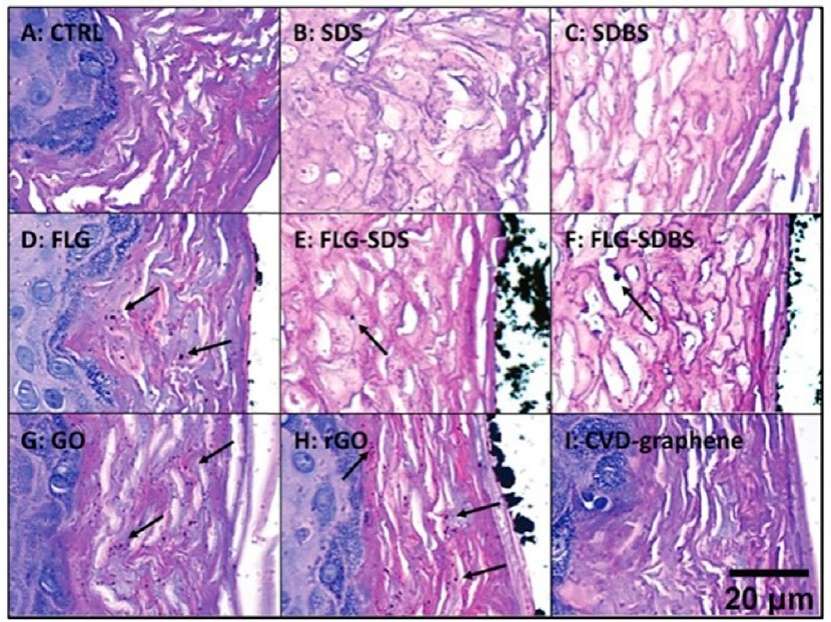
Skin irritation test using the SkinEthic^TM^ reconstructed human epidermis, following the Organisation for Economic Co-operation and Development (OECD) Test Guideline (TG) 439. Presence of GBMs above the epidermis surface and within the stratum corneum (shown by arrows) in reconstructed human epidermis (RhE) exposed to vehicle (A), sodium dodecyl sulfate (SDS) (B) and sodium dodecylbenzenesulfonate (SDBS) (C) positive controls, FLG (D), FLG-SDS (E), FLG-SDBS (F), GO (G), rGO (H), or chemical vapor deposition (CVD) (I). Scale bar: 20 μm.^188^ Reproduced in part with permission under a Creative Commons 3.0 Unported License from Fusco, L.; Garrido, M.; Martin, C.; Sosa, S.; Ponti, C.; Centeno, A.; Alonso, B.; Zurutuza, A.; Vazquez, E.; Tubaro, A.; Prato, M.; Pelin, M. Skin Irritation Potential of Graphene-Based Materials using a Non-Animal Test. *Nanoscale* 2020, *12*, 610–622. Copyright 2020, the Royal Society of Chemistry.

GBMs have been extensively studied with respect to skin effects in the frame of the Graphene Flagship using mainly *in vitro* model systems of the human skin.^3^ However, few *in vivo* data on skin adverse outcomes induced by GBMs are currently available. Recently, two independent studies investigated the skin sensitization potential of GNPs,^186^ as well as FLG and GO,^187^ following the OECD TG 442B. The initial study applied a protocol using female BALB/C mice exposed to GNPs for three consecutive days. The stimulation index (SI) value, below the threshold predicting skin sensitization, suggested GNPs as a nonsensitizer material.^186^ The second study adopted a protocol using female CBA/JN mice exposed to FLG (average size: 171 nm) or GO (average size: 15 μm) for three consecutive days. SI values for FLG and GO were also below the skin sensitization threshold. In addition, both FLG and GO induced no signs of skin irritation and inflammation, despite their capability to slightly penetrate epidermis or dermis.^187^ The lack of skin irritation and sensitization properties was corroborated also by *in vitro* studies. In particular, the irritation potential of a panel of GBMs [FLG exfoliated with melamine, FLG exfoliated with sodium dodecyl sulfate (SDS), FLG exfoliated with sodium dodecyl-benzenesulfonate (SDBS), chemical vapor deposition (CVD)-graphene films, GO, and rGO] was assessed following the OECD TG 439, using SkinEthic^TM^ (reconstructed human epidermis) as a fully differentiated three-dimensional epidermal tissue constituted of normal keratinocytes. Among the tested GBMs, only FLG exfoliated with SDS (average size: 917 nm) or SDBS (average size: 1097 nm) resulted as irritants. However, the effects were ascribed to the high amounts of residual surfactants in the final materials rather than to the materials themselves. Indeed, after removal of the residues by repeated washings, the same materials resulted as nonirritant, similar to FLG exfoliated with melamine. On the whole, these results demonstrated that GBMs exfoliated with nontoxic agents, or those from which toxic agents had been fully removed, can be viewed as nonirritant. Notwithstanding, for FLG, GO, and rGO, histological analysis revealed the presence of small depots within the epidermis, especially in the stratum corneum, suggesting the possibility of GBMs to penetrate the outer skin layers (Figure 9).^188^

This observation was confirmed using human skin samples (thickness: 0.8 mm, from one healthy donor) and Franz diffusion cells: GO (average size: 197.6 nm; concentration range: 300–1000 μg/mL) permeated the skin in a time-dependent manner, with about 55% of the total GO permeating within 6 h exposure.^189^ Regarding skin sensitization, GNPs (*<*2 μm) were tested following the OECD TG 442D.^186^ The procedure evaluates the ability to activate keratinocytes *in vitro* as the second key phase of skin sensitization adverse outcome pathway (AOP). Using the KeratinoSens^TM^ model of human skin and measuring the induction of a stably transfected luciferase gene under the control of the antioxidant response element, no sensitization potential was recorded for GNPs.^186^ These results confirmed the conclusions obtained in the *in vivo* studies demonstrating that GNPs are not skin sensitizers. In support of this, a recent study using an *in vitro* 3D reconstructed human epidermis model based on OECD TG 439 attributed skin irritation to added surfactants such as sodium dodecyl sulfate and not GBMs such as rGO and GNPs.^190^ The same study also excluded skin corrosive properties for a wide range of GBMs (including FLG, GO, rGO, and GNPs) by applying the OECD TG 431.^190^

Beyond the evaluation of skin irritation, corrosion and sensitization properties of GBMs, the majority of the *in vitro* studies to define the mechanisms of acute toxicity in the Graphene Flagship were carried out on keratinocytes or fibroblasts. Studies on HaCaT skin keratinocytes revealed that the highest oxidized materials, as GO, were the most cytotoxic ones. However, cytotoxicity only manifested after long exposure (48–72 h) and with high concentrations of GBMs (>10 μg/mL).^191^ GBM internalized by cells could continuously trigger adverse effects that were partially reversible, even if with low potency.^192^ Both FLG (average size: 391 nm) and GO (average size: 979 nm) induced mitochondrial membrane depolarization and ROS production in multiple studies, and evidence was provided for a selective activation of NADH dehydrogenase and xanthine oxidase^193−195^ and free cytosolic calcium in skin keratinocytes, with a consequent rearrangement of their metabolome of the cells.^194^ Effects were higher for GO as compared to FLG, probably because of relatively high amounts of oxygen-containing functional groups.^193,194^ As a possible consequence of oxidative stress induction, further evidence demonstrated that GBMs can trigger a pro-inflammatory response in keratinocytes. Indeed, HaCaT cells exposed to subcytotoxic concentrations (0.01–1.0 μg/mL) of FLG (average size: 413.8 nm) or thermally dehydrated GO (average size: 979 nm) released significant amounts of IL-1α, IL-6, IL-8, and TNF-α. However, conditioned media collected from GBM-treated keratinocytes failed to influence monocyte differentiation and migration, supporting the lack of sensitization potential.^196^ Other recent studies have evaluated the long-term effects of some GBMs. Remodeling of the metabolome was evidenced in keratinocytes exposed for 1 week to subcytotoxic concentrations (5 μg/mL) of FLG (average size: 40 nm), showing alterations in cellular energetic metabolism along with alterations in Ca^2+^ ions and redox homeostasis.^197^ Furthermore, metabolic remodeling was also observed after 30-day exposure to FLG (average size: 0.3 μm) or GO (average size: 2.17 μm) in epithelial cells, with increased levels of tricarboxylic acids.^198^ In addition, the oxidation degree of graphene was found to determine genotoxic effects following subchronic exposure. Genotoxic effects in HaCaT cells were reversible up to 30 days with induction of DNA repair that was implicated in tumor transformation, but the effects were irreversible following a 3-month exposure.^199^ Thus, subchronic exposure is a less studied but important exposure scenario that should be considered in genotoxicity studies.

On the whole, robust data obtained in different studies carried out applying specific OECD TGs (see section Regulatory Perspectives on 2D Materials) confirm that GBMs do not display skin irritation, corrosion and sensitization properties, at least when they are prepared using nontoxic exfoliating agents or when toxic agents are fully removed from the final material. Additionally, *in vitro* data confirmed low cytotoxic potential for the majority of GBMs, but the ability of FLG and GO to induce mitochondrial damage, ROS production, metabolic alterations and release of pro-inflammatory cytokines raises some concern regarding their (long-term) safety at the skin level.

### Hexagonal Boron Nitride

Toxicity studies on the effects of hBN at the skin level are still scanty. In particular, no *in vivo* studies evaluating the cutaneous effects of this material have been reported so far. However, in 2015 the Cosmetic Ingredient Review Expert Panel published a safety assessment of boron nitrides used in cosmetics as a slip modifier,^200^ hBN was reported not to be an irritant (using 50% hBN in olive oil on 20 participants). Similarly, two eye shadow formulations and a face powder formulation containing 13–18% of hBN were nonirritant and nonsensitizing for the skin. However, all these results were supported only by unpublished data given by the Personal Care Products Council, without any detailed experimental information, including physicochemical properties of the tested materials.^200^ Early *in vitro* work on hBN, carried out on human skin fibroblasts (CCD-1094Sk) exposed to hBN (lateral size: 50–190 nm), showed no cytotoxic effects up to 100 μg/mL, with a slight cytotoxic effect observed only at the highest concentration tested (400 μg/mL).^201^ This observation was subsequently confirmed on human dermal fibroblasts, which showed a concentration-dependent reduction of cell viability by a similar hBN (50–70 nm).^202^ The results suggested a weak cytotoxic potential, in line with the effects observed in keratinocytes. Indeed, hBN induced only a slight cytotoxicity in HaCaT cells after exposure up to a concentration of about 85 μg/mL. Unfortunately, no quantitative data about hBN size and thickness were provided.^203^ Overall, the few available *in vitro* studies suggest a weak cytotoxic potential of hBN. However, further studies are needed.

### Molybdenum Disulfide

Concerning TMDs, only a few studies aimed to assess the toxic effects of MoS_2_ at the skin level are available. The only *in vivo* study evaluated the cutaneous effects of a MoS_2_ thin film (prepared via direct sulfurization of deposited Mo film on quartz plates) and MoS_2_ microparticles (not characterized) on female guinea pigs using a patch covering shaved skin up to 48 h exposure. No clinical sign of erythema, edema or ulcers were observed, suggesting very low skin toxicity for these forms of MoS_2_.^204^ However, a previous study on human dermal fibroblasts showed that 72 h exposure to chitosan-functionalized MoS_2_ nanosheets reduced cell viability, inducing cell membrane damage, ROS generation, DNA alteration, apoptosis, inflammation and altered cell metabolism.^205^ Similar alterations (loss of cell membrane integrity, oxidative stress, damages of nuclei) were seen in HaCaT keratinocytes exposed to MoS_2_ (hydrodynamic diameter: 602 nm) and its derivative counterpart that contained surface defects (hydrodynamic diameter: 713 nm). Cells were exposed to each material (0.2, 1.0, 5.0, and 25.0 μg/mL) under different conditions, i.e., short-term exposure for 24 h, and long-term exposure mimicking an occupational scenario (daily cell exposure for 5 days, 8 h per day, followed by 16 h culture in fresh media, followed by 2 days’ recovery without MoS_2_ materials). Structural defects in MoS_2_ were found to enhance cellular internalization and augment oxidative stress after 5 days. In addition, the 2-day recovery following the long-term exposure allowed only slight improvement of cell viability.^206^ Despite the test on guinea pigs not showing signs of skin toxicity, the data remain insufficient to draw conclusions on cutaneous hazards of MoS_2_.

### Black Phosphorus

Other postgraphene materials such as black phosphorus (BP) have also been considered for biomedical applications implying skin contact. For instance, washable skin-touch-actuated BP-based “nanogenerators” embedded in textiles were developed for harvesting mechanical energy from body movements.^207^ Additionally, silver-laden BP nanosheets were prepared for antibacterial applications.^208^ In a recent study, BP nanosheets functionalized with antibacterial peptides (lateral dimension estimated to be about 575 and 512 nm for BP alone and BP modified with antibacterial peptides, respectively) displayed *in vivo* antibacterial activity with >99% antibacterial effectivity in a mouse model of methicillin-resistant *Staphylococcus aureus* (MRSA) skin infection, accompanied by negligible toxicity.^209^ However, specific evaluation of potential skin effects of BP sheets is lacking.

In sum, whereas GBMs do not show skin irritation, corrosion and sensitization, skin toxicity data on other 2D materials has been insufficient to draw firm conclusions as physicochemical properties such as oxidation state can vary, which may greatly influence the toxicity. Given that the skin is the most underestimated exposure route, caution should be taken, especially in the occupational setting. In addition, subchronic exposure has been highlighted as an often-neglected time-point.

## Impact on the Immune System

**Figure 10:**
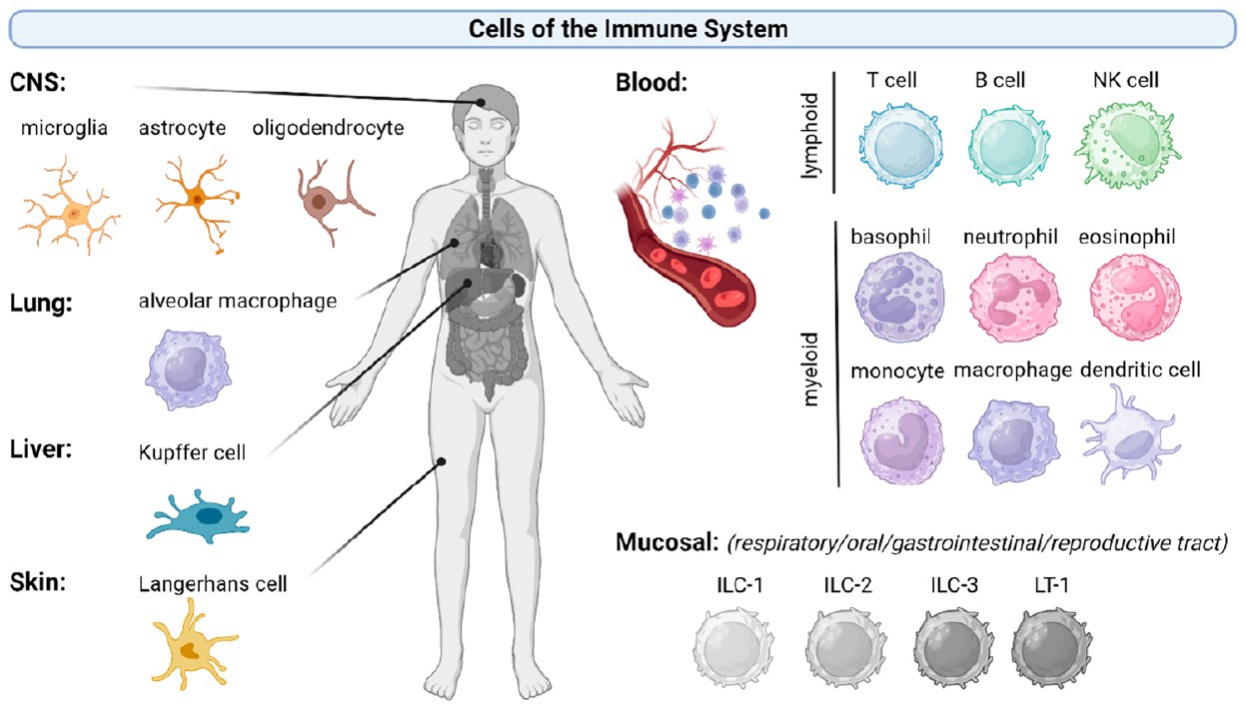
Illustration of the various organs and immune cells of the human body.

The immune system is commonly divided into the innate and adaptive arm.^210^ Innate immune cells comprise monocytes and macrophages, as well as mast cells, and granulocytes such as basophils, neutrophils, and eosinophils. Dendritic cells (DCs) serve as a bridge between the innate and adaptive arms of the immune system. Adaptive immune cells comprise lymphocytes that are classified as T and B cells, which themselves are subdivided into multiple phenotypes based on surface receptors and functions (Figure 10). Professional phagocytic innate immune cells such as macrophages, neutrophils and DCs are what foreign materials initially encounter upon contact with a physiological barrier such as the skin, or the gut or lung epithelium, depending on the route of exposure. These innate immune cells are also important in tackling pathogens or apoptotic debris.^211^ We have previously reviewed the interactions between GBMs and the immune system.^3^ We also emphasized the importance of addressing potential endotoxin contamination as endotoxins, derived from Gram-negative bacteria, can have unexpected impacts on lipopolysaccharide (LPS)-responsive pathways such as Toll-like receptor (TLR) signaling and downstream inflammatory cytokine production.^212^ It is therefore crucial to ensure that nanomaterials are free from endotoxin contamination to avoid the possibility of experimental results attributed to contamination and not to the test material.^213^

In this chapter, we discuss interactions between GBMs and other 2D materials and the innate and adaptive branches of the immune system, addressing various immune cell types. The *in vivo* impact of 2D materials (in rodent models) is covered in the subsequent organ-specific sections.

## 2D Materials and Innate Immunity

### Graphene-Based Materials

The bulk of experimental studies of GBMs published in recent years have focused on GO, which is a hydrophilic material, while some studies have also addressed graphene. Graphene was found to increase ROS production in unpolarized macrophages and increased oxygen consumption rate in unpolarized and pro-inflammatory M1 macrophages, with macrophages that take up graphene remaining viable.^214^ Moreover, graphene physical structure can affect macrophage responses.^214^ GNPs triggered expression of anti-inflammatory genes such as *ARG1*, *PTGS2*, and *CYBB* in anti-inflammatory M2 macrophages.^214^ FLG was found to increase autophagic flux and expression of the lysosomal genes *ATG5*, *CTSB*, and *CTSL* in primary human macrophages, but were not cytotoxic despite cellular uptake. FLG also increased secretion of inflammatory cytokines and ROS in M1 macrophages.^215^ However, another study in mouse bone marrow-derived macrophages showed no inflammatory effect of graphene despite cell internalization.^216^ FLG was found to trigger so-called trained immunity of bone marrow-derived macrophages with increased IL-6 and TNF-α production, but this effect was negated by incorporation of graphene within a collagen matrix.^217^ Trained immunity is a phenomenon where innate cells such as monocytes or macrophages are programmed to produce an augmented nonspecific response upon subsequent challenge with microbial products.^218^ These *in vitro* data thus suggest that GBMs could exert long-term immune-modulatory effects in the absence of cytotoxicity.

**Figure 11:**
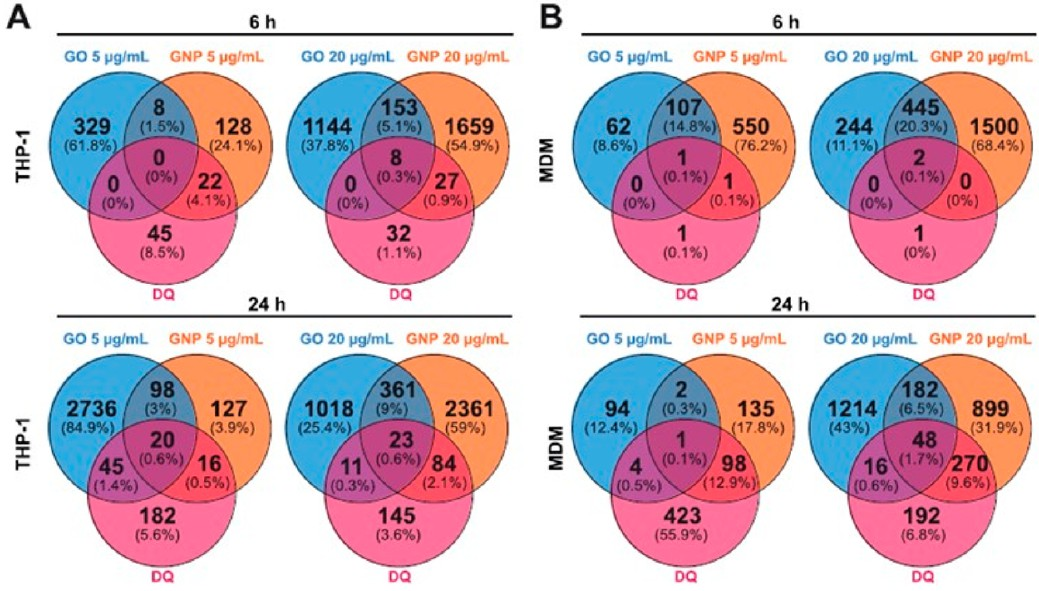
Gene expression profiling of human macrophages exposed to GO versus GNP. Venn diagrams of differently expressed genes (DEG) indicating material-specific responses in THP-1 macrophages and monocyte-derived macrophage (MDM). Comparison of DEG after 6 and 24 h of exposure to 5 or 20 μg/mL GO or GNP or 100 μg/mL DQ (crystalline quartz) in THP-1 macrophages (A) or MDM (B).^219^ Reproduced in part with permission under a Creative Commons BY 4.0 License from Korejwo, D.; Chortarea, S.; Louka, C.; Buljan, M.; Rothen-Rutishauser, B.; Wick, P.; Buerki-Thurnherr, T. Gene Expression Profiling of Human Macrophages After Graphene Oxide and Graphene Nanoplatelets Treatment Reveals Particle-Specific Regulation of Pathways. *NanoImpact* 2023, *29*, 100452. Copyright 2023, Elsevier.

Recent transcriptomics studies in the Graphene Flagship showed that GNPs mainly upregulated inflammatory and apoptotic genes in human macrophages whereas GO showed only limited inflammatory impact. The authors also highlighted that primary macrophages are more sensitive to GBMs when compared to the macrophage-like THP-1 cell line (Figure 11).^219^

GO and rGO were found to have no toxicity or effect on the inflammatory cytokine IL-8 in THP-1 macrophages.^220^ Cytokine arrays performed on human whole blood showed that GO caused overexpression of mainly monocyte and macrophage-related cytokines such as IL-6, CXCL1, CCL20, TNF-α, and CCL3, while graphene produced similar results except for IL-6.^221^ Single-cell mass cytometry performed on peripheral blood mononuclear cells (PBMCs) confirmed the complex interaction of GO with many types of immune cells, and identified monocytes, a precursor of macrophages, as the main population impacted by GO, with amino functionalization dampening immune activation.^222^ GO can impact various cell response pathways. For example, GO was found to decrease antioxidant levels and increase expression of pro-apoptotic and DNA damage genes in THP-1 cells.^223^ Proteomics analysis of GO-treated RAW264.7 macrophages showed upregulation of lipoprotein lipase and lysozyme in particular, as well as increased ROS-induced autophagy. A dose-dependent increase in membrane rafts and phagosome production was observed.^224^ Furthermore, high mobility group box 1 (HMGB1) release was observed in C57BL/6 mice and in RAW264.7 macrophages exposed to various carbonaceous nanomaterials (C_60_ fullerenes, SWCNTs, GO).^225^ Lipidomics analysis of macrophage-like THP-1 cells showed that GO reduced mRNA and proteins of the peroxisome proliferator-activated receptor (PPAR) pathway, which is related to lipid droplet biogenesis, with larger GO (500–5000 nm) having greater effects than smaller GO (<500 nm), accompanied by decreases in monocyte chemoattractant protein-1 (MCP-1).^226^ Graphene nanosheets have also been found to cause plasma membrane damage, ROS production and apoptosis in a rat basophilic cell line.^227^ It is important to note that the functionalization and/or reduction of GO can affect immune responses. For example, GO reduction decreased both oxidative stress and proinflammatory cytokines in RAW264.7 macrophages.^228^ Metabolomics of RAW264.7 macrophages confirmed that PEG-GO had minimal inflammatory potential based on decreased levels of the inflammatory metabolite succinate. PEG-GO also expressed low TNF, CD80, and CD206. These results were in contrast to flavin mononucleotide-stabilized graphene, demonstrating the different immunomodulatory abilities of various GBMs.^229^

**Figure 12:**
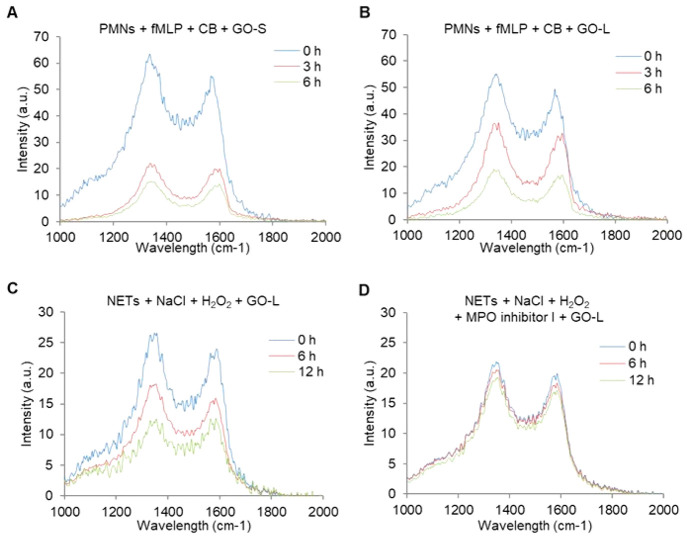
Neutrophil degradation of GO sheets with varying lateral dimensions. (A,B) Freshly isolated human neutrophils were treated with fMLP (10 nM) and cytochalasin B (5 μg/mL) to trigger degranulation and incubated with GO-S (A) or GO-L (B) for the indicated time-points. Raman confocal measurements showed biodegradation of GO-S and GO-L as determined by a reduction in the intensity of both the D and G bands. (C,D) Neutrophils were treated with 25 nM PMA for 3 h to trigger production of neutrophil extracellular traps (NETs). Then, NETs were purified and incubated with GO-L in the presence of NaCl and H_2_O_2_ for the indicated time-points and biodegradation was determined by Raman confocal microspectroscopy. Degradation of GO was evidenced in the absence (C), but not in the presence (D) of MPO inhibitor-l (0.6 μM), indicating that the acellular degradation in NETs was MPO-dependent. The data represent an average of the whole scan (10 000 spectra per sample).^230^ Reproduced with permission from Mukherjee, S. P.; Gliga, A. R.; Lazzaretto, B.; Brandner, B.; Fielden, M.; Vogt, C.; Newman, L.; Rodrigues, A. F.; Shao, W.; Fournier, P. M.; Toprak, M. S.; Star, A.; Kostarelos, K.; Bhattacharya, K.; Fadeel, B. Graphene Oxide is Degraded by Neutrophils and the Degradation Products are Non-Genotoxic. *Nanoscale* 2018, *10*, 1180–1188. Copyright 2018, the Royal Society of Chemistry.

Neutrophils are innate immune cells that respond swiftly upon encountering foreign bodies and play key roles in inflammation and host defense. Previous work in the Graphene Flagship showed that GO triggers neutrophil extracellular traps (NETs) in a size-dependent manner in primary human neutrophils (Figure 12)^230^ and this was confirmed in a recent study, in which PEG-GO was found to provoke a milder response than its unmodified counterpart.^231^ NETs are believed to be important for antimicrobial defense, but excessive formation of NETs may also contribute to tissue damage in various diseases.^232^ In another recent study conducted in the Graphene Flagship, mice were subjected to repeated chronic pulmonary exposure to GO suspensions. The authors noted a transient influx of alveolar neutrophils and eosinophils with replacement of alveolar macrophages by interstitial macrophages, for GO of different lateral dimensions, without induction of lung remodeling or adaptive immune responses. Importantly, the latter study showed that lung recovery was faster for nanosized GO as compared to micron-sized GO.^233^

Graphdiyne is an artificially produced sp^2^ and sp-hybridized carbon allotrope consisting of benzene rings and butadiyne linkages, which makes it structurally and characteristically different from conventional GBMs. No toxicity was seen in primary human M1 and M2 macrophages exposed to graphdiyne oxide.^234^ Importantly, biodegradation of graphdiyne oxide was demonstrated in M1 macrophages, which express inducible nitric oxide synthase (iNOS), but not in M2 macrophages, which lack the ability to produce nitric oxide (NO).^234^ Moreover, pro-inflammatory cytokines were produced in a biodegradation-dependent manner. Furthermore, another recent study supported the proclivity of graphdiyne oxide to polarize macrophages to pro-inflammatory M1 macrophages.^235^ Subsequent studies by other investigators have shown that graphdiyne oxide has better biocompatibility and is more susceptible to degradative oxidation than GO, following subcutaneous or intraperitoneal administration in mice.^236^ The authors also addressed biodegradation in an acellular system using hypochlorous acid, and they implied that previous work^234^ demonstrated that macrophages engulfed “carbon nanosheets” (graphdiyne oxide) into lysosomes for biodegradation, but the biodegradation of graphdiyne oxide in macrophages was shown to occur in a peroxynitrite-dependent manner.^234^

### Transition Metal Dichalcogenides and hBN

Recent studies performed in the Graphene Flagship have revealed that 2D MoS_2_ and WS_2_^237^ showed no cytotoxicity toward primary human monocyte-derived macrophages despite being readily internalized.^238^ The authors found that TMDs triggered so-called trained immunity as shown also for graphene (see above). Thus, pre-exposure to TMDs (“training”) followed by a resting period caused marked changes in immune-specific gene expression after challenging the cells with bacterial LPS. Specifically, evidence was provided for the upregulation and secretion of CD70 (also known as CD27 ligand), an important costimulatory molecule. Co-stimulatory molecules act to amplify or counteract the initial activating signals provided to T cells through T cell receptors. MoS_2_ was found to trigger trained immunity through an epigenetic pathway as seen by the reversal of effects in cells exposed to the histone methyltransferase inhibitor methylthioadenosine. Furthermore, MoS_2_ triggered an elevation of cyclic adenosine monophosphate levels in macrophages and increased glycolysis was also observed upon MoS_2_ “training”, pointing toward a metabolic rewiring of the cells.^238^ These results suggest that TMDs (especially MoS_2_) could potentially be exploited for the modulation of immune responses. Even though MoS_2_ nanosheets have been found to be internalized by macrophages, other nonphagocytic cells may respond differently. For instance, MoS_2_ nanosheets with 5-layer and 40-layer thicknesses were evaluated for their cellular effects using human lung cell lines as a model. It was observed that 40-layer nanosheets were internalized by cells, whereas 5-layer nanosheets adhered to the surface without being internalized.^239^ The authors suggested that the 2D materials could “remotely” trigger autophagy through their interactions at the cell surface, which is somewhat counterintuitive as autophagy is typically activated for the removal and recycling of damaged organelles and aggregated and misfolded proteins or for the disposal of pathogens within the cell. Moreover, not all TMDs are alike, and some authors have documented cytotoxicity for TMDs using human cell lines (BEAS-2B and THP-1). The authors prepared five 2D TMDs by exfoliating nanosheets from bulk materials of WS_2_, MoS_2_, WSe_2_, and MoSe_2_, and included also hBN in the study.^240^ They could subsequently show that MoS_2_ and WS_2_ triggered ferroptosis, an iron-dependent, lipid peroxidation-mediated form of cell death. The authors reasoned that surface vacancies were responsible for the cytotoxicity, and could show that surface passivation of MoS_2_ and WS_2_ for the “healing” of these vacancies significantly mitigated toxicity.^240^ These studies are apparently in contradiction to previous work showing that 2D nanosheets of MoS_2_ and WS_2_ showed little cytotoxicity in BEAS-2B and THP-1 cells^241^ and in A549 and HaCaT cells.^237^ However, the route of synthesis, and the dispersibility of the nanosheets, may play a key role. The dose and the exposure time also matter; indeed, it is noted that ferroptosis was observed at relatively high doses (200 μg/mL).^240^ In a very recent study, MoS_2_ nanosheets were shown to induce ferroptosis in the murine macrophage-like RAW264.7 cell line and in BEAS-2B cells through the induction of ferritinophagy and the inhibition of ferroportin-1 (FPN).^242^ Ferritin plays a central role in iron metabolism by storing iron in cells, and ferritinophagy refers to the selective autophagic degradation of ferritin resulting in the accumulation of cellular Fe^2+^. FPN is an iron export protein responsible for maintaining cellular iron homeostasis. Previous work has shown that the delivery of ferritin to the lysosomes requires the nuclear receptor coactivator 4 (NCOA4),^243^ and the authors could show that MoS_2_-triggered ferroptosis was NCOA4-dependent.^242^ Thus, some forms of MoS_2_ may trigger Fe-dependent cell death, while other forms of MoS_2_ show excellent biocompatibility. Partners of the Graphene Flagship^215^ compared FLG and MoS_2_ at doses up to 50 μg/mL using primary human monocyte-derived macrophages, which had been polarized into classically activated (pro-inflammatory) M1 and alternatively activated (anti-inflammatory) M2 macrophages. Overall, FLG and MoS_2_ showed little toxicity even though cellular stress responses were observed.^215^

Gu et al.^244^ performed molecular dynamics simulations to investigate the interactions of MoS_2_ and PEG-MoS_2_ nanoflakes with a model of the plasma membrane. MoS_2_ was found to insert and penetrate through the membrane, while the PEG chains on the surface of PEG-MoS_2_ hindered the membrane insertion process, leading to a prolonged passage through the membrane. The authors argued that this lower/prolonged membrane penetration and stronger membrane adsorption of PEG-MoS_2_ compared to nonfunctionalized MoS_2_ could explain the propensity of the PEGylated nanosheets to trigger proinflammatory cytokine secretion.^244^ These studies thus extend the previous work by the same authors on PEGylated GO, which was found to elicit stronger inflammatory responses in murine peritoneal macrophages than nonfunctionalized GO.^245^ However, it is noted that in these theoretical studies, for practical reasons, the lateral dimensions of the 2D nanoflakes are very small. Hence, the “small” and “large” MoS_2_ nanoflakes in the aforementioned study were modeled with edge lengths of 2.86 and 6.81 nm, respectively (in other words, far smaller than actual nanoflakes).^244^ Notwithstanding, these studies imply that PEGylation does not necessarily serve to “passivate” 2D materials. PEG functionalization is commonly performed in order to reduce the nonspecific protein adsorption or “corona” formation, which may occur in the blood. In a recent study, the impact of the surface adsorbed “corona” of four different proteins present in human blood, i.e., albumin, transferrin, fibrinogen, and immunoglobulin G (IgG), was investigated. The authors found that MoS_2_ nanosheets coated with IgG or fibrinogen triggered stronger responses in phorbol-12-myristate-13-acetate-activated THP-1 cells (used as a model of macrophages), and suggested that the effect of the IgG-coated nanosheets could be due to the high expression of Fc-gamma (Fcγ) receptors on the surface of macrophages.^246^ Indeed, Fcγ receptors orchestrate uptake of opsonized particles or pathogens. 2D MoS_2_ was also found to trigger NET formation in a nitric oxide-dependent manner, in human neutrophils. This effect was, however, possibly due to the molybdate ions in general, as Na_2_MoO_4_ was also reported to have the same effect.^247^

There are comparatively few studies on the impact of WS_2_ on macrophages. However, in a study using an *in vitro* coculture system of A549 and THP-1 cells to mimic the lung microenvironment, WS_2_ nanosheets were shown to trigger “bystander” effects in macrophages.^248^ Hence, when conditioned medium from A549 lung epithelial cells pretreated with WS_2_ was transferred to the macrophage-differentiated THP-1 cells, this affected macrophage responses to LPS along with a polarization toward M2 macrophages, and this was shown to occur through a nitric oxide (NO)-dependent TGF-β1 signaling pathway.

To our knowledge, there are no studies to date on hBN and neutrophils. Similarly, there are no studies, on TMDs or hBN and eosinophils, key players in allergic responses. However, a very recent study explored the interactions between basophils and MoS_2_. Basophils are also involved in inflammatory and allergic responses.^52^ MoS_2_ nanosheets manufactured according to two different methods were thus studied using primary human basophils. Overall, the analyzed materials were found to be cytocompatible. The authors noted a marginal albeit nonsignificant release of histamine (an important mediator of allergic reactions), with no impact on surface markers of cell activation or viability. The study also highlighted how components such as surfactants or exfoliating agents used in the production of 2D materials can skew the outcome.^52^ Further studies are needed to address the interactions with other granulocytes as well as mast cells, key players in allergic responses.

## 2D Materials and Adaptive Immunity

### Graphene-Based Materials

The majority of studies on GBMs and immune cells have focused on macrophages. However, as the conduit between innate and adaptive immunity, DCs play an increasingly important role in medical applications such as vaccines by inducing downstream T cell response, thus requiring more safety studies to verify biocompatibility once in contact with exogenous materials. GO in particular has previously been shown to suppress antigen presentation by DCs.^249^ Apart from being taken up solely by phagosomes, GO was shown to increase primary human DC maturation, increase production of ROS and pro-inflammatory cytokines. GO-treated DCs also induced expression of the Th1 and Treg transcription factors Tbet and FoxP3 in CD4+ T cells.^250^ Other investigators have also shown minimal phenotypic activation of murine DCs, and minimal cytokine secretion by GO of varying sizes. Of note, DCs pulsed with the model antigen protein OVA complexed with small GO (lateral size: 0.05–3 μm) could induce CD4+ T cell proliferation and FoxP3 expression, while OVA complexed with large GO (lateral size: 0.5–15 μm) could promote CD8+ T cell activation and cytokine production.^251^ Micron-sized GO (>1 μm) demonstrated strong adherence to mouse DC surface, inducing cytoskeletal reorganization via the Rho-ROCK pathway, while smaller GOs (500 nm) were mostly internalized by DCs. Micron-sized GO also facilitated DC–T-cell clusters that were crucial for T cell activation, especially in the context of acting as an adjuvant in vaccines.^252^ Factors such as the number of layers of 2D materials can affect immune responses. It was reported that commercial monolayered GO caused cell aggregation, but exerted less impact on cell viability than multilayered GO, with both GO inducing ROS in the DC2.4 dendritic cell line.^253^ A recent study showed that the different types of PEG linked to GO can play a role. Branched PEG-GO led to decreased IL-17 synthesis and an increase in PBMC-derived Th17/Th22 proportion, while linear PEG-GO increased IFN-y production. It is challenging to interpret the effect on specific T helper cells due to their potential to express markers of other T helper subsets. Th17/Th22 dual cells, for example, have been implicated in cancer, autoimmunity and infection and can transdifferentiate into Th1- and Treg-like cells.^254^*In silico* work has highlighted the mechanism of graphene immune toxicity. Graphene insertion was found to disrupt protein interactions between T-cell receptors (TCRs) and peptide-HLA, thus impairing TCR antigen recognition, leaving antigen presentation intact.^255^ Nima et al.^256^ developed an effective approach to quantify graphene interacting with single cells that utilizes combined multimodal-Raman and photoacoustic spectroscopy. Using this single-cell spectroscopic approach to study the JAWSII immature dendritic cell line, the authors could show that most cells took up graphene, supporting the observation of uptake by DCs.^256^ Using T and B lymphocytes isolated from the spleen of BALB/C mice, Murera et al.^257^ found that FLG neither impacted viability nor activation of the cells. In more recent work, amino-functionalized GO sheets were compared to GO using primary human B lymphocytes.^258^ The authors found that GO-NH_2_, in particular, triggered B cell receptor activation and upregulation of granzyme B, an important cytotoxic protein, at 50 μg/mL.

### Transition Metal Dichalcogenides and hBN

**Figure 13:**
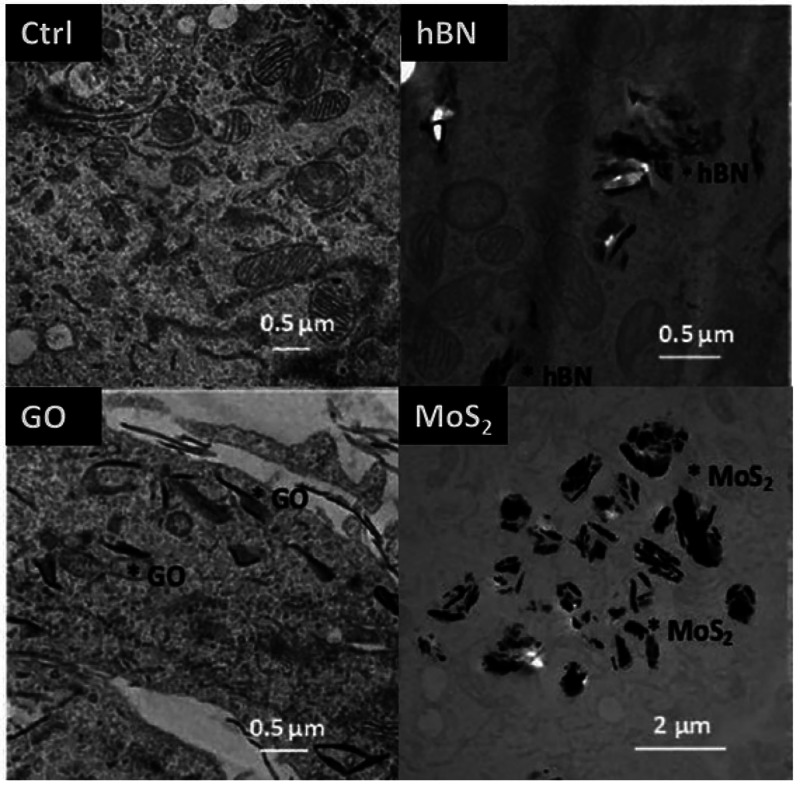
Comparative study of hexagonal boron nitride (hBN), graphene oxide (GO), and MoS_2_ using primary human monocyte-derived dendritic cells (DCs). Transmission electron microscopy (TEM) micrographs of DCs maintained in medium along (Control) or exposed to 50 μg/mL hBN, GO, or MoS_2_ for 24 h. The stars indicate the localization of the materials.^250^ Reproduced with permission from Lin, H.; Peng, S.; Guo, S.; Ma, B.; Lucherelli, M. A.; Royer, C.; Ippolito, S.; Samori, P.; Bianco, A. 2D Materials and Primary Human Dendritic Cells: A Comparative Cytotoxicity Study. *Small* 2022, *18*, e2107652. Copyright 2022, Wiley-VCH Verlag GmbH & Co, KGaA, Weinheim.

Lin et al.^250^ performed a comparative study of GO, hBN, and MoS_2_ and found that these materials did not affect the viability of primary human monocyte derived DCs at doses ranging from 5 to 50 μg/mL. Moreover, unlike hBN, MoS_2_ did not show any effect on DC maturation, and had little effect on DC-induced T cell proliferation (Figure 13).^250^ In contrast, using murine bone marrow-derived DCs as a model, other investigators have observed DC maturation at a relatively high dose (128 μg/mL) of MoS_2_.^259^ Interestingly, the *in vivo* homing of DCs was also enhanced upon exposure to MoS_2_. To this end, DCs derived from firefly luciferase-positive transgenic mice were incubated with 128 μg/mL MoS_2_, and the DCs were then injected into the footpads of recipient mice to test their capacity to drain to adjacent lymph nodes. The authors found that DCs exposed to MoS_2_ were capable of activating T cells, and an enhanced homing ability was confirmed. They subsequently concluded that MoS_2_ nanosheets are a vaccine adjuvant candidate that may fortify immune responses.^259^

The universe of 2D materials is continuously expanding. 2D metal carbides and nitrides, also known as MXenes, are one emerging class of 2D materials with several promising applications. However, few studies are available on the possible impact of these materials on immune cells. In one of these studies, the impact of MXenes was evaluated with respect to PBMCs.^260^ To this end, PBMCs exposed to Ti_3_C_2_T_*x*_ showed no evidence of cytotoxicity. However, dose–response studies were not performed. Single-cell mass cytometry^261^ is a promising tool with which to explore the impact of nanomaterials on immune cells. Previous studies using single-cell mass cytometry have focused on the impact of GO on human PBMCs^262^ and, more recently, on the impact of BP nanosheets on mouse PBMCs.^263^ However, an open question has been the detection of the materials (in cells or tissues). In a recent study, it has been reported that single-cell mass cytometry can be exploited for the label-free detection of 2D materials with single-cell or subpopulation resolution, by applying a set of MXenes (Nb_4_C_3_, Mo_2_Ti_2_C_3_, and Ta_4_C_3_). Among the tested materials, Nb_4_C_3_ displayed the strongest signal and extensive cellular interaction could be detected. In particular, DCs showed the most prominent binding to these MXenes.^264^

To sum up the literature to date, it remains challenging to conclusively determine the specific immune impact of 2D materials even within their individual subclasses due to minute differences in their manufacturing process, structure, functionalization or dispersibility profile, which could lead to various possibilities of cell interaction and downstream response. Macrophages remain the most popular immune cell of investigation due to their role as a key phagocytic cell of the innate immune system, as well as ease of culture. Nevertheless, other innate immune cell types such as monocytes, neutrophils, and dendritic cells are also being studied. Overall, 2D materials should be comprehensively studied with regards to biocompatibility and innate immune cells as well as adaptive immune cells must be considered. Furthermore, in addition to overt cytotoxicity, cell functionality must be carefully investigated.

## Impact on the Pulmonary System

Since our previous review in 2018,^3^ the impact of GBMs and 2D materials on the pulmonary system has remained one of the most studied topics, and attempts are being made to make use of the current literature to establish an AOP framework for GBM pulmonary toxicity. Here, we provide an update on the pulmonary impact of GBMs as well as other, emerging 2D materials, addressing both *in vitro* and *in vivo* studies.

### Focus on *In Vivo* Studies

**Figure 14:**
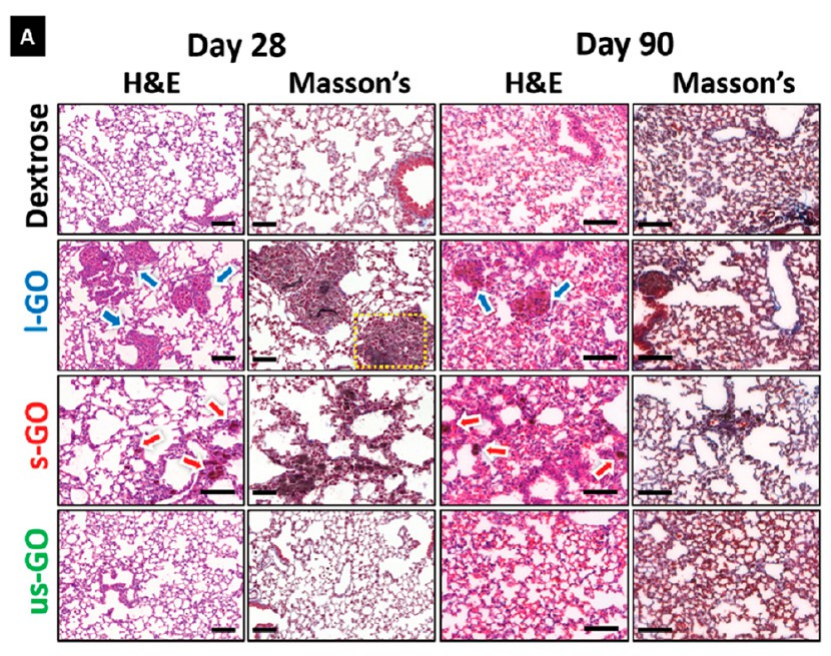
Pulmonary exposure to GO induces size-dependent granulomatous inflammation. Lung sections from mice intranasally instilled with GO were extracted at 1, 7, 28, and 90 days. (A) Representative images of sections stained with H&E and Masson’s trichrome at days 28 and 90 were acquired. Arrows indicate areas of significant immune cell infiltration in response to the presence of GO, with alveolar wall thickening and granuloma formation. Scale bars = 100 μm.^266^ Reproduced in part with permission under a Creative Commons BY License from Rodrigues, A. F.; Newman, L.; Jasim, D.; Mukherjee, S. P.; Wang, J.; Vacchi, I. A.; Menard-Moyon, C.; Bianco, A.; Fadeel, B.; Kostarelos, K.; Bussy, C. Size-Dependent Pulmonary Impact of Thin Graphene Oxide Sheets in Mice: Toward Safe-by-Design. *Adv. Sci. (Weinh.)* 2020, *7*, 1903200. Copyright 2020, Wiley-VCH Verlag GmbH & Co, KGaA, Weinheim.

Exposure of small animal models such as mice and rats to understand the pulmonary impact of GBMs has continued since our previous assessment of the state-of-the-art.^3^ Drawing from this information has allowed investigators to conduct a meta-analysis of studies focused on carbon-based nanomaterials to address the possible grouping of these materials based on patterns of inflammatory markers. Thus, data were obtained from studies in which C57BL/6 mice were exposed by oropharyngeal aspiration to 4 and 40 μg of FLG of various lateral dimensions (1 μm; 5 μm; 20 μm), GO (5 μm), or rGO (5 μm).^265^ The evaluation considered 46 proteins analyzed in the broncho-alveolar lavage (BAL) of these animals at 1 and 28 days postexposure. It was revealed that rGO was grouped with hazardous carbon nanomaterials, while FLG and GO were grouped with nonhazardous carbon nanomaterials with respect to the adverse effects on the lungs. In another series of pulmonary studies, GO sheets with different lateral dimensions were administered (in a single dose of 50 μg) to C57BL/6 mice. Using a single intranasal aspiration, only the largest GO materials (1–30 μm) cause long-term alteration of lung tissues with persistent granuloma-like structures up to 90 days, without fibrotic lesions or TGF-β upregulation and reduced translocation from the upper airways deep into the lung.^266^ In contrast, the smallest GO material (10–300 nm) showed limited to no impact on lung architecture even at the earliest time point. Midsized GO material (0.050–2 μm) presented a range of response between that of the other two GO materials. Transcriptome analysis carried out at day 28 confirmed the above results and revealed GO size specific differences in gene expression, with the largest materials triggering upregulation of five time more genes compared to the smallest materials, including some genes related to the regulation of cancer pathways. Overall, nanosized GO were cleared while micron-sized GO persisted in macrophages. This was reproduced in a recent mouse lung study where small GO (average size: 60 nm) was more easily cleared than micrometric large GO (average size: 8 μm), with similar trends observed in nanometric small FLG (average size: 200 nm) and micrometric large FLG (average size: 1 μm).^267^ In another study, when similar GO materials (60 nm compared to 8.2 μm) were given to mice thrice via oro-pharyngeal aspiration of 1 or 10 μg per animal, micron-sized GO at the highest dose were once again found to be the most inflammatory and altered lung-architecture.^233^ Moreover, repeated oro-pharyngeal administration^233^ yielded similar results as the previously performed studies with a single intranasal administration.^266^ For instance, repeated exposure induced the persistence of micron-sized GO materials in multinucleated macrophages and granuloma-like structures for up to 84 days, whereas nanosized GO induced a milder response and was cleared faster from the lungs. Importantly, no fibrosis and only innate immune response was triggered by micrometric GO. There was no evidence of adaptive or allergy-like immune response (Figure 14).^266^ In a complementary study, both nanosized and micron-sized GO induced DNA damage 1 day after single exposure to 30 μg.^268^ However, these damages were absent at day 7 and 28, suggesting the activation of DNA repair mechanisms. In the same study after repeated exposure (10 μg thrice), only micron-sized GO sheets induced persistent DNA damages at day 84. Importantly, low dose repeat exposure (1 μg thrice) that replicate possible exposure at workplace did not induce any DNA damage. The kinetics of inflammation and oxidative stress were directly associated with genotoxicity. In the case of nanosized GO, rapid recovery from DNA damage was attributed to the transient nature and rapid resolution of inflammation. In contrast, long-term DNA damage induced by micron-sized GO correlated with persistent inflammation with multinucleated macrophages and granuloma-like structures.

The role of the TLR signaling pathway was investigated using Tlr2 and Tlr4 knockout mice exposed by oropharyngeal aspiration to 18 μg micron-sized (2–3 μm) GO.^269^ While there was no major significant differences between Tlr4^–/–^ and wild-type, neutrophils influx was reduced in Tlr2^–/–^ animals. GO induction of the lung disorder biomarker serum amyloid A (Saa1 and Saa3) in the lungs was both TLR2- and TLR4-dependent, while induction of the inflammation markers Cxcl2 and Cxcl5 in the lungs was TLR2-dependent only. Expression of Saa1 and the inflammatory mediator Lipocalin-2 (Lcn2) in the liver was also TLR2-dependent. Overall, the results suggested that inflammation induced by GO was not dependent only on TLR4 signaling (also used by LPS). In another study, GO (with an average lateral size of 314 nm) was intratracheally administrated to BALB/c mice as a single bolus dose of 2.5 mg/kg.^270^ Large amounts of macrophage-dominant immune cell infiltrates, and alveolar collapse were found 24 h after exposure. Systemic inflammation, with increased IL-12 and IL-6 cytokine levels in the serum of treated animals was also reported. However, the sole time point of only 24 h does not allow for the assessment of possible recovery or induction of long-term sequelae (e.g., fibrosis). Wistar rats were exposed to large (20–30 μm) GO by single intratracheal instillation (2.5, 5, or 7.5 mg/kg), and the impact of exposure was assessed at 1 day, 3 days, 7 days, 4 weeks, 8 weeks, and 12 weeks.^271^ Regardless of dose, fibrosis was found after 12 weeks. Accordingly, increased levels of TGF-β were reported throughout all groups at all time-points. The effects thus appeared more severe than the previous findings of another group that observed lung granuloma formation with micron-sized GO (1–30 μm) but no fibrosis at 90 days.^266^

Using intravenous injection looking specifically at pulmonary impact, Sprague–Dawley rats were also exposed to GO suspensions at four doses ranging from 5 to 100 mg/kg once a day for 7 days.^272^ The two lowest dose regimes did not cause lung damage. In contrast, high GO doses led to air–blood barrier damage causing pulmonary edema and protein release in the alveolar cavity, as well as immune cell infiltration and histopathological changes. These high GO dose regimes also led to high levels of cytokine and oxidative stress markers that were associated with high levels of autophagy, measured in lung tissue. A study in nonhuman primates and mice highlighted the potential impact of GO on the lungs. To this end, the authors used male BALB/C mice (*n* = 10 for each time-point) along with adult male cynomolgus monkeys *Macaca fascicularis* (*n* = 5 for each material and *n* = 1 for control). To determine the dose of GO for nonhuman primates, the maximum safe dose for BALB/c mice was converted to the equivalent dose for *Macaca fascicularis* (i.e., maximum recommended starting dose) according to the guidance from Food and Drug Administration (FDA). After intravenous administration of the nanosized (20–80 nm) GO functionalized with amine-terminated branched PEG, death was observed in 6% of the mice 1–12 h after exposure, and in 20% (1 out of 5) of the nonhuman primates (1.5 h after exposure).^273^ Elevated serum immunoglobulin E and lung injuries (blood clots and alterations of alveolar structures) were noted, and the authors argued that the deaths were related to anaphylaxis. Anaphylaxis is a severe, life-threatening hypersensitivity reaction initiated by exposure to a specific antigen in a sensitized organism, but it is not clear whether GO itself could be considered as an immune antigen. Indeed, the cause of these deaths may not be directly linked to GO, since PEG has been previously linked to anaphylactic reactions (in sensitized individuals).^274−276^ Moreover, obstruction of lung capillaries by agglomerated GO might represent a critical factor in this study, and results cannot be generalized to all types of GO.

In an attempt to understand the role of surface chemistry, C57BL/6 mice were exposed to a single intratracheal instillation to 18, 54, or 162 μg of either 2–3 μm GO or 1–2 μm rGO.^277^ The impact on the lung and liver transcriptome was analyzed after 1 day to measure acute phase responses. While both materials triggered pathways related to ROS production and genotoxicity, GO induced wider perturbations across both lung and liver. GO also induced pathways related to fibrosis, as reported previously,^266^ despite the absence of fibrosis in lung sections, even at 90 days.^278^ This discrepancy was also observed after repeated exposures,^233^ implicating the presence of other factors required to trigger fibrosis. The 1-day transcriptomic results were in line with a previous report from the same authors using the same animal tissues,^278^ and suggested that higher surface oxygen content could be responsible for the greater reactivity of GO.

Despite the increasing number of *in vivo* studies dedicated to GBMs, there is surprisingly little progress on the pulmonary impact of other 2D materials. An oropharyngeal aspiration method was used to assess possible impact of different forms of MoS_2_ (aggregated with lateral size in the range of 0.54–1.1 μm; exfoliated by lithiation, with average size of 585–746 nm; dispersed using Pluronic F87 with average size of ∼80 nm) on the lungs of C54BL/6 mice at 2 mg/kg.^241^ Histopathological analyses at 40 h showed focal areas of inflammation for the aggregated MoS_2_, whereas the other two materials had little to no effect, despite all having been internalized in alveolar macrophages. In line with these results, aggregated MoS_2_ induced neutrophil recruitment via secretion of CXC chemokine, IL-6, and MCP-1 at 40 h. At 21-day post exposure, there was evidence of inflammation resolution for the aggregated MoS_2_ group, and none of the tested materials induced fibrosis despite the higher TGF-β content in bronchoalveolar lavage (BAL) fluids for both MoS_2_. In another study, mice were intratracheally exposed to MoS_2_ nanosheets (with lateral sizes in the range 50–150 nm) and sacrificed at 0.5–28 days after exposure to a bolus dose of 50 μg.^56^ BAL fluids showed increased levels of macrophages and neutrophils at 12 h, which reduced after 1 day. Cytokine profiles in BAL fluids were accordingly above control levels at 12 h, but were below control levels at day 1 and 2, reaching baseline thereafter, suggesting rapidly resolved acute inflammation despite persistence of various biotransformed molybdenum-based structures (from sheets to rolls, see “Biodistribution of 2D Materials”) in alveolar macrophages up to one month. Interestingly, the authors also found such structures in extracellular vesicles in BAL fluid, suggesting a mechanism of inflammation resolution by eliminating the offending material. The pulmonary toxicity of MoS_2_ sheets (with size of 97 nm and 1.9 μm) was also investigated in Sprague–Dawley rats at 1 and 7 days after single intratracheal instillation at 1.5 and 5 mg/kg.^279^ BAL fluids demonstrated high level of material internalization in alveolar macrophages, and slightly higher number of neutrophils compared to control at 1 day (resolving by day 7). While cytokines and protein content were not statistically increased in comparison to the vehicle control at day 7, there was a higher lactate dehydrogenase (LDH) content in MoS_2_-treated groups after 1 day, albeit not statistically significant. Blood biochemical parameters were not altered at either at 1 or 7 days after exposure to any MoS_2_. DNA damage assays using peripheral blood lymphocytes also revealed no effect for the two materials. However, on day 7, dose-dependent histopathological signs of inflammation were noted for both materials, more severe for the micron-sized MoS_2_ compared to the nanosized MoS_2_. In addition to accumulation in macrophages, MoS_2_ sheets were also identified in epithelial cells, suggesting that these materials could eventually translocate through the air–blood barrier. Regardless, the authors concluded that MoS_2_ had little to no pulmonary impact.

### Focus on *In Vitro* Effects

Numerous cell culture (*in vitro*) studies of 2D materials have been published in recent years. Cell culture models based on submerged exposure (material suspension dispersed in cell culture medium) are most often used to test nanomaterial cytotoxicity. However, in recent years, “pseudo” air–liquid interface (ALI)^280^ and authentic ALI exposure models have emerged and have been applied for 2D material pulmonary toxicity assessment. A recent study showed that primary nontransformed normal human bronchial epithelial (NHBE) cells were far more sensitive to various GBMs than the well-established A549 lung carcinoma derived cell line.^281^ The three materials tested, namely GO (average lateral size: 1.18 μm), FLG (average lateral size: 300 nm), and smaller FLG sheets (lateral size: 36 nm) caused more than 90% cell death at concentration as low as 5 μg/mL, 7 days after exposure. In contrast, A549 cells displayed a toxic response to the three materials only at the highest dose (100 μg/mL) after 7 days and with about 50% of cell viability. The authors concluded that normal lung cells are a better cell model for GBM safety testing than lung cancer cells. However, it is not clear why the GBM responses were so severe in NHBE cells, although cell type could also make a difference, i.e., NHBE cells are bronchial epithelial cells and A549 cells are alveolar epithelial cells. Moreover, in the latter study, cells were maintained under submerged conditions, which may also limit inferences to the real-life *in vivo* situation. Interestingly, one study addressed these limitations and used a 3D mucociliary tissue model made of primary human bronchial epithelium (i.e., EpiAirway) exposed for 1 min every day for 30 days to GO (with lateral size in the range of 100–1500 nm) via a nebulizer system at 0.71 μg/cm^2^ dose (daily) reaching a cumulative dose of at 21 μg/cm^2^ after 30 days.^282^ GO sheets stimulated TNF-α and IL-1β secretion without oxidative stress after 2 weeks of continuous exposure, but there was no toxicity, compared to the bronchial epithelial cell line BEAS-2B (also maintained at ALI) in which significant toxicity was evidenced. This later result highlights the importance of functional mucociliary clearance, present in the EpiAirway^TM^ model and absent in BEAS-2B cells. The authors observed a GO-mediated inhibition of autophagy, which was alleviated when the accumulation of GO in cells decreased by exocytosis.

One of the events leading to pulmonary fibrosis is the occurrence of epithelial-mesenchymal transition (EMT).^283^ To assess the ability of rGO sheets (with lateral size in the range of 50–700 nm) to induce EMT, Zhu et al.^284^ exposed A549 cells under submerged conditions to increasing concentrations of material suspensions (1–20 μg/mL). While no significant impact could be identified on cell viability even at 20 μg/mL for 72 h, rGO promoted cell migration and invasion at low concentrations (1–10 μg/mL for 24 h) but inhibited it at higher concentration (20 μg/mL for 24 h). At the molecular level, these behavioral changes were reflected by a decrease in E-cadherin and Smad4 and an increase in Vimentin. Similar results were observed when A549 cells were exposed under submerged conditions to low concentration of GO materials (10 μg/mL for 24 h).^284^ Irrespective of lateral dimensions (large, 400–900 nm and small, 200–600 nm), GO sheets altered the expression of various biomarkers (decrease of E-cadherin, increase of Vimentin and N-cadherin or TGF-β receptor), activating the TGF-β-Smad2/3 signaling pathway that typically drives EMT. The authors further demonstrated *in vivo* that these GO sheets could promote tumor metastasis. Interestingly, the promotion of cell migration and invasion upon exposure to GO sheets (with average lateral size of 202 nm) was also confirmed in a different study^225^ in which the authors reported that GO exposure (10 μg/mL for 24 h) promoted release of HMGB1 by macrophages, leading to RAGE (receptor for advanced glycation end-products) activation and stimulation of the migration of A549 lung cells cocultured with GO pretreated macrophages.

Pro-inflammatory responses and DNA damage are considered key events in the progression to fibrosis and cancer, respectively. In an attempt to assess the ability of GO (size 2–3 μm) or rGO (size 1–2 μm) to trigger these two events, monocultures of A549 and THP-1 macrophages were exposed to materials under submerged conditions.^220^ Mortality of A549 or THP-1 remained below 20% even at the highest doses used (160 μg/mL for A549, 80 μg/mL for THP-1). There was a transient increase in IL-8 release for A549 at 6 h for both materials, returning to control values by 24 h. In contrast, there was no inflammation in differentiated THP-1 cells, but increased DNA damage at 24 h for GO at 10 and 40 μg/mL. These results were in agreement with previously published *in vivo* results from the same research group.^278^ The genotoxic potential of different types of FLG (∼500 nm nonfunctionalized, ∼430 nm amine-functionalized and ∼350 nm carboxylic acid-functionalized) was also tested at 24 h using the human bronchial epithelial cell line 16HBE14o^–^.^285^ The nonfunctionalized and amine-functionalized FLG were found to induce primary indirect genotoxicity via depletion of intracellular glutathione (GSH), decrease in oxygen consumption and adenosine triphosphate (ATP) production. Moreover, cytotoxicity was identified at 10 μg/mL and above for nonfunctionalized, at 50 μg/mL and above for amine-FLG and absent for carboxylic acid-FLG. All materials tested induced IL-8 secretion and depletion of GSH at nontoxic doses alluding to oxidative stress-mediated genotoxicity. Overall, carboxylic acid-FLG appeared to be the least toxic material. The same research group used similar materials to test genotoxicity in human transformed type-I (TT1) alveolar epithelial cell monocultures, differentiated THP-1 macrophage monocultures and their coculture under submerged conditions.^286^ All materials induced significant primary-indirect genotoxicity in monocultured TT1 cells, and secondary genotoxicity in the form of oxidative stress in the TT1/THP-1 coculture model. In a different study, the genotoxicity of a wide range of GBMs (13 types of GNPs and rGO of various sizes) was tested in submerged normal human broncho-epithelial BEAS-2B cells.^287^ For rGO, 3 out of 7 materials did not induce genotoxicity even at the highest dose (100 μg/cm^2^). In contrast, all 6 tested GNPs induced genotoxicity at 6 h. The extent of surface oxygen content leading to ROS production was identified as one of the main drivers of genotoxicity. An even wider range of 2D materials was studied in primary mouse tracheal epithelial cells (mTEC) and A549 cells.^288^ Graphene (average lateral size of 110 nm), GO (average lateral size of 400 nm or 2 μm), rGO (average lateral size of 400 nm or 2 μm), partially reduced GO (average lateral size of 400 nm), MoS_2_ (average lateral size of 400 nm), and hBN (average lateral size of 150 nm) were initially tested at high concentration (125–250 μg/mL). Only the two types of rGO and hBN caused significant mortality (10% for 400 rGO, 20% for 2 μm-rGO, and >40% for hBN). hBN was not toxic to mTEC at dose up to 80 μg/mL, but caused a dose-dependent toxicity to A549 with a 40% loss of viability at 40 μg/mL. hBN also impacted cell mobility in the latter cells, suggesting possible interference with the cytoskeleton. Two types of hBN sheets, obtained either with rhomboidal/cornered (“sharp”) or rounded edges were produced to study the potential lysosomal membrane damages in lung epithelial cell line (H460) induced by hBN.^289^ The rounded hBN (with an average later size of 156 nm) sheets accumulated in endolysosomes, while the “sharp” hBN (with an average later size of 342 nm) could also be found in the cytoplasm. Molecular dynamics simulations revealed that the hBN with “sharp” corners can penetrate the lipid bilayer and form a water channel across the membrane, while hBN sheets with rounded edges did not exhibit this behavior. Cathepsin B release in the cytoplasm was observed only with the sharp hBN, suggesting that lysosomal membranes were damaged. A higher level of superoxide was found after exposure to sharp hBN leading to apoptosis from concentrations as low as 20 μg/mL at 24 h. In contrast, rounded hBN caused limited amount of ROS production, which did not lead to loss of cell viability. In another study, THP-1 macrophages were exposed to hBN sheets (with average lateral size of 350 nm) for 24 h at concentrations up to 100 μg/mL.^290^ The cytotoxic effect was limited (below 10%) even at the highest concentration used. Despite oxidative properties, these hBN sheets did not activate the NFκB signaling pathway, and activated the NLRP3 inflammasome only at the highest dose. In line with these results, hBN caused limited inflammation-related protein release by macrophages. Finally, during bacterial challenge, hBN stimulated bacteria was taken up by THP-1 macrophages. Human primary alveolar epithelial cells were challenged with hBN sheets (in the range of 100–300 nm) at concentrations between 0.625 and 1280 μg/mL.^291^ The cytotoxicity at 72 h reached significant level at doses between 40 and 80 μg/mL, and was similar to positive control (e.g., H_2_O_2_) for 1280 μg/mL.

Concerning TMDs, the impact of WS_2_ (with size in the range of 50–200 nm) was studied in a variant of mouse Lewis lung carcinoma cells (LLC1).^292^ While toxicity was not observed at day 1 at any tested concentrations up to 25 μg/mL, by day 2 toxicity increased significantly reaching 30% for concentration as low as 0.25 μg/mL and almost 50% at 1 μg/mL. Moreover, aggregated, lithium-exfoliated and Pluronic F87-dispersed MoS_2_ were tested in THP-1 macrophages and BEAS-2B cells.^241^ None of the tested MoS_2_ induced cytotoxicity. However, aggregated MoS_2_ induced pro-inflammatory response in both types of cells, and a pro-fibrogenic response in the coculture model. These responses were ascribed to a higher deposited dose of the aggregated MoS_2_ compared to the other well-dispersed MoS_2_ materials.

Despite the many *in vitro* studies of 2D materials, it must be noted that the majority of studies are acute exposure studies (24–48 h), and acute exposure is not a good predictor of long-term effects, as shown in a recent transcriptomics study using BEAS-2B cells. Hence, both size-dependent and exposure-dependent differences were seen following short-term (48 h) versus long-term (28 days) exposure to GO of varying lateral dimensions (lateral sizes of 1–30 μm for large GO, 50–2000 nm for small GO, and 50–300 nm for ultrasmall GO), with evidence of mitochondrial dysfunction at 48 h and subversion of apoptosis pathways at 28 days.^293^ Indeed, in terms of knowledge gaps, there is no long-term study to assess the carcinogenic potential of 2D materials. This is necessary for the regulation of 2D materials.

Few studies have addressed interactions of GBMs using advanced *in vitro* models including models of human airway or organoids, that are more representative of *in vivo* physiology. These models are of higher relevance than standard cell culture models, due to more accurate recapitulation of the interplay between different cell types and 3D architecture that better corresponds to human tissue. Human airway model containing ciliated and mucus-producing goblet cells were exposed to aerosolized GO for the period of 30 days. Interestingly, the slow uptake of GO started to occur only after 15 days of exposure, predominantly by endocytosis.^282^ GO flakes were localized in endosomal vesicles, indicating subcellular trafficking of the material toward the lysosomes. However, most of the material was trapped in the mucus and did not reach the cells. This is in agreement with the results recently obtained after exposing human lung organoids (HLO) derived from embryonic stem cells to GO with different lateral dimensions.^294^ The HLO contained six major epithelial cell types found in the lungs and were functional in terms of expressing beating cilia and secreting mucus and surfactants. The vast majority of GO sheets (regardless of their size) remained trapped in the mucus/surfactant and there was no or very limited uptake of the materials by the cells 7 days after microinjection of the materials directly inside the lumen of the organoids. Despite progress, there remain numerous knowledge gaps not only for GBMs but also for hBN or TMDs. Therefore, drawing valid conclusions for materials within a category (for instance, all types of GO) will require more experimental work. Moreover, high-throughput screening tools have improved in the past decade,^295^ and are expected to reach the maturity and throughput necessary to speed up pulmonary toxicity testing of 2D materials. The use of machine learning is yet another emerging approach to analyze and interpret the results of multiple toxicity studies, thus supporting predictive 2D nanotoxicology.^296^

## Impact on Liver, Spleen, and Kidneys

### Focus on *In Vivo* Effects

Upon reaching the deep lung and alveolar space, 2D materials may translocate to the blood circulation from the airways and reach secondary organs, in particular the liver and spleen. For instance, GO sheets of different lateral dimensions were found to translocate and accumulate to a small but significant extent in spleen after intranasal administration.^266^ In order to better understand how this accumulation could impact this important organ,^297^ single-to-few layers GO (lateral size between 80 and 100 nm) were directly injected in the tail vein of C57BL/6j mice.^298^ Several studies have applied this route of administration as proxy to model air–blood barrier translocation and subsequent accumulation in the spleen.^39,299,300^ Despite the presence of GO in this organ, in particular in marginal zone macrophages, there was no acute (1 day) or long-term (28 days) damage to the histological macro-architecture of the organ after intravenous injection at any tested dose (from 2.5 to 10 mg/kg).^298^ In addition, the hematological and immunological functions of the spleen were tested. Even at the highest dose, the spleen could function normally and eliminate aged or aberrant red blood cells. There was, however, a significant albeit very limited change in the number of T cells (CD4^+^ and CD8^+^) in comparison to control. Concerning the pro-inflammatory mediators, IL-6, IL-1β, and TNF-α, and anti-inflammatory mediators, IL-10 and TGF-β, there was only increased expression of IL-1β at 1 day for all doses tested, resolving by day 28 for the lowest doses, but leading to a significant decrease in IL-1β expression compared to control for 10 mg/kg.^298^ Similar lack of histological damage to the spleen or liver, another organ where injected particles tend to accumulate, was reported in a study using GO functionalized for tracking purposes.^298^

Focusing on liver toxicity after 2 or 5 mg/kg intraperitoneal injection of GO (in the range of 1–1.5 μm) for five consecutive days in mice, it was reported that the water content, malondialdehyde and peroxidase levels were dose-dependently increased.^300^ In agreement with the increased water level and induction of oxidative stress, liver histopathological analysis demonstrated hepatocyte swelling, hence displaying all hallmarks of fatty liver (i.e., hepatoxicity) in the 5 mg/kg group. In addition, there was a decrease of aspartate transferase/alanine transferase (AST/ALT) ratio in the 2 mg/kg GO group and an increase in AST/ALT ratio in the 5 mg/kg GO group compared to the control group, further suggesting that GO irrespective of dose was altering liver function. In the same study, it was also shown that intravenously administrated GO could be eliminated via the urinary system, as previously shown by other investigators.^39,301,302^ The authors decided to assess the plasma level of creatinine as biomarkers of kidney function and found it to be decreased in GO-exposed groups compared to the control group, thereby confirming kidney damage.^300^ Using a similar route of administration, larger dimension GO (5–10 μm) was administered 15 times over a period of 30 days to Wistar rats at 0.4, 2, or 10 mg/kg.^303^ Liver and kidney histopathology and blood biochemical analyses were performed and dose-dependent toxicity was observed. The mid- and high-dose induced histopathological damages, more pronounced in the liver at high-dose. In addition, biomarkers of hepatic function (e.g., ALT, alkaline phosphatase ALP, AST) were altered in the highest dose group, while there were no changes in the two lowest dose groups. The detoxifying enzyme catalase was decreased whereas levels of the oxidative stress marker malondialdehyde were increased in tissue homogenates. Both GO dose and lateral dimension affected renal excretion pathways and kinetics as well as extent of kidney injuries in CD1 mice.^304^ The small GO materials (162 nm) were eliminated via glomerular filtration and induced structural alterations in glomerular podocytes while larger GO materials (330 nm) were eliminated faster via the proximal tubules. At 15 mg/kg and both 2 and 7 days, both materials injured renal tubular epithelial cells, causing loss of brush border, cast formation and tubular dilatation, as well as increased glomerular diameter. In agreement with these injuries, albumin to creatinine ratio was increased in small GO at the early time point and increased in large GO group at the later time point. GOs varying in lateral dimensions (e.g., 50–200 nm, 200–500 nm, and 500–2000 nm) were used in another study in which it was found that GO accumulated in the liver and lungs, but not in the spleen of intravenously injected mice at 5 mg/kg.^305^ Furthermore, hepatic IL-6 levels were increased with the dose and lateral dimensions in IL-6 reporter mice, peaking at 9 h after injection and decreasing thereafter to reach baseline level at 48 h. This hepatic inflammation was associated with induction of ROS production via activation of the NFκB signaling pathway in the liver. GO sheets accumulated primarily in Kupffer cells, and to a lower extent in hepatocytes. The authors demonstrated that hepatocyte IL-6 secretion was linked to the release of pro-inflammatory mediators such as IL-1β and TNFα by Kupffer cells and M1 macrophage polarization upon TLR4 activation by the GO materials. The impact of liver accumulation was also investigated after intravenous injection of very small GO (in the range of 10–20 nm) in mice at 2 mg/kg daily for 7 days.^306^ Interestingly, a zonation pattern specific to this GO was revealed, akin to the zonation pattern reported in the spleen for other GO materials.^298^ GO accumulated preferentially in the portal triad rather than the vicinity of the central vein in hepatic lobules.^306^ Despite minimal changes in the liver function studies, RNA sequencing and DNA methylation sequencing analyses revealed that this location-specific accumulation led to a location-specific alteration of the transcriptome and epigenome in GO injected mice, with hepatocytes in the portal triad zones displaying greater functional and phenotypic disorders. This study shows that detailed investigations are needed to understand the impact of GO and other 2D nanomaterials on the liver.

**Figure 15:**
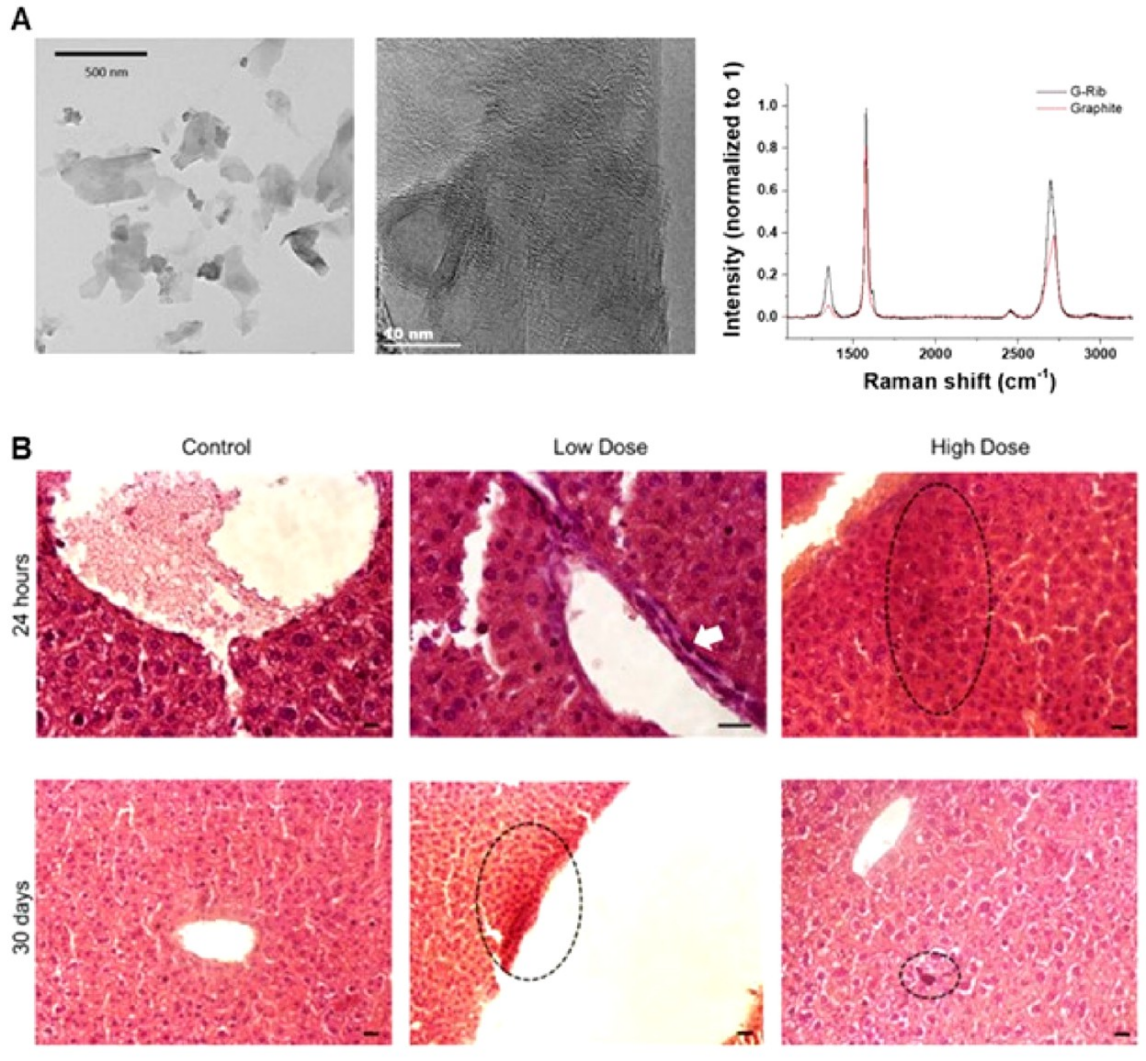
Graphene dispersed using biocompatible riboflavin causes no liver damage in mice upon intravenous administration. (A) Transmission electron microscopy (TEM), high-resolution (HR)-TEM, and Raman spectroscopy of graphene-riboflavin sheets. (B) Hematoxylin and eosin (H&E) staining of liver sections from control or graphene-riboflavin-exposed mice (low dose: 5 mg/kg body weight and high dose: 15 mg/kg body weight) at different times postadministration. Dotted circles indicate possible nanomaterial accumulation in the tissue. The white arrow indicates the recruitment of Kupffer cells in the liver 24 h after injection. Scale bars: 20 μm.^39^ Reproduced with permission from Ruiz, A.; Lucherelli, M. A.; Murera, D.; Lamon, D.; Menard-Moyon, C.; Bianco, A. Toxicological Evaluation of Highly Water Dispersible Few-Layer Graphene in vivo. *Carbon* 2020, *170*, 347–360. Copyright 2020, Elsevier.

Surfactants involved in the dispersion of 2D materials can contribute to biological effects. In a recent study, GO or Pluronic 103-stabilized GO sheets of 250 nm average lateral dimensions were intravenously injected in rats (0.5 mg/kg).^307^ Histopathological, hematological and biochemical analyses showed no sign of any effect for Pluronic-stabilized GO. In contrast, GO induced inflammation in the liver and spleen, as well as alterations of lung and kidney structure. Biochemical markers were also altered in the GO group compared to control or Pluronic-stabilized GO group, suggesting that dispersion in Pluronic 103 was mitigating the toxicity observed without this surfactant. Riboflavin-dispersed FLG (average size: 840 nm) was intravenously injected in BALB/c mice at 5 and 15 mg/kg to assess distribution and toxicity.^39^ Histological analysis showed that despite hepatic accumulation and translocation into the bladder (i.e., urine) through the glomerular filtration barrier, FLG did not damage anatomical structures of the liver or kidneys (Figure 15). In agreement with these results, blood biomarkers of hepatic function (e.g., AST, ALT, and ALP levels) and renal function (e.g., urea and creatinine levels) were not altered. Most hematological markers were also in the normal ranges, although platelet count was significantly higher in the 5 mg/kg group. Finally, immune cells isolated from the spleen and lymph nodes of FLG exposed animals showed limited signs of alteration in the FLG exposed group compared to the control group. Another study reported that smaller FLG (in the range of 330–630 nm) was able to induce damages to red blood cell membranes, accumulation of free iron in hepatocytes, and enhanced erythro-phagocytosis by Kupffer cells, upon its accumulation in the liver after intravenous injection in mice.^308^ These effects were not found when even smaller material (in the range of 20–40 nm) was injected. These alterations were not hazardous to animal health. They led instead to the biodegradation of the larger FLG via Fenton reaction and hydroxyl radicals within one year.

Apart from GBMs, other 2D materials such as hBN and TMDs have been investigated in the liver, pancreas, and spleen. In another study, the acute (24 h) impact of hBN (in the range of 50–200 nm) after intravenous administration at 0.5–3.2 mg/kg was investigated in Wistar rats.^309^ Effects were identified at the two highest doses on thiol–disulfide homeostasis in serum (measuring total thiol, native thiol, and disulfide concentrations). Further investigating the thiol–disulfide homeostasis in different organs, (e.g., liver, spleen, kidney, heart, and pancreas), changes to homeostasis were found in the heart and spleen at 0.8 mg/kg but not at higher doses. In addition, the authors reported an increase in the level of lipid hydroperoxides (proxy for oxidative stress) and myeloperoxidase (proxy for neutrophil infiltration and inflammation) and a decrease in catalase levels (proxy for antioxidant defense) at the two highest doses in the same organs.^309^ The main limitation of the above study is that a single time point was analyzed, leaving open the question of long-term impact of such alterations or possible recovery/resolution. In another comprehensive study, the fate of MoS_2_ nanodots (3.3 nm) coated with human serum albumin (HSA) was monitored following intravenous administration in BALB/c mice at 5 mg/kg.^310^ Upon injection, a protein corona was formed on the MoS_2_-HSA complexes (increasing the size of the complexes to 24.5 nm). Interestingly at 30 min after injection, 75% of the blood molybdenum content was located with platelets, likely because fibrinogen was highly abundant in the protein corona of the MoS_2_ complexes. The biodistribution profile demonstrated that most MoS_2_ complexes were trapped very quickly and up to 60 days in the liver and spleen. In the liver, the complexes were located in the Kupffer cells, while in the spleen they were located within macrophages of the red pulp. Moreover, it was demonstrated that apoliprotein E (ApoE) was a key factor for this accumulation in the resident macrophages of the liver and spleen. Interestingly, the authors demonstrated that molybdenum translocated after day 3 from spleen to liver where it was incorporated into molybdenum-dependent enzymes and boosted their catalytic activity. However, despite acute hepatic inflammation, the MoS_2_ nanodots had no long-term toxic effects on liver or spleen.^310^

### Focus on *In Vitro* Studies

Understanding the impact of 2D materials specifically on hepatic stellate cells that initiate liver fibrosis is important. To this end, human hepatic stellate LX-2 cells were exposed to GO sheets (in the size range of 182–836 nm).^311^ A decrease in cell viability and mobility (cessation of cell movement due to disturbance of actin cytoskeleton) was found at 100 μg/mL, in agreement with a disruption of the cell mitochondrial membrane and membrane potential, which were associated with an induction of oxidative stress via ROS production. GO also stimulated protein expression of αSMA, a biomarker of fibrosis via modulation of the TGFβ pathway. In an alternative cell model to primary human hepatocytes, GO (with average size of 360 nm) at 80 μg/mL for 24 h was found to activate early apoptosis but not oxidative stress or inflammation.^312^ In addition, this GO material impaired cytochrome P450 phase-I drug metabolism enzymes, but it had no effect on phase-II enzyme GST or phase-III efflux transporter ABCG2. Phase-I drug metabolism enzyme alteration was associated with the alteration of gene expression and protein levels for several acute-phase proteins. Overall, this study showed that GO had an impact on the hepatic acute phase detoxification response. In a L02 liver cell model, it was demonstrated that GO nanosheets around 15 nm could absorb microcystin-LR (MC-LR), a liver toxin produced by cyanobacteria commonly found in water.^313^ Compared to free MC-LR and free GO, GO-bound MC-LR induced more apoptosis and ferroptosis in HaCaT (skin) and L02 (liver) cells. The concomitant induction of apoptosis and ferroptosis is unexpected as ferroptosis is defined as a nonapoptotic (caspase-independent) cell death. Nevertheless, GO and GO-MC-LR complexes were found to trigger oxidative stress, production of mitochondrial ROS, and iron accumulation, leading to mitochondrial dysfunction and cytoskeletal damage.

Other 2D materials such as hBN and MoS_2_ have also been investigated. The toxicity of hBN (with average size of 86 nm) and MoS_2_ (with average size of 56 nm) dispersed in Pluronic F87 was compared to their aggregated forms in different liver cells, (e.g., Kupffer cell-like KUP5, SV40-transformed murine LSECs, and Hepa 1–6 cells).^314^ Both aggregated and Pluronic-dispersed MoS_2_ induced cytotoxicity in Kupffer cells but not in the other cell types in a dose-dependent fashion, whereas hBN was not cytotoxic in any form. Importantly, the authors revealed that the adverse effects of dispersed and aggregated MoS_2_ were due to dissolution and release of molybdenum ions, which induced mitochondrial ROS production and apoptosis. Moreover, the increased uptake of aggregated MoS_2_ in Kupffer cells led to pyroptosis with NLRP3 inflammasome activation.

## Impact on the Cardiovascular System

The involvement of airborne particulate matter (≤2.5 μm in diameter) (PM_2.5_) in the development of cardiovascular disease is well established.^315^ Toxicity pathways are complex and involve inflammation, oxidative stress, and atherosclerosis, implicating autonomic nervous system reflexes via the respiratory and central nervous system.^316^ Similarly, engineered nanomaterials may exert detrimental cardiovascular effects.^317^ Indeed, the passage of inhaled gold nanoparticles from the lungs into the blood with accumulation at sites of vascular inflammation has been evidenced in human volunteers.^318^ The latter study thus provides a potential explanation for the link between environmental exposure to particles and cardiovascular disease. However, less is known about the cardiovascular impact of graphene and other 2D materials.^3^ On the other hand, several applications based on 2D materials are currently under consideration including biosensors,^319^ heart valves,^320^ and other cardiac devices.^321^ It is therefore prudent to ensure cardiovascular safety of these 2D materials.

In a recent study, mice were exposed via oropharyngeal aspiration to three different sizes of GNPs (lateral dimensions of 1, 5, or 20 μm, and thickness of 1–2, 7, and 7 nm, respectively). Additionally, GO and rGO were also investigated.^322^ The authors found that GO was more toxic compared to rGO. GO altered gene expression in the heart and kidney, in particular factors associated with inflammation (i.e., *MT1a*, *CCL24*, *Cxcl14*), cell signaling (i.e., *Creb*) and remodeling factors (i.e., *Col1a1*, *Fn1*). rGO resulted, however, in increased sensitivity to phenylephrine-induced vasoconstriction and elevated cardiac concentrations of H_2_O_2_.^322^ Overall, exposure to graphene nanoparticles produced physiological and alterations that could potentially lead to cardiovascular dysfunction. In another study, rats were exposed to GO (lateral dimension: 5–10 μm, and thickness: 0.8–2 nm) at dose rate of 50, 150, or 500 mg/kg via intraperitoneal injection every 48 h for 1 week. The authors found no effect on the heart in contrast to the liver, spleen, and lungs.^323^ Other investigators explored the *in vitro* impact of graphene nanoparticles (GO and rGO) using the rat myocardial cell line H9c2.^324^ The authors found GO and rGO reduced the viability of cardiac cells with IC_50_ values of 652.1 ± 1.2 and 129.4 ± 1.2 μg/mL, respectively. Hence, rGO particles produced a 5-fold increase in cytotoxicity when compared to GO, suggesting that the surface chemistry of graphene including the amount of oxygen functionalities, may play a role. The possible cardiovascular impact of other 2D materials, e.g., MoS_2_ and hBN, remains to be understood.

## Reproductive and Developmental Impact

The possible impact of 2D material exposure on reproductive health and offspring development remains an important subject of investigation.^3^ Early work showed that rGO, tested at 1–25 μg/mL, did not affect viability or initiate reactive species in human sperm unlike oxidized single-walled carbon nanotubes (SWCNTs).^325^ Moreover, GO has been postulated to increase fertilization potential due to its membrane cholesterol-extracting ability.^326^ No changes in epididymal sperm parameters, sperm production, or plasma testosterone levels were found in mice following pulmonary (intratracheal) exposure to GO.^327^ However, another study in rats found that intraperitoneal administered GO (10 mg/kg) for 15 and 30 days resulted in decreased epididymal sperm counts and elevated sperm abnormalities, with increased testes superoxide dismutase (SOD), gluthathione peroxidase (GPx), and malondialdehyde. Recovery was noted after 30 days of withdrawal and no effect on male fertility was observed.^328^

Maternal exposure to nanomaterials can translate to fetal effects through effects on the placenta thereby impacting growth and development of the offspring. The placenta itself, a transient organ meant to support the growing fetus, evolves throughout pregnancy, increasing in surface area but thinning its barrier. The maternal side of the placenta is made up of syncytiotrophoblasts overlying cytotrophoblast cells, mesenchymal tissue, while inner endometrium constitutes the fetal side.^329^ It is increasingly clear that nanoparticles are generally able to accumulate and eventually cross the placenta based on numerous *in vitro* coculture and *in vivo* animal and human models, as extensively reviewed,^330^ requiring further research using models that can effectively elucidate potential toxicity mechanisms. *Ex vivo* placental perfusion with an actual human placenta perfused with tested material is currently being applied as a method to investigate placental translocation.^331^ Additionally, 3D coculture models comprising placental fibroblasts surrounded by trophoblasts^332^ and coculture models comprising trophoblast (BeWo cells) and placental microvascular endothelial cells (HPEC-A2)^333^ and placenta or placenta-embryo chip models^334,335^ are also emerging as advanced tools for nanosafety assessment in pregnancy. Using the human trophoblast cell line BeWo, Kucki et al.^336^ found no evidence of pronounced cytotoxicity for four different commercial or research-grade GO materials after 48 h of exposure. However, GO induced a transient opening of the trophoblast barrier as evidenced by a temporary increase in the translocation of sodium fluorescein. Cellular uptake of GO (including large GO flakes of 10–30 μm) was observed by transmission electron microscopy, Hence, even though GO did not elicit major adverse effects on BeWo trophoblast cells, the pronounced cellular internalization as well as the potential adverse effects on hormone release and barrier integrity warrants further studies on the long-term consequences.

Recent work has shown that MoS_2_ conjugated with the antioxidant catechin did not negatively affect swine sperm capacitation.^337^ Others have explored the impact of MoS_2_ on chick embryos.^338^ The results revealed a high percentage of deaths and growth delays. Furthermore, immunohistochemical analysis showed a strong positivity for metallothionein in red blood cells in various tissues. Studies on developmental toxicity are currently lacking for hBN though the toxicity of boron is well-documented. However, one must remember that hBN is thermally and chemically stable and that most studies on boron are, in fact, focused on borate ions. More research is therefore needed to better understand the potential developmental toxicity of emerging 2D materials. It is notable, in this context, that developmental toxicity can occur in the absence of placental transfer of the toxicant.^339^

## Impact on the Gastrointestinal System

The human health impact of 2D materials on the gastrointestinal (GI) tract is an important area of investigation as ingestion represents a relevant exposure scenario for 2D materials in food or food packaging. Moreover, oral uptake is a secondary exposure route for inhaled particles that are cleared from the respiratory tract via the “mucociliary escalator” and subsequently swallowed. Early work on the impact of 2D materials (mostly GBMs) on the GI tract was based primarily on *in vitro* models of the GI epithelium or oral exposure in rodents.^340−342^ Collectively, these studies indicated no or mild acute toxicity of GBMs on the GI epithelium.^3^ In the past 5 years, further research was performed in the Graphene Flagship and beyond to close the remaining knowledge gaps on the impact of digestive fluids on 2D material biotransformation and toxicity, the genotoxic and inflammatory potential of 2D materials, and their interference with the microbiome. In addition to GBMs, studies are emerging on other 2D materials such as TMDs.

### From *In Vitro* to *In Vivo* Models

Previous work found no degradation of FLG and GO when using an *in vitro* digestion assay to simulate oral digestion, suggesting biopersistence when administered orally.^343^ Two recent studies revisited the physicochemical transformation of 2D materials (e.g., GBMs, hBN, and TMDs) in an *in vitro* simulated digestion system.^344,345^ In the initial study, size-sorted GO of submicron or micron lateral dimensions were examined with respect to physicochemical transformations across simulated digestions, and its toxicological assessment against an advanced *in vitro* cellular model of the human intestinal epithelium consisting of tricultures of Caco-2 enterocytes, HT-29 mucus-producing goblet-like column cells, and microfold (M) cells.^344^ The study showed that GO is reduced during simulated digestion and reacts with digestive enzymes. However, toxicological assessment of the GO small intestinal digesta over 24 h did not show any acute cytotoxicity. In the subsequent study by the same authors, a panel of 11 industrially relevant 2D materials, including graphene, GO, partially reduced GO (prGO), rGO, hBN, MoS_2_, and WS_2_, were evaluated by using simulated GI digestions and a triculture model of the human small intestinal epithelium.^345^ The 2D materials were dispersed in a fasting food model and subjected to 3-phase simulated digestion (representative of the oral cavity, gastric tract, and small intestine). This resulted in agglomeration of all the 2D materials, especially graphene, in the small intestinal digesta. In addition, MoS_2_ was dissolved by 75% by the end of simulated digestion. The 2D material small intestinal digesta over 24 h (1 and 5 μg/mL) did not induce acute toxicity in intestinal epithelial tricultures for most of the 2D materials with the exception of a low but statistically significant increase for the inorganic materials and GO dispersed in Pluronic F108.^345^ Taken together, these studies have confirmed that digestion of 2D materials does not enhance acute cytotoxicity toward the intestinal epithelium. However, further studies are warranted, especially for MoS_2_.

Domenech et al.^346^ investigated the genotoxicity of GO and GNPs *in vitro* using intestinal cocultures (Caco-2/HT-29). They showed that GO and GNPs (up to 50 μg/mL) induced DNA strand breaks after 24 h of exposure while cell viability, oxidative stress or barrier integrity were not affected. However, the genotoxic potential of GBMs needs further investigation to confirm the genotoxicity by other end points and to understand if the DNA damage persists or can be detected and repaired by the cells. Inflammatory responses of GBMs in the GI tract and adverse effects on the intestine in the state of inflammation were addressed in several recent studies. Lahiani et al.^347^ exposed *ex vivo* human colon tissue to graphene (1–1.2 nm thick, ≤10 μm lateral size), which resulted in an activation of genes involved in the binding, adhesion (e.g., *GTPase* and *KRAS*) and proliferation of epithelial cells (e.g., *PCNA*, *STAT3*) within 2 h as well as increased levels of pro-inflammatory cytokines IFN, IL-8, IL-17, IL-6, IL-9, MIP-1, and Eotaxin within 24 h.^347^ These results suggest that pristine graphene may activate the STAT3-IL-23-IL-17 inflammatory response in the gut. Two further studies assessed the inflammatory responses of GO on intestinal epithelial cells *in vitro* (e.g., NCM460 and FHC human colon epithelial cell lines) and in a mouse model of colitis *in vivo.*(348,349) In the initial study, Gao et al.^348^ could show that GO (up to 200 μg/mL; 24 h) induced dose- and time-dependent cytotoxicity in NCM460 cells and promoted inflammation, lysosomal dysfunction, and a block of autophagy. Furthermore, the treatment with GO (oral gavage; 60 mg/kg; every 2 days from day 2 to 8) in a dextran sodium salt-induced colitis mouse model resulted in an aggravation of the pathological condition, characterized by shortening of the colon, severe pathological changes, and induced autophagy. However, GO did not induce any adverse responses in healthy mice suggesting that simple *in vitro* epithelial GI monocultures lacking a mucus barrier may overestimate toxicity responses. Liu et al.^349^ explored the potential mechanism underlying GO aggravated colitis and inflammation in mice, revealing that GO triggered apoptosis in FHC cells through the activation of ROS/AMP-activated protein kinase (AMPK)/p53 pathway, as evidenced by the upregulation of cytochrome c, Bax, and cleaved caspase-3 and the downregulation of Bcl-2. In conclusion, these findings point toward an increased toxicity of GO in conditions of a pre-existing inflammation, which highlights the need to include diseased individuals in the safety assessment of 2D materials. The potential long-term GI effects from single and/or repeated 2D material exposure have not yet been extensively addressed. Recently, a study reported an oral exposure analysis delivering GO (30, 60, and 120 mg/kg) to the GI tract in mice every 3 days for 16 days by oral gavage.^350^ The authors observed dose-dependent ultrastructural intestinal alterations in colonic tissues including uneven arrangement and local atrophy of the microvilli, swelling of the mitochondria and endoplasmic reticulum, and widening of the intercellular spaces. No such pathological changes were observed in the studies discussed above,^348,349^ but it is unclear if these discrepancies are due to the slightly prolonged exposure or the use of different GO materials. Clearly, more work is warranted to understand long-term health effects of ingested 2D materials.

### Impact on the Gut Microbiome

**Figure 16:**
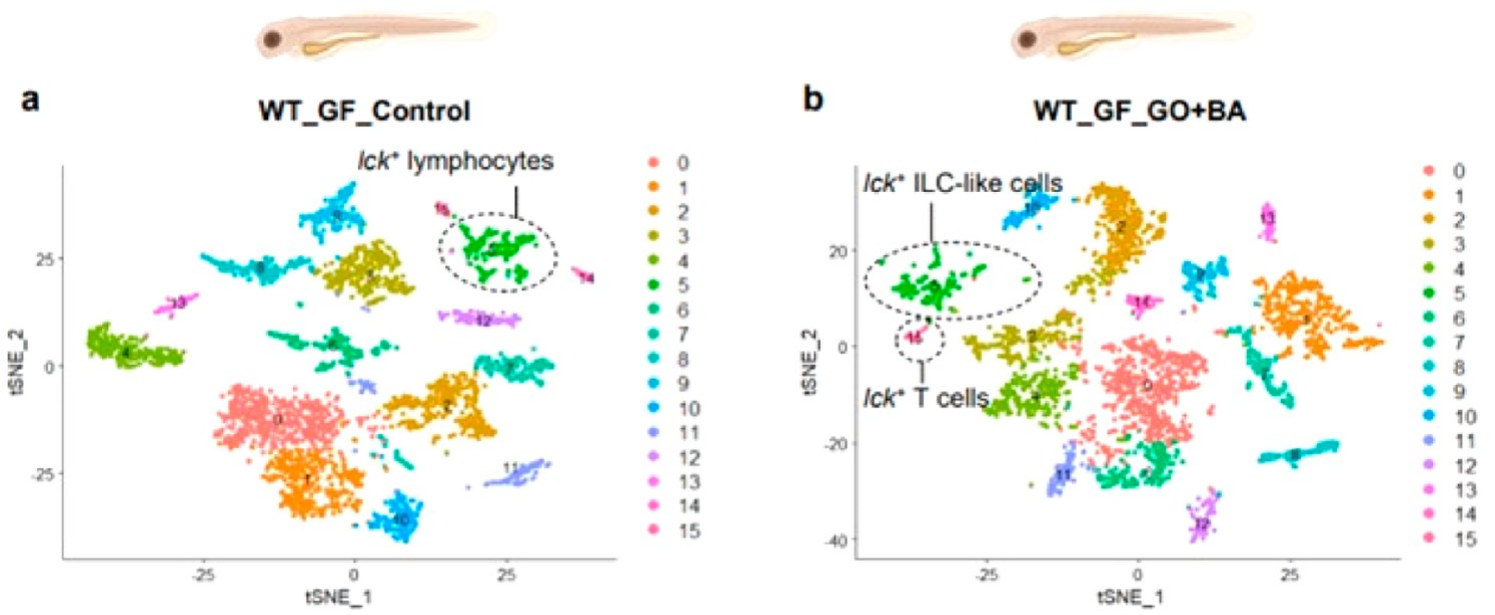
Graphene oxide elicits microbiome-dependent type 2 immune responses in zebrafish. Germ-free (GF) wild-type (WT) zebrafish embryos were unexposed or exposed to GO plus butyric acid (BA) (a short-chain fatty acid produced by bacteria in the gastrointestinal tract), and single-cell RNA sequencing was performed on whole zebrafish embryos. (a) The 2D projection of the t-distributed stochastic neighbor embedding (tSNE) analysis showing the *lck*^*+*^ lymphocytes (cluster 5) in control fish. (b) The 2D projection of the tSNE analysis showing the emergence of two separate *lck*^*+*^ clusters in fish exposed to GO+BA, i.e., *lck*^*+*^ (innate lymphoid cell) ILC-like cells (defined as *nitr*^*+*^*rag1*^*–*^) (cluster 5) and *lck*^*+*^ T cells (defined as *nitr*^*–*^*rag1*^*+*^) (cluster 15).^123^ Reproduced in part with permission under a Creative Commons BY 4.0 License from Peng, G.; Sinkko, H. M.; Alenius, H.; Lozano, N.; Kostarelos, K.; Brautigam, L.; Fadeel, B. Graphene Oxide Elicits Microbiome-Dependent Type 2 Immune Responses via the Aryl Hydrocarbon Receptor. *Nat. Nanotechnol.* 2023, *18*, 42–48. Copyright 2023, Nature Publishing Group.

The gut microbiome is sometimes considered as our “forgotten organ”. However, there is an emerging understanding that the microbiome in the GI tract is an important determinant of human health and disease.^351,352^ Therefore, it stands to reason that studies on the possible impact of 2D materials following exposure through the oral route should take the gut microbiome (and its metabolites) into account (Figure 16).^210^

Xie et al.^353^ reported on the impact of graphene (dispersed in deionized water with 0.9% NaCl and 0.1% Tween 80, followed by sonication for 30 min to improve material dispersibility) on the gut microbiome. To this end, mice were exposed to graphene for 4 weeks by gavage every day at the exposure dose of 1, 10, or 100 μg/day. The authors found that graphene exposure increased biodiversity of gut microbiota, and caused a shift in the microbial community. The 1 μg/day graphene exposure had a stronger influence on the gut microbiota than 10 and 100 μg/day exposures, which might be due to the aggregation of graphene at high concentrations. A comparative study was performed on SWCNTs, MWCNTs and GO with respect to inflammatory responses and intestinal permeability following oral exposure, including 16S rRNA sequencing to evaluate changes in the gut microbiome.^354^ For the latter study, mice were exposed at the dose of 2.5 mg/kg per day for 7 days. Overall, SWCNTs caused more severe changes to the GI tract. However, GO-exposed mice displayed an increased shift in the ratio of *Firmicutes*/*Bacteroidetes*, the two most abundant phyla in the mouse gut, when compared to the other tested materials. In a more recent study, GO was found to interfere with the composition of the gut microbiota during pregnancy which was associated with pregnancy complications.^355^ Mice were thus exposed at a dose of 2 mg/kg, 10 mg/kg, or 40 mg/kg by gavage daily during the entire organogenesis period (gestational day 7 to gestational day 16). Notably, in placenta tissues of pregnant mice exposed to GO at doses above 10 mg/kg, the expression levels of tight junction proteins (i.e., claudin1 and occludin) and vascular endothelial growth factor were markedly decreased, suggestive of an impaired placenta barrier. Mice exposed to 40 mg/kg showed an upregulated ratio of *Firmicutes/Bacteroidetes*, and the authors found that there was a strong link between a perturbed microbiome and abnormally expressed factors of the placenta barrier as well as adverse pregnancy outcomes.^355^ Interestingly, a recent study showed that carbon nanomaterials can influence gut microbiota in mice, undergoing degradation, transformation, and eventual fermentation into the interactive organic metabolite butyrate.^356^

Zebrafish are increasingly used as a model system in microbiome research.^357^ Zheng et al.^358^ exposed adult zebrafish for 21 days to graphene, GO, and rGO, and found that all three GBMs significantly altered the composition of the gut microbiota, while only graphene reduced the diversity or richness of the gut microbiota. Wu et al.^359^ exposed mice to 2D MoS_2_ sheets of varying lateral dimensions, i.e., nano-MoS_2_ and micro-MoS_2_, for 90 days. They found that nano-MoS_2_ caused more toxicity (intestinal inflammation) than micro-MoS_2_. Metabolome analyses showed that both types of MoS_2_ altered the metabolic profiles of the gut and the intestinal microbiota. In a recent study conducted in the Graphene Flagship, wild-type (WT) and aryl hydrocarbon receptor (AhR)-deficient zebrafish were continuously exposed for 7 days to a low dose (50 μg/L) or high dose (500 μg/L) of GO.^123^ AhR has emerged as an important environmental “sensor”,^360^ and the purpose of the study was to investigate the role of AhR for the impact of GO on the gut microbiome. In brief, GO (0.1–15 μm) was found to significantly modulate the gut microbiome, and these effects were shown to be AhR-dependent. Furthermore, using germ-free zebrafish, the authors could show that GO triggered a so-called type 2 immune response in zebrafish when combined with the short-chain fatty acid butyrate, a well-known microbial metabolite. Specifically, evidence for the upregulation of innate lymphoid cell (ILC)-like cells was obtained, and these effects were also shown to be AhR-dependent.^123^ GO thus appeared to act as a shuttle or delivery vehicle for butyrate (a known ligand of the AhR) leading to enhanced AhR activation in the gut epithelium, which in turn provided a signal for the homing and/or differentiation of ILC-like cells in the gut. This is the first study showing that a 2D material can influence the crosstalk between the microbiome and immune system via the AhR.

Overall, 2D material GI toxicity remains relatively unexplored compared to pulmonary toxicity. Digestion of 2D materials does not seem to cause acute gut toxicity in general, while graphene- and molybdenum-based materials have been implicated in dysbiosis. There is a dearth of long-term studies of 2D materials, as well as studies of vulnerable subjects.

## Impact on the Central Nervous System

The central nervous system (CNS) comprises of the spinal cord and the brain. There are three main types of neurons: (1) receptors, comprising the ganglia of spinal dorsal roots and cranial nerves with general sensory components; (2) effectors, comprising ventral horn cells, motor cranial nerve nuclei, and the autonomic nervous system; and (3) interneurons, which make up the majority of neurons in the CNS. Nanomaterials can cause neurotoxicity, neuroinflammation, and neurodegeneration by translocating across the blood–brain barrier, or via the olfactory route to the brain.^361,362^ The effects of GBMs on the CNS has been extensively covered.^3^ Here, we provide a snapshot of recent studies on the impact of GBMs on the CNS, addressing studies using *in vitro* and *in vivo* models as well as studies using alternatives to conventional animal models, such as roundworms and zebrafish.

**Figure 17:**
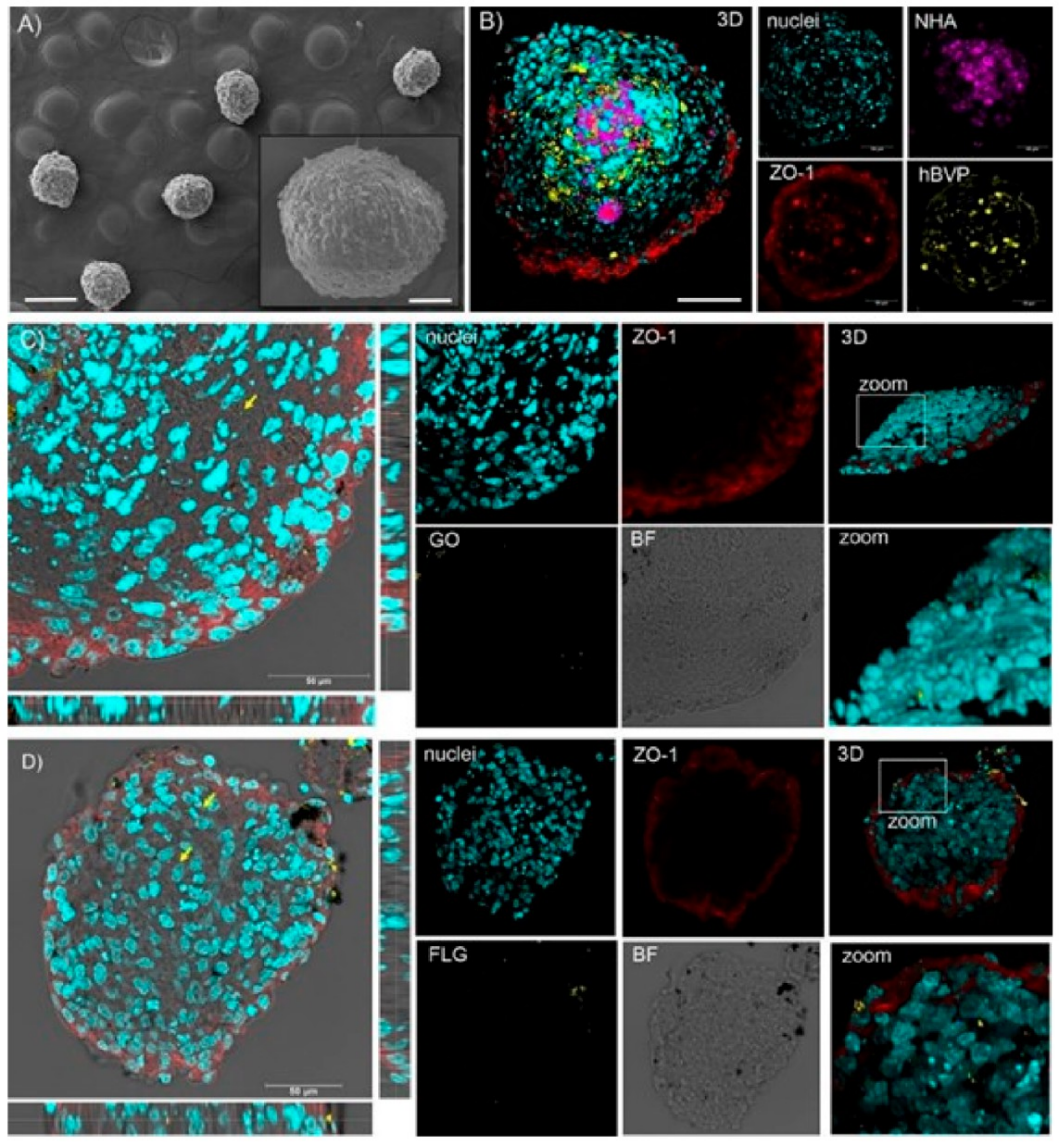
GBM interactions with a 3D model of the human blood–brain barrier (BBB). (A) SEM micrographs of the human multicellular assembloid model showing their spherical morphology. (B) Confocal imaging and 3D reconstruction of the assembloid model. Prestained primary human astrocytes and human pericytes are shown in purple and yellow, respectively; zonula occludens-1 (ZO-1) stained hCMEC/D3 (human brain endothelial cell) tight junctions are shown in red. Representative confocal XY planes, Z projections, and 3D reconstructions from a 20 μm slice of the multicellular assembloid model incubated with 10 μg/mL of GO (C) or FLG (D) for 24 h. Nuclei (Hoechst staining) are visualized in cyan, GO and FLG observed through light reflection mode are reported in yellow, and ZO-1 immunoreactivity is shown in red.^367^ Reproduced with permission from ref (367). Copyright 2023, the American Chemical Society.

2D materials are attractive candidates for treating neurological dysfunctions, exploiting a range of approaches, from photothermal effects to drug delivery.^363,364^ However, for any formulation intended to reach neurons of the CNS, the question of blood–brain barrier (BBB) penetration needs to be addressed. The majority of the biodistribution studies produced for different types of GBMs administered systemically point out a poor brain accumulation, suggesting low propensity to overcome the BBB.^39,365,366^ A recent study in the Graphene Flagship confirmed the low propensity of graphene materials to cross this barrier.^367^ The authors addressed the interactions of two GBMs (GO and FLG) with the BBB using *in vitro* models of increasing complexity (from 2D to 3D cell cultures) and observed uptake by endothelial cells. However, translocation was a rare event, and no adverse effects on the physiological properties of the BBB were observed (Figure 17).^367^ Besides graphene, several other 2D materials including hBN, BP, and TMDs are being considered for the treatment of brain tumors, to counteract amyloid aggregation, or for imaging.^368−370^ Needless to say, all of these applications require careful safety assessment of the 2D materials in question before the preclinical findings can be translated into the clinic. Close attention is also needed to the degradability of the materials (see section Biotransformation of 2D Materials); for some applications (e.g., drug delivery), degradation of the carrier may be desirable, while for other applications, e.g., regenerative or restorative medicine, degradation may be undesirable.

In a recent pilot study, MWCNTs and GO sheets (lateral dimensions between 10 and 1800 nm) were injected into the striatum of mice (the striatum is a nucleus, i.e., a cluster of neurons, in the subcortical basal ganglia of the forebrain).^371^ For comparison, cationic liposomes were also administered. For each nanomaterial, a total of 1 μL of a 0.5 mg/mL suspension in 5% dextrose was injected. While significant neuronal cell loss and sustained microglial cell activation were observed after injection of the liposomes, neither of the two nanomaterials triggered such effects, and GO appeared to elicit the least deleterious neuroinflammatory response. It is noted that the mice were only monitored up to 7 days postexposure. In a recent Graphene Flagship study, organotypic spinal cord cultures from mice were exploited to study the impact of “small” GO flakes (lateral dimensions about 100–400 nm) on astrocytes, key regulators of CNS homeostasis, and active players in neuroinflammation.^372^ GO protected the spinal tissue from dysfunctional signaling in response to a cocktail of pro-inflammatory cytokines. Furthermore, intravenous injection of GO ameliorated disease progression, reduced astrogliosis, and promoted neuronal survival in experimental autoimmune encephalomyelitis (EAE) mice, possibly through the modulation of Ca^2+^ signaling.^372^ Small GO (<500 nm) has also been used in proof-of-concept studies in applied neurology, showing benefit in limiting excitotoxicity in an *in vitro* Wistar rat model of ischemic stroke featuring oxygen-glucose deprivation. Glutamate-mediated excitotoxicity is affiliated with the pathogenesis of various brain maladies, ranging from ischemic stroke or brain injury to Parkinson’s and Alzheimer’s disease.^373^ The same GO was reported to downregulate presynaptic glutamate release in rats, highlightingu possible uses with regards to stress-related neurological diseases.^374^

Nonmammalian models have also proved themselves as valuable alternatives in evaluating neurological toxicity. The roundworm *Caenorhabditis elegans* and zebrafish (*Danio rerio*) have been used as models to explore the *in vivo* impact of GBMs targeting complex sensory-motor nervous system functions. At doses <50 mg/L, chronic exposure of *C. elegans* to graphite, GO, graphene quantum dots (GQDs) and nitrogen-doped GQDs induced impairment in body movements, arising from the damage of dopaminergic and glutamatergic neurons.^375−377^ In addition, *C. elegans* showed active avoidance of environmental GO (at concentrations >50 mg/L), a behavior supported by interneuron activity.^378^ Prolonged exposure to low doses of GO (1 mg/L) caused a decreased expression at interneuron synapses^111^ and an altered protein–protein interaction^141^ of the postsynaptic molecule Neuroligin 1. Several studies have shown that GBMs added to the zebrafish environment (i.e., water), accumulated in the brain of the zebrafish.^379,380^ In particular, GO (>0.1 μg/L) significantly disturbed locomotion, with the emergence of motor dysfunction, associated with dopamine decrease and brain histological features characteristic of Parkinson’s disease.^381^ In contrast, GO quantum dots (GO-QDs) (at 100 μg/mL) exerted protective effects in a zebrafish model of Parkinson’s disease, where they decreased neurotoxicity and counteracted swimming disruption.^379^ In another study, GO-QDs and rGO-QDs weakened locomotion and promoted thigmotaxis (i.e., the tendency to move toward physical contact with surfaces).^382^ However, tuning GO thermal reduction modulated the impact on the nervous functions, ranging from downregulation (GO) to upregulation (highly reduced GO) of zebrafish locomotor activity.^383^ In conclusion, these studies hint at the role of specific physicochemical features of GBMs in guiding their translocation and/or effects in the central nervous system. Further studies on other 2D materials using similar models are also warranted.

## Biodistribution of 2D Materials

In this section, we discuss organ accumulation and clearance (excretion) of GBMs and other 2D materials following different administration or exposure routes. The potential for biodegradation of 2D materials *in vitro* and *in vivo* will be discussed in the subsequent section.

### Graphene-Based Materials

The absorption, distribution, and excretion of 2D materials is influenced by physicochemical properties (i.e., lateral dimensions, thickness), surface functionalization, and route of exposure/administration. The formation of a so-called biocorona (see section Biotransformation of 2D Materials) may also influence the fate of 2D materials in a living organism. The pulmonary or inhalation route of exposure is of key relevance, not least in the occupational setting.^182^ Following inhalation, GBMs are mostly retained in the lungs.^384,385^ In a more recent study in the Graphene Flagship, FLG was found to accumulate long-term (e.g., 1 year) in Balb/C mice lungs, regardless of being administered at a high single-dose (e.g., 13 μg) or at a four-times weekly repeated lower dose (e.g., 3.4 μg).^386^ The effects of lateral dimensions of GO sheets were also compared after a single intranasal instillation in mice.^266^ To this end, ultrasmall GO sheets (<300 nm), small (50 nm–2 μm), and large GO sheets (1–30 μm) were synthesized. Using GO functionalized with DOTA (GO-DOTA) followed by chelation of GO-DOTA with metal isotopes (^111^In or ^115^In), the authors could show a size-dependent deposition in the lower respiratory tract. Moreover, large, micron-sized GO induced stronger pulmonary inflammation than the nanometer-sized GO, despite a reduced translocation to the lungs. RNA sequencing of lung tissues from exposed mice also revealed distinct size-dependent effects. However, although large GO triggered the formation of tissue granulomas, no pulmonary fibrosis was observed. MWCNTs, used as a positive control, triggered pulmonary inflammation similar or worse than large GO sheets. This behavior is strongly dependent on the shape. Hence, long and rigid MWCNTs have been described to behave like asbestos fibers, while graphene materials do not share the same behavior, underlining how shape is an important parameter that needs to be considered to avoid putting all carbon materials into the same category.^387^

**Figure 18:**
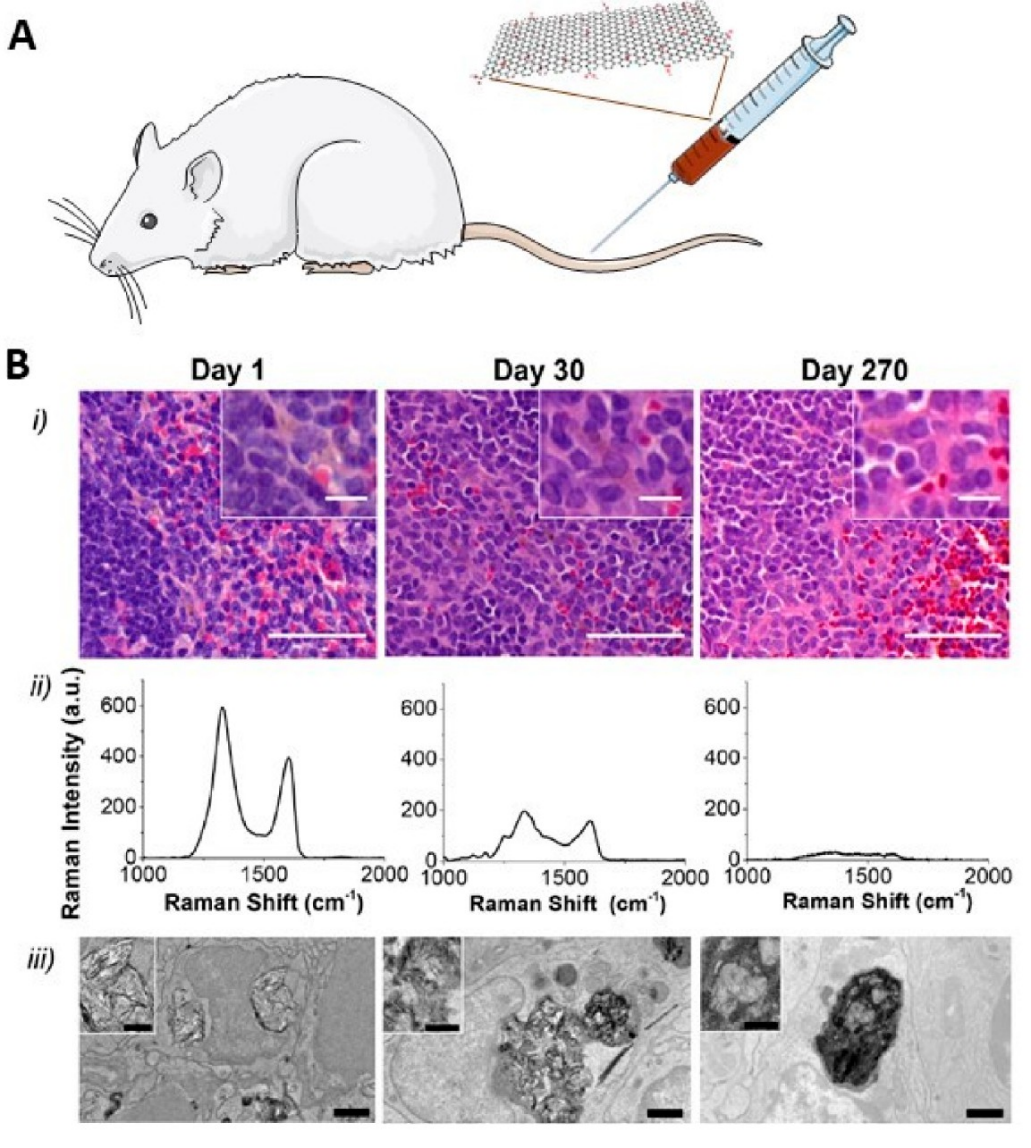
Evidence of splenic capture and intracellular biodegradation of graphene oxide in mice. (A) Schematic figure showing intravenous injection of GO in a C57BL/6 mouse. (B) Splenic biodegradation of GO over nine months (B) following i.v. administration at a dose of 7.5 mg/kg. (i) Splenic sections of mice that had been stained with hematoxylin and eosin (H&E); scale bars represent 50 μm. Inset images show the presence of GO material in the vicinity of cells of the marginal zone; scale bars represent 10 μm. (ii) Average Raman spectra of GO present in physically homogenized spleen tissue at different time points, *n* = 10 region of interest (ROI) × 3 mice. (iii) TEM micrographs of GO sequestered within the vesicular compartments of marginal zone splenocytes over time; scale bars represent 1 μm. The inset shows a magnification of the GO material at the respective time points; scale bars represent 500 nm.^298^ Reproduced with permission from ref (298). Copyright 2020, the American Chemical Society.

Intravenous administration is one of the common routes for biomedical applications of nanomaterials. Studies conducted in the Graphene Flagship have shown that following intravenous administration, organ biodistribution of GO showed bioaccumulation (after nine months) predominantly in the splenic marginal zone, with *in vivo* intracellular biodegradation of GO sheets also observed (Figure 18).^298^

**Figure 19:**
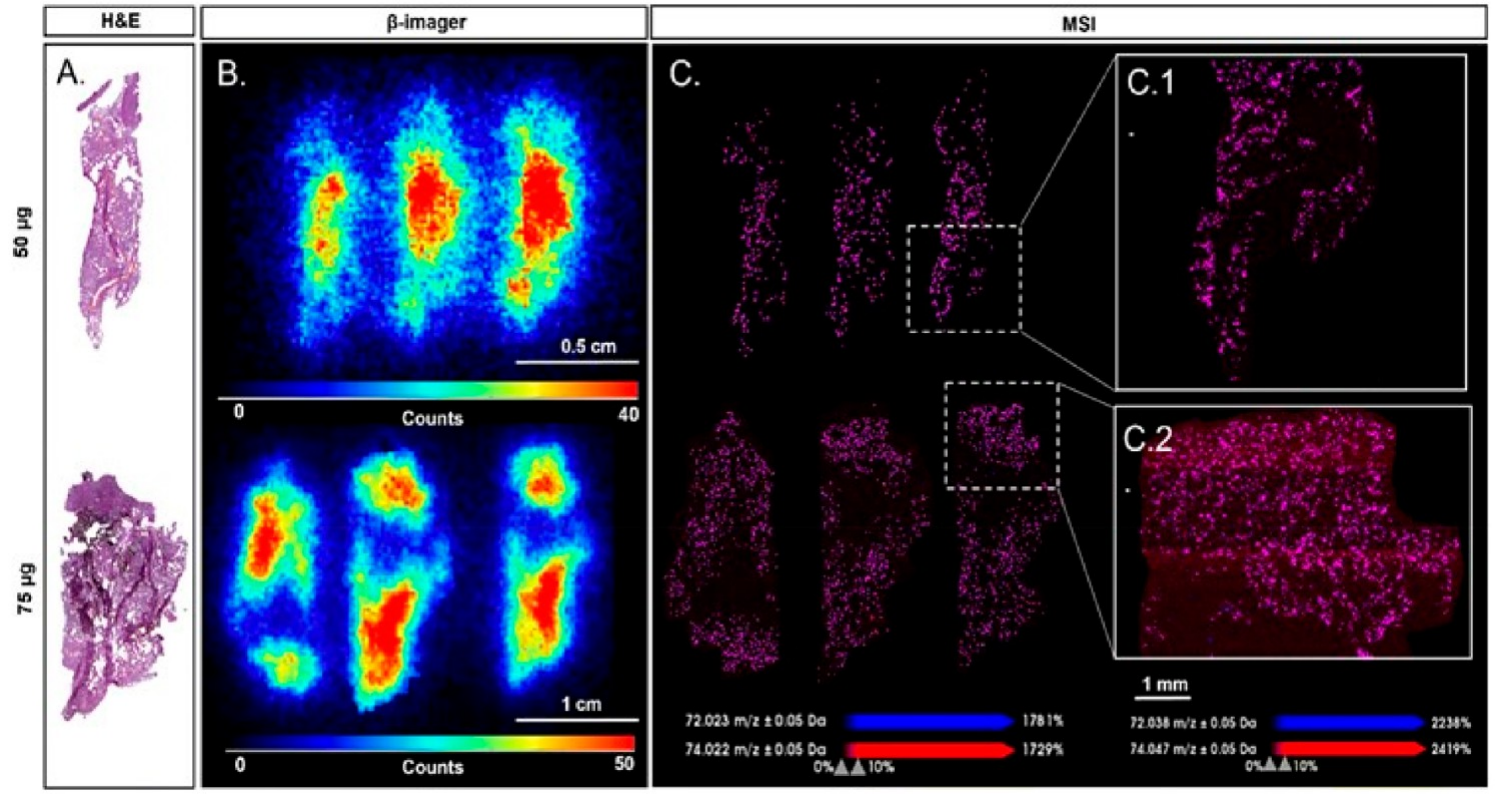
Biodistribution of ^14^C-graphene oxide following intravenous administration in mice. Comparison between hematoxylin and eosin (H&E), radioimaging and mass spectrometry imaging (MSI) of lung sections from mice exposed to 50 μg and 75 μg of ^14/12^C-GO. (A) H&E staining. (B) β-Imager acquisition of 50 μg and 75 μg injection dose with a spatial resolution of 150 μm. (C) MSI analysis of the same lung section from mice exposed to 50 μg and 75 μg of ^14/12^C-GO with a spatial resolution of 25 (inset C.1 and C.2) and 100 μm. Molecular images of GO were represented using the overlay of maps (purple) obtained for *m*/*z* 72 (blue) and 74 (red) ions.^391^ Reproduced in part with permission under a Creative Commons 3.0 Unported License from Cazier, H.; Malgorn, C.; Georgin, D.; Fresneau, N.; Beau, F.; Kostarelos, K.; Bussy, C.; Campidelli, S.; Pinault, M.; Mayne-L’Hermite, M.; Taran, F.; Junot, C.; Fenaille, F.; Sallustrau, A.; Colsch, B. Correlative Radioimaging and Mass Spectrometry Imaging: A Powerful Combination to Study ^14^C-Graphene Oxide *In Vivo* Biodistribution. *Nanoscale* 2023, *15*, 5510–5518. Copyright 2023, the Royal Society of Chemistry.

GO is a popular choice for biomedical applications due to its hydrophilicity and reported compatibility with biological systems. Large GO (1–35 μm) accumulated preferentially in the lungs compared to the spleen after intravascular administration, contrary to two smaller GO (small: 30 nm–2 μm and ultrasmall: 10–550 nm), which accumulated in the liver. Urinary excretion was not affected by lateral dimensions although its rate was influenced by the lateral size, with large GO excreting at slower rates compared to small GO and ultrasmall GO.^388^ In agreement with these findings, the distribution of ultrasmall GO sheets (10–20 nm) after intravenous injection (2.0 mg/kg) showed higher accumulation in liver and spleen compared to the lungs.^306^ Histological investigations indicated differential distribution of GO in liver lobules with a higher accumulation in the peripheral part (portal triad zone) compared to the center of the lobule (central vein zone). These findings are in agreement with previous studies.^323,389^ The distribution of FLG exfoliated with riboflavin (average lateral size of 840 nm) following i.v. administration at two different doses (5 and 15 mg/kg) has also been reported.^39^ In both cases, histological analysis showed that FLG sheets were present mainly in the liver up to 30 days, with no signs of hepatic toxicity. A previous study reported instead long-term hepatic toxicity of FLG (injected i.v. at 20 mg/kg).^365^ Differences in toxicity can be attributed to the method of exfoliation of graphene, which in the presence of riboflavin produces a more dispersible and stable nanomaterial reducing general toxicity. Large numbers of Kupffer cells were also able to capture FLG in the liver even after 3 months, alluding to degradation.^39^ Importantly, correlative radioimaging and mass spectrometry imaging (MSI) was recently applied to study biodistribution of ^14^C-GO (Figure 19).^390,391^ Following intravenous administration in mice, ^14^C-GO distribution was thus quantified by radioimaging on tissue slices, whereas on the same slices, MSI provided a highly resolved distribution map of the nanomaterial. Quantitative assessment showed greater amounts of GO in the liver than in the lungs, spleen, and kidneys. This approach could be advantageous in the preclinical development of GBM-based biomedical applications.

The ability of GBMs to cross the blood–brain barrier (BBB) remains a matter of debate. Syama et al. previously reported on PEGylated reduced “nano-graphene” (specifically, rGO) (lateral dimensions about 1 μm, thickness of 4–9 nm) in the brain of mice and suggested BBB crossing.^392^ Mendonça et al. reported that PEGylated rGO could induce disruption of the BBB leading potentially to brain entry.^393^ More recently, *in vivo* investigations confirmed BBB disruption in mice after acute exposure to rGO (10 mg/kg) encapsulated in micelles (100–200 nm). BBB crossing has been disputed by other investigators who suggested that labeling moieties could detach during circulation *in vivo*.^394^ GO was, however, found to translocate to the brain following intranasal instillation in a size-dependent manner, with trace amounts of ultrasmall GO in the brain up to 1 month postexposure.^298^

### Transition Metal Dichalcogenides and hBN

**Figure 20:**
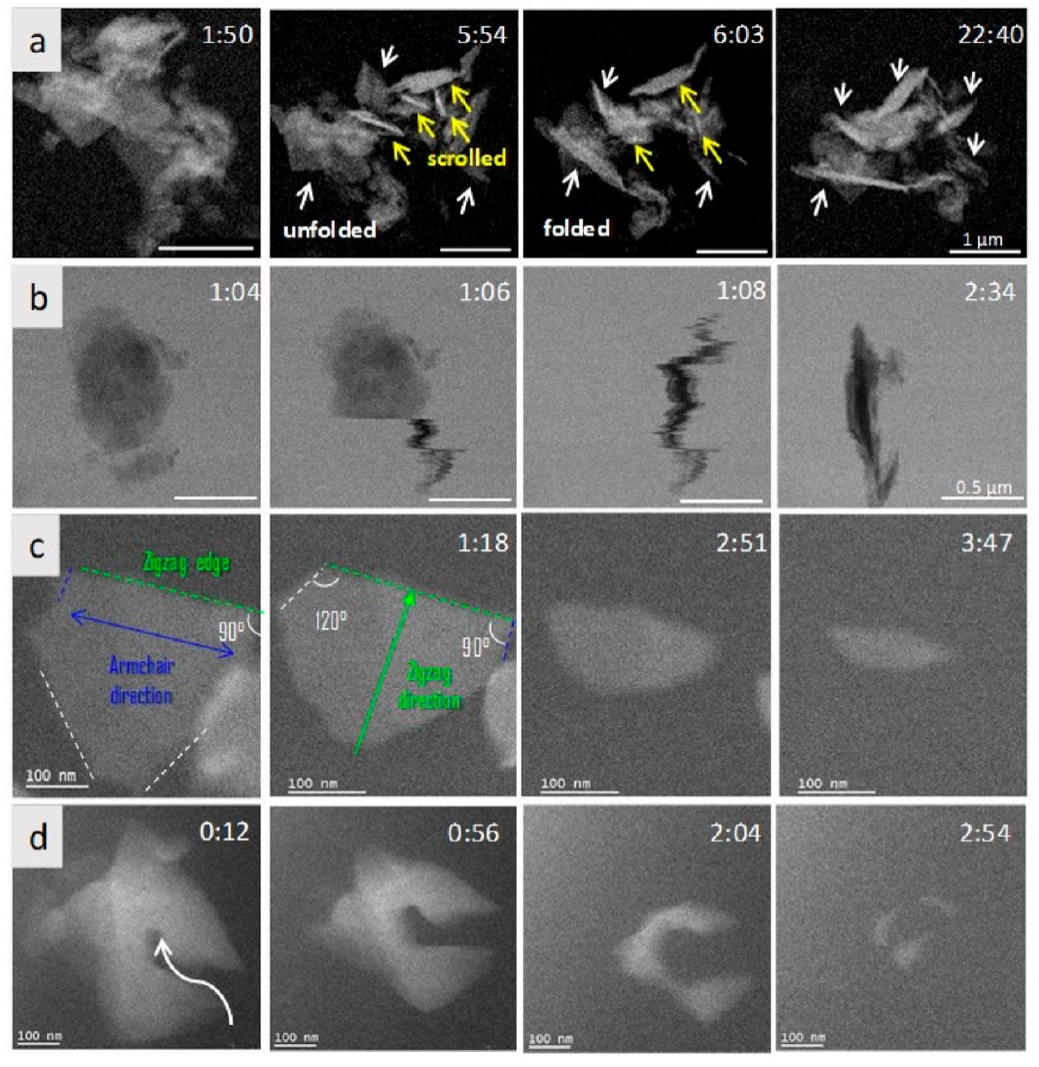
Evidence for dynamic nanoscrolling of MoS_2_ nanosheets. (a) STEM image sequence from *in situ* liquid phase recording of MoS_2_ sheets in 10 mm H_2_O_2_-DPBS. The white and yellow arrows point to sheets that fold and those that scrolled, respectively. (b) STEM image sequence from *in situ* liquid phase recording of free-standing MoS_2_ patch scrolling in 5 mm H_2_O_2_-DPBS. Time is indicated in min. The last two panels on the right side show the intermediate stages between a free-standing sheet and a fully scrolled needle. These are extracts of a movie showing MoS_2_ nanosheets forming dynamic nanoscrolls. (c) STEM sequence from *in situ* liquid etching of MoS_2_ sheets in DPBS-H_2_O_2_ solution. (d) Sequence from *in situ* liquid STEM displaying internal etching from edge defects of a single MoS_2_ sheet.^56^ Reproduced with permission from Ortiz Pena, N.; Cherukula, K.; Even, B.; Ji, D. K.; Razafindrakoto, S.; Peng, S.; Silva, A. K. A.; Menard-Moyon, C.; Hillaireau, H.; Bianco, A.; Fattal, E.; Alloyeau, D.; Gazeau, F., Resolution of MoS_2_ Nanosheets-Induced Pulmonary Inflammation Driven by Nanoscale Intracellular Transformation and Extracellular-Vesicle Shuttles. *Adv. Mater.* 2023, *35*, e2209615. Copyright 2023, Wiley-VCH Verlag GmbH & Co, KGaA, Weinheim.

The investigation of *in vivo* biodistribution, excretion, and toxicology profiles of TMDs is still limited. A very recent mouse study described the intramacrophage fate of 2D MoS_2_, which included nanosheet scrolling, oxidation and etching, as well as the release of molybdate ions (Figure 20).^56^

A previous study compared the *in vivo* behavior of PEGylated-MoS_2_, WS_2_, and TiS_2_ nanosheets of similar size.^395^ The nanomaterials (in the range of 100 nm size) showed predominant accumulation in the reticulum endothelial system (RES) such as liver and spleen after intravenous injection (10 mg/kg). In contrast with WS_2_ and TiS_2_, MoS_2_ showed biodegradation in the liver and almost complete excretion after one month through urine and feces possibly due to the oxidation of MoS_2_ into water-soluble molybdate species like MoO_4_.^2^ This is in agreement with the aforementioned mouse study in which *in vivo* degradation of MoS_2_ in the lungs was shown with partial excretion of nanoparticles by way of extracellular vesicles.^56^ With the aim of developing a drug delivery system, the distribution of MoS_2_ and PEG-MoS_2_ showed predominant accumulation of both nanomaterials in RES with a long retention time of more than 30 days for MoS_2_.^396^ PEG-MoS_2_ presented instead a faster excretion rate and was not observed in lungs, kidneys, heart, and brain. In another study, *in vivo* biodistribution of very small WS_2_ (with an average size of 37.5 nm) was monitored after intravascular injection in mice. WS_2_ nanosheets rapidly (within 1 h) accumulated in the liver, followed by distribution in the spleen, lung, and kidney, within 3 h. WS_2_ nanosheets were completely excreted after 3 days. Intraperitoneal injection was also investigated and showed similar distribution but with a longer residence time of WS_2_ for more than 10 days, mainly retained in the liver.^397^ It was evidenced that WS_2_ transformation is not complete and stops at the level of tungsten oxide, which is more biopersistent.^395^ It would, however, be preferable that the tungsten oxide eventually evolved into soluble nontoxic tungstate in an oxidative environment. To date, few *in vivo* studies of hBN have been performed (although some studies have addressed BN nanotubes).^398,399^ Despite earlier intravascular studies in mice showing radioisotope-labeled PEG-hBN (20 mg/kg) accumulating in the liver, lung, heart and spleen, with major toxic effects in the heart,^400^ boron nitride has been used in many topical products such as cosmetics and has been established to be nontoxic in amounts used even if inhaled.^401^ A rat study using an extremely high intravenous single dose of 1600 μg/kg hBN showed significant accumulation and damage at 24 h in the liver, kidney, heart, spleen and pancreas. However, 800 μg/kg showed no detrimental effect in terms of inflammation and cytotoxicity.^309^

## Biotransformation of 2D Materials

Many studies attempted to decipher cell uptake of GBMs, mainly taking into account the following parameters that drive 2D material interactions with biological systems: lateral dimensions and surface properties (e.g., oxygen containing groups and surface functionalization), cell types studied (phagocytic, nonphagocytic, cancerous or healthy cells) and material biotransformation (such as the presence of a surface-adsorbed biocorona and/or dissolution of the materials in various compartments in the body).^402,403^

Cellular uptake of GBMs has been extensively studied using professional phagocytes (macrophages) as well as other cell types such as lung and gastrointestinal epithelial cells. Recent work has shown that GO, regardless of lateral dimensions, interacts significantly with the plasma membrane of a panel of cell lines (BEAS-2B, NIH/3T3, HaCaT, 293T).^404^ However, the subsequent uptake mechanism is dependent on the lateral dimensions of the material. It was shown that small GO was internalized mainly via micropinocytosis, while ultrasmall GO was mainly internalized via clathrin- and caveolae-mediated endocytosis. Interestingly, a shift from macropinocytosis to clathrin-dependent endocytosis in the uptake of small GO was demonstrated after 24 h. Importantly, both small GO and ultrasmall GO ended up in lysosomal compartments after 48 h.^404^ Several other studies have also demonstrated that lateral dimensions of GBMs govern cell uptake. Professional phagocytic cells are capable of taking up GBMs with a range of lateral dimensions,^405^ whereas nonphagocytic cells either do not take up large materials (>1 mm) or internalize the materials by micropinocytosis.^404,406^ Notably, uptake of nanomaterials is also influenced by the presence (or absence) of a protein biocorona.

### The Biocorona of 2D Materials

The biocorona refers to the coating of biomolecules (proteins, lipids, sugars, nucleic acids, and metabolites) on the surface of nanomaterials, endowing the nanomaterials with a biological “identity”.^407^ The composition of the biocorona of 2D materials drives cellular interactions not least in the immune system.^210^ The protein corona is the most widely studied biocorona, but other biomolecules including lipids are also present in various biofluids and have been identified in the biocorona of GBMs.^408^ Early work showed that the biocorona mitigated the cytotoxicity of GO.^409^ Some investigators suggested that this is due to less uptake (endocytosis) of GBMs.^410^ However, the latter studies were performed using a lung adenocarcinoma (nonphagocytic) cell line. Professional phagocytic cells may respond differently as the presence of specific proteins or “opsonins” in the corona may direct the nanomaterials toward specific uptake pathways. Moreover, surface modification, e.g., PEGylation, also modifies cellular uptake of GBMs.^411^ Different surface chemistries (e.g., GO, rGO, and FLG) and lateral dimensions also result in the adsorption of different proteins on the GBM surface^412^ but also in different relative orientations of these biomolecules,^413^ exposing different epitopes for the interaction with cellular receptors. The percentages of immune-relevant corona proteins in graphene, borophene, and phosphorene were reported to be 41.3%, 46.5%, and 75.6%, respectively, indicating that graphene and borophene were not effective immune regulators. Several studies on protein corona on GBMs are available focusing on GO and how the protein layer mitigates its adverse effects *in vitro*.^414−416^ Theoretical studies suggested that the protein corona is able to reduce toxicity by reducing the physical interactions between the GO sheets and the cell membrane.^417^ The role of the corona was also investigated in relation to GO cytocompatibility and antimicrobial properties.^418,419^ Other studies addressed the role of the biocorona for the uptake of FLG and rGO. Hence, long-term colloidally stable dispersions of FLG (lateral dimensions of about 200–300 nm) were prepared in the biological exposure medium in which the materials were studied (human serum, or fetal bovine serum, FBS) at the concentrations of 10%, 50%, and 100% in PBS. Naturally, the resulting FLG presented a biocorona of serum proteins.^420^ The authors were able to map some functionally relevant epitopes that are known to mediate binding to specific receptors on cells in the liver. In a follow-up study, the authors focused on one such corona protein, apolipoprotein A-I, and observed that the uptake of FLG was “somewhat” increased in cells expressing the cognate receptor, scavenger receptor B1.^421^ Other investigators used sodium cholate to prepare stable aqueous dispersions of rGO. The authors hypothesized that changing the cholate concentration in the dispersion would alter the surface properties of rGO.^422^ To this end, rGO with varying concentrations of sodium cholate were prepared with or without a protein corona derived from a 1-h incubation in culture medium containing 10% FBS. The results revealed that the rGO dispersed in a lower surfactant concentration exhibited higher protein adsorption, and a stronger cytotoxicity. However, the surfactant itself also displayed cytotoxicity, and cell type-specific differences in susceptibility were noted. Thus, the interplay between rGO, the dispersant, and the biocorona, is complex. Finally, emerging data point toward the possibility of intracellular protein corona formation in macrophages.^235^

Other forms of biotransformation of GBMs apart from biocorona formation may also occur. Dissolution is an important parameter to take into account when addressing the biological or environmental effects of 2D materials “beyond graphene”.^93^ Biotransformation could be affected by the adsorption of biomolecules found in body fluids (for instance, in the airways, in the bloodstream, or in the gut), or be driven by enzymatic degradation in the body (for instance, following oral ingestion).^344^ In a recent study, an *in vitro* model of the intestinal epithelium was applied to simulate oral, gastric, and small intestinal digestion of GBMs.^345^ The authors concluded that occasional ingestion of small quantities of 2D materials such as hBN, MoS_2_, WS_2_, and GO is unlikely to be highly cytotoxic. The study reported significant agglomeration of all materials during digestion, especially GO, which was probably due to interactions with digestive proteins. Notably, the MoS_2_ sheets had dissolved by ∼75% after simulated digestion.^345^ Biologically relevant biotransformation of MoS_2_ nanodots was reported in an *in vivo* study in which the MoS_2_-HSA complexes were administered intravenously in mice at 5 mg/kg of body weight.^310^ The nanodots were found to accumulate largely in the liver and spleen, and this biodistribution was accounted for by the presence of apoE in the biocorona. Moreover, biotransformation in the liver, potentially through the actions of phase I enzymes such as cytochrome P450 enzymes, resulted in the incorporation of molybdenum into molybdenum-dependent enzymes.

The biological interactions of MoS_2_ are increasingly being studied.^423^ Early work demonstrated that exfoliated pristine and covalently functionalized MoS_2_ were internalized while exerting minimal pro-inflammatory cytokine release in macrophages.^151^ MoS_2_ is taken up by macrophages,^215^ and the pro-inflammatory effects have been attributed to the adsorption of fibrinogen and IgG.^246^ MoS_2_ nanosheets were found to interact with the plasma membrane of cells and are taken up via endocytosis in a size and cell type-dependent manner.^424,425^ Subcellular trafficking takes place through vesicular maturation from early, through late endosomes, toward the lysosomes, similarly to what has been reported for GO. Once reaching the lysosomes, the nanosheets either remain there or are slowly exocytosed from the cells.^425^ Larger MoS_2_ nanosheets (average size: 700 nm), are primarily internalized in cells through phagocytosis/micropinocytosis, while smaller MoS_2_ nanosheets (<300 nm), enter the cells either through the caveolar or clathrin-mediated uptake pathways, comparable to GO.^424,425^

### Biodegradation of 2D Materials

Previous work has shown that carbon-based nanomaterials including SWCNTs and MWCNTs as well as GO and FLG can undergo enzymatic biodegradation.^28,210^ In an early study on enzymatic degradation of carbon-based nanomaterials, the plant enzyme, horseradish peroxidase (HRP), and low amounts of hydrogen peroxide were applied.^426^ Soon, GO was also shown to undergo HRP-dependent degradation in “test-tube” experiments.^427^ Importantly, several mammalian peroxidases (myeloperoxidase, MPO; eosinophil peroxidase, EPO; lactoperoxidase, LPO) were subsequently shown to “digest” carbon nanotubes.^428−430^ Notably, LPO-driven degradation occurred even in the presence of a biocorona of pulmonary surfactant proteins and lipids.^430^ However, MWCNTs are not as effectively degraded as their single-walled counterparts.^431^ Early work showed that GO can also undergo biodegradation in the presence of purified human MPO^432^ and when incubated with activated human neutrophils.^230^ GO is also degraded to some extent by EPO,^433^ and GQDs have been shown to be degraded both by MPO and EPO.^434^ Studies performed in the Graphene Flagship also revealed that FLG can undergo neutrophil-driven degradation *ex vivo*, but the process was much slower than for GO (i.e., several days, as opposed to a couple of hours for GO).^435^

Overall, most degradation studies have used qualitative measurements to evaluate degradation, e.g., transmission electron microscopy, atomic force microscopy, and Raman spectroscopy, while few studies have explored quantitative approaches.^436^ However, it is noted that MPO is more effective than the HRP/H_2_O_2_ system.^437^

The degradation of GBMs has predominantly been studied in macrophages and neutrophils (and in the environment; see section Fungal and Bacterial Degradation). The mechanism of biodegradation in each cell type is distinct. Thus, while macrophage degradation of GBMs (such as graphdiyne oxide) was shown to occur intracellularly through a peroxynitrite-dependent pathway,^234^ neutrophils “digest” GBMs extracellularly, either through degranulation with the release of MPO, or through the formation of NETs that contain MPO as well as other granule proteins.^230,438^ Functionalization of GO with fMLP, a chemotactic peptide, was shown to trigger degranulation, leading to degradation in the absence of other stimuli.^27^ Evidence of degradation of GO was also observed in the lungs of mice following pulmonary exposure; hence, Raman imaging revealed the progressive biotransformation of GO into less graphitic structures.^266^ However, the mechanism was not disclosed. Moreover, in a thorough evaluation of the *in vivo* fate of GO following intravenous injection, Newman et al.^298^ could show that GO present in spleen-resident macrophages gradually underwent biodegradation over a period of 9 months postexposure. This work offers important information on biological processing and degradation of GO in mammalian tissues.

Biodegradation of MoS_2_ using HRP and MPO was found to be incomplete compared to that of GBMs.^151^ The degradation of MoS_2_ in human THP-1 cells was monitored 24 h after exposure,^439^ although the mechanism of degradation was not disclosed. The distribution and translocation of polyvinylpyrrolidone (PVP)-modified MoS_2_ nanosheets in cells, and their degradation in different biological environments (e.g., H_2_O_2_ alone, MPO plus H_2_O_2_, and catalase plus H_2_O_2_) have also been evaluated.^440^ It was found that MoS_2_ nanosheets were completely decomposed when incubated with MPO in the presence of H_2_O_2_. Furthermore, it was found that intravenously administered PVP-MoS_2_ was gradually cleared from mouse liver and spleen within 30 days. Early work on biodegradability of hBN evidenced the different actions of HRP and MPO. Partial oxidation was found using MPO, while HRP was unable to transform hBN even after 60 days.^441^ A degradation study of hBN in a lysosome mimicking solution showed boron release after 30 days.^442^

In synopsis, the fate of GBMs has been extensively investigated, and we now know that certain GBMs (especially GO) are biodegradable, and that the formation of a protein corona (or biocorona) may reduce toxicity, while other 2D materials have not been studied to the same extent.

## Life Cycle Perspective on 2D Materials

To fully understand the environmental impact of GBMs and other 2D materials, a life cycle assessment is needed that applies a “cradle-to-grave” perspective on the production, use, and disposal of these materials.^12,443^ Additionally, hazard assessment of 2D materials should take into account not only the as-produced or pristine material but should also adopt a life cycle perspective. For comparison, previous work addressed the toxicity of aerosols generated from sanding polymer-coated MWCNT-embedded composites to better mimic the hazard that may be encountered by workers or consumers.^444^ The authors found no evidence of free nanotubes in the aerosols and concluded that while the number of workers and consumers increases along the life cycle, toxicity and/or potential for exposure to the as-produced material may, in fact, be reduced. Here, we focus on the release of 2D nanofillers from polymer matrixes during the life cycle of the composite and explores acute impact on human health. For as-produced GBMs, there is a broad but as yet inconclusive understanding regarding their human health and environmental impact.^3^ In contrast, much less data is available on materials embedded in composites, and on 2D material release after abrasion, combustion, or weathering of products.

**Figure 21:**
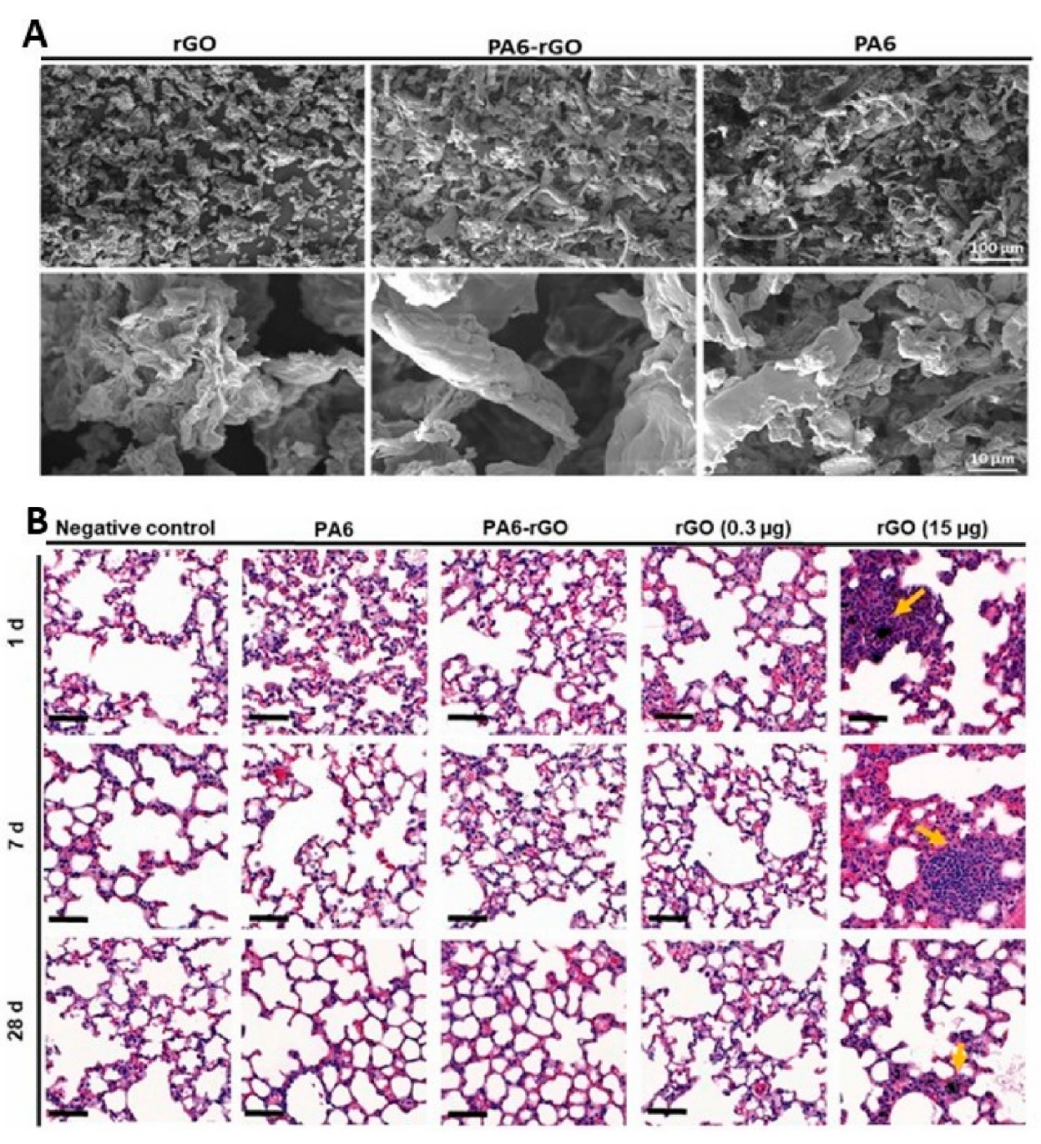
Hazard assessment of thermoplastic composites reinforced with reduced graphene oxide. (A) Characterization of rGO and abraded particles from PA6-rGO composites. SEM images of rGO, abraded particles from PA6-rGO composite and abraded particles from neat PA6. Animals (*n* = 3) were exposed by oropharyngeal aspiration to abraded polymer (PA6, 15 μg), abraded composite (PA6-rGO, 15 μg; with 2.5% rGO, hence 0.375 μg of rGO in 15 μg of PA6-rGO), reduced graphene oxide (rGO, 0.3 μg or 15 μg; 2.5% of 15 μg equals to about 0.3 μg), or negative control (BSA 0.1% in water). (B) Representative images of hematoxylin and eosin (H&E)-stained lung sections from mice exposed to rGO and abraded composites, following 1, 7, and 28 days after oropharyngeal aspiration. Arrows indicate the formation of granulomas after treatment with rGO.^280^ Reproduced with permission from Chortarea, S.; Kuru, O. C.; Netkueakul, W.; Pelin, M.; Keshavan, S.; Song, Z.; Ma, B.; Gomes, J.; Abalos, E. V.; Luna, L. A. V.; Loret, T.; Fordham, A.; Drummond, M.; Kontis, N.; Anagnostopoulos, G.; Paterakis, G.; Cataldi, P.; Tubaro, A.; Galiotis, C.; Kinloch, I.; et al. Hazard Assessment of Abraded Thermoplastic Composites Reinforced with Reduced Graphene Oxide. *J. Hazard. Mater.* 2022, *435*, 129053. Copyright 2022, Elsevier.

Synthetic polymers and plastics are a relatively modern category of functional materials. Possessing attractive properties such as low density or easy processability in contrast to their metallic counterparts,^445^ polystyrene (PS), polyvinyl chloride (PVC), and polyester resins have been produced in large quantities since the 1930s.^446^ The reinforcement of plastics with carbon or glass fibers^447^ has led to a huge diversity of composite materials using different polymers and fillers. The use of carbon-based nanomaterials as fillers is not limited to mechanical improvements, but also to induce electrical conductivity, antifouling properties, or to reduce flammability. Carbon-based as well as 2D nanofillers have become very popular since their addition at low amounts (1–5 wt %) have shown to drastically improve composite properties. In parallel with the broad use of nanofiller-reinforced polymers, the potential exposure to humans and environment is increasing and therefore careful risk and hazard assessment is needed to avoid social and economic drawbacks.^126,448^ Matrix degradation occurs mainly by mechanical forces, aggressive chemicals, or weathering (e.g., UV light and temperature) and can result in increased release of particles into the environment and therefore human exposure. Normal use involves forces much less than that during intentional drilling or sanding, which makes aerosol production less of an issue compared to occupational use. It is also important when analyzing toxicity to consider that nanoparticles on surfaces, as opposed to compounded in a matrix, are not directly comparable.^449^ Recent studies have shown that graphene-based polyester resin composites are released during machining and in weathered samples.^450^ Cell viability levels of around 60% in A549 human lung cancer cells were observed with high dose (100 ppm) graphene nanosheets and graphene composites. Both graphene and resin demonstrated low toxicity although the authors acknowledged the presence of ambient particles from the spraying process that comprised of various metals. In another study conducted in the Graphene Flagship, abrasion of 2.5 wt % rGO reinforced polyamide (PA6), a representative thermoplastic, yielded a greater amount and smaller size of released particles (average size 1.91 μm) for the PA6-rGO composite (average particle size 3.16 μm) (Figure 21).^280^

This is in contrast to sanded GBM-reinforced polyurethane, where fewer fragments were released than that of matrix alone and showed no changes in particle size and no release of free GBM compared to the neat polymer.^451−453^ The abraded FLG-reinforced epoxy polymer showed no significant shift in the particle size distribution or free graphene compared to pure epoxy materials.^296,454,455^ When combusted, GBM-reinforced poly(lactic) acid films showed increased flame-retardant properties, and unburned GBMs could be found in the ashes, in higher quantities compared to neat polymers.^456^ Combustion of FLG-reinforced epoxy increased thermal stability of the composite, with no changes in amounts of particles released.^457,458^ When exposed to weathering, the composite reacted in the same way as the matrix, with lower amounts of particles released when exposed to UV light alone, but the same amounts upon simulated rain. However, free graphene flakes were observed.^452,453^ Based on the relatively few studies we can nevertheless extrapolate that depending on the combination of the matrix and the amount of nanofiller, only a moderate to low release of GBMs can be expected. However, despite the small amounts of GBMs released after the different treatments, careful assessment of abraded material is still warranted. Few studies have addressed the released materials with respect to toxicity. FLG-reinforced epoxy after abrasion or after combustion displayed no or only very moderate and transient cytotoxicity.^454,457^ A similar result was observed in the multilaboratory analysis of rGO-PA6 abraded particles.^280^ Only as-produced rGO at the high dose of 40 μg/mL triggered adverse effects, most notably in macrophages. Since inhalation of airborne materials is mainly occupational, the effects after 1, 7, and 28 days after single pulmonary exposure were evaluated in mice. In agreement with *in vitro* data, rGO-PA6 abraded particles induced only modest and transient pulmonary inflammation.^280^

To sum up, relatively low amounts of 1–5 wt % 2D nanofillers in composites significantly improved polymer properties. During use and end-of-life, release of 2D nanofillers after abrasion, weathering or combustion is negligible. Particle size distribution after various stresses did not change compared to neat polymers; however, depending on combination of polymer and nanofiller, there remains a possibility of free or protruding GBMs. On the basis of current knowledge, the use of 2D nanofillers in composites could be considered a benefit that outweighs the risk. At present, this type of analysis is lacking for other 2D materials beyond GBMs.

## Occupational Exposure to 2D Materials

Human inhalation exposure to 2D materials is most likely to occur in the occupational environment, and is associated with various activities in material processing and handling. Worker exposure to GBMs and other 2D materials is related to the safety and emission control of processes and activities in the synthesis and manufacturing. Occupational exposure can be potentially significant in end-of-life scenarios, such as recycling and waste handling. Following our previous review,^3^ few papers have been published on occupational exposure studies on these materials. The focus has been on production,^459,460^ or research and development^461,462^ in the workplace covering early life-cycle stages of the material, therefore leaving a dearth of knowledge on the later stages of occupational exposure including waste handling and recycling.

The Organisation for Economic Co-operation and Development (OECD) (ENV/JM/MONO(2015)19)^463^ and European Committee for Standardisation (CEN) approach (EN-16966:2018)^464^ for NOAA (Nano-Objects and their Aggregates and Agglomerates) assessment provides a clear and reliable view on the exposure situation in different and often dynamic industrial and R&D/Pilot occupational exposure scenarios. The widely harmonized multimetric tiered approach for workplace exposure measurement strategy and methods thus provides guidance for three tiers of assessment. Tier 1 is initial assessment, where the potential for release and emission of nano-objects (including 2D materials) into the workplace air is determined. Relevant workplace, process and production activity information is gathered structurally, according to best practices in occupational hygiene. Together with detailed material information, the possibility of release of nanomaterial can be considered. If Tier 1 shows potential for nanomaterial exposure, evaluation should proceed to Tier 2. Moreover, control or risk banding tools can be used to examine exposure potential at work. Most of these tools already have a Tier 1-type approach with structured questions focused on determination of potential release or exposure. Some examples can be found in the ISO technical specification on nanotechnologies (ISO/TS 12901-2:2014). Tier 2 is aimed at obtaining an indication of exposure to nano-objects. In this basic assessment, exposure is investigated using easy-to-use and portable measurement equipment to detect airborne nanomaterial (nanoparticles, aerosol) levels in real-time during process and activities. The off-line sampling and analysis of workplace air to characterize possible nanomaterials is combined with real-time assessment with techniques such as electron microscopy. It is noted, however, that there are currently no consensus methods for off-line analyses of most nanomaterials.^465^ Tier 3 is the expert assessment of personal exposure to airborne particles. The aim is to comprehensively characterize exposure to airborne particles in the breathing zone of the workers. This requires state-of-the-art techniques and methods, and evaluations should include considerations and comparisons to the corresponding reference values currently available. The appropriate measurement techniques should cover the largest size-range of particles currently available (10 nm to 10 μm), have a suitable time resolution to monitor sudden changes in concentrations due to work activities, and include size-integrated metrics, such as particle surface area, number concentration and mass concentration. Typically, as airborne particles, 2D materials can be both nano- and micron-size NOAA, with high variability in, for example, morphology and state of agglomeration. Thus, it remains challenging to selectively identify and quantify these particles for exposure assessment purposes when current state-of-the-art online instruments are designed for theoretical spherical object measurements. At present, discussions and considerations of the most useful combination of measurement methods and metrics combining online and off-line assessments to assess potential health effects are still ongoing.^466^ Exposure situations and exposure potential in the workplace can be grouped according to the state of the material during a specific work process. Harmful emissions are most probable in the dry state, and less probable in the liquid or paste state, when aerosol formation is not likely (CEN EN-17058).^467^ Currently, most production and related handling phases are in the liquid/paste states of the material, and thus related emissions and exposure potentials remain low.^182,459,460^ The final stages of synthesis/production process, when the raw material, such as produced GBMs or other 2D materials, is dried and packed for further use, are the most critical points regarding worker exposure, in addition to equipment maintenance and cleaning, where dry material can be released accidentally. The exposure (Tier 1 and Tier 2) levels in CVD graphene production were previously analyzed in an R&D laboratory.^461^ The findings could not prove any graphene particle emission during any process. However, the need for more detailed Tier 3 assessment was emphasized to achieve a more comprehensive assessment. A GO pilot production process was then studied by applying Tier 2 methodology.^468^ The implemented safety measures proved efficient, and no exposure was detected in the process. More recently, GNP and GO emissions and exposures were investigated during downstream industrial handling, showing that powder handling contributes to the highest particle emission and exposures.^466^ Overall levels remained low but the importance of a multimetric approach to study worker exposure was again highlighted. In the Tier 3 study of producing and processing FLG in a pilot production laboratory,^459^ potential release of FLG could not be excluded, especially in the final process stages or equipment cleaning. Exposure was not clear based on particle measurements, but the filter sample collected from worker breathing zone unexpectedly detected FLG. The low exposure levels detected in recent studies can be related to utilization of proper and efficient technical control measures, and using closed or segregated systems to prevent release of harmful emissions in workplace air. Nevertheless, challenges remain with respect to risk assessment and risk management of 2D materials. The multimetric approach can give relevant and essential information on exposure scenarios, but the lack of associated occupational exposure limit (OEL) values and the ever-expanding knowledge gap on human health effects of 2D materials have led to the use of precautionary principles in exposure control and risk management. One suggested approach is the utilization of safe-by-design principles to cover the whole life cycle of the product, from innovation, development, and production to end-of-life.^469^

## Regulatory Perspective on 2D Materials

The bulk of all studies related to the toxicity or ecotoxicity of 2D materials are performed using nonstandardized protocols, and the aim is not always to support regulation; instead, the focus is on achieving a mechanistic understanding of the (potential) toxicity of 2D materials. However, standardized test protocols are required to support the regulation of up-to-date materials. The OECD has addressed Test Guidelines (TGs) and Guidance Documents (GDs) to evaluate the safety of chemicals for over 60 years, and because nanomaterials are chemicals, they are also included. The OECD TGs build on the need of Mutual Acceptance of Data (MAD), hence facilitating regulatory acceptance of data and avoiding experimental duplication, especially of *in vivo* (animal) testing. In Europe, nanomaterials fall under the Registration, Evaluation, Authorisation and Restriction of chemicals (REACH) regulation, (EC 1907/2006).^470^ However, particular issues regarding nanosized materials have been identified, which challenge some of the TGs and GDs developed for conventional chemicals. To this aim, the Working Party on Manufactured Nanomaterials (WPMN)^471^ was established in 2006 and the Sponsorship Programme for the Testing of Manufactured Nanomaterials (Testing Guideline Programme, TGP)^472^ was launched in 2007. The Testing Programme verifies testing methods applied on nanomaterials by pooling the expertise of OECD member countries, some nonmember countries, and other stakeholders to fund safety testing of specific nanomaterials. Initially the Testing Programme focused on 11 materials of industrial relevance, namely cerium oxide, fullerenes, dendrimers, gold nanoparticles, MWCNTs, nanoclays, silicon dioxide, silver nanoparticles, SWCNTs, titanium dioxide, and zinc oxide. Following on from these initial activities, efforts focused on identification of needs regarding adaptations of guidelines to nanomaterials (ENV/JM/MONO(2009)21).^473^ These efforts fall under the OECD WPMN in collaboration with the Working Group of National Co-ordinators of the TGP (WNT), and the status of the work is reviewed yearly through a work plan publication.

The large European research project NanoReg (85 partner institutes) funded under the FP7 program had as an overarching aim the development of a common European approach to the regulatory testing of nanomaterials in terms of environmental, health and safety issues. One of the key results was to evaluate the applicability of several TGs to nanomaterials and to highlight shortcomings where these were present. Several recommendations were made, which have been summarized in the so-called ProSafe “white paper”.^474^ To meet the regulatory needs, an action plan was set up in 2017 to support the amendment and development of TGs for nanomaterials and especially nanoforms in REACH. This is known as the “Malta Initiative” (so named as the initiative arose during the Maltese EU Council presidency) and brings together EU member states, the European Commission (including Directorate-General Research and Innovation, Directorate-General Environment, and Joint Research Centre, JRC), the European Chemicals Agency (ECHA), industries, and other institutions. The Malta Initiative is a voluntary instrument without an official/legal mandate. The OECD MAD ensures that test results generated in accordance with OECD TGs and the OECD Principles of Good Laboratory Practice (GLP) are accepted in all OECD countries and adherent countries. Therefore, OECD TGs are key for internationally harmonized and standardized safety testing of chemicals and materials for governments, industry, and academia. To date, the Malta Initiative has facilitated updating and modification of 18 TGs, ensuring that they are fit for purpose for nanomaterials. The output of the Malta Initiative shows that a coordinated effort leads to successful TG development. Such effort includes: (a) funding of researchers for the validation and harmonization of test methods, and (b) an international platform for collaboration and exchange between stakeholders. The general aims are to identify relevant end points and methods ready for validation and harmonization; to support collaboration of researchers, regulators, and industry in TG development; to ensure the development of test methods that are operable and useful in (pre) regulatory and scientific testing; to increase the chances for (effective) adoption by OECD member countries. Despite the important progress made, gaps in method developments for nanomaterials remain, and up-to-date developments in material innovations require further method developments. In collaboration with the OECD, the European Commission has funded further activities under the Malta Initiative to foster development of up-to-date or adapted TGs or GDs for the safety assessment of nanomaterials. Thus, Horizon 2020 projects such as NanoHarmony and Gov4Nano, in collaboration with the NanoMet project at OECD, are focused on the development of TGs and GDs to cover regulatory gaps identified by the Nanomaterial Expert Group (NMEG) at ECHA.

Despite the large efforts put forward by different initiatives at the EU level, uncertainties regarding safety assessment of nanomaterials still exist. One example is the recent revoking of the Commission Delegated Regulation of 2019 regarding harmonization and labeling of TiO_2_ as a carcinogenic substance by inhalation in certain powder forms by the European Court of Justice (EU Press Release No. 190/22).^475^ Thus, this points to a lack of reproducibility of results as a consequence of a lack of harmonized protocols and the long time required to achieve approval of protocols at the OECD level. To accelerate this process, several experts have already published adapted protocols to address particular issues related to nanomaterials, even if these have not yet obtained OECD approval. Examples include protocols produced in European Commission funded projects (NanoReg, NanoTest, PATROLS, GRACIOUS, RiskGONE, etc.) such as the adaptation of cytotoxicity tests to avoid nanomaterial interference,^476^ adaptations of genotoxicity assays so that they are more reliable for nanomaterials,^477^ and the implementation of F.A.I.R. (findability, accessibility, interoperability, and reusability) data principles in nanosafety research.^478^ However, gaps still exist, and a prioritization scheme is required like the one put forward by the NMEG that led to the work performed in Gov4Nano and NanoHarmony. In a recent review coordinated by the Dutch National Institute for Public Health and the Environment (RIVM), a comprehensive survey was provided of the information requirements in all areas of European legislation that are applicable to nanomaterials and needs for further action to address nanospecific issues were identified.^479^ Overall, harmonization efforts concluding at OECD level will help improving transferability and reproducibility of results from different laboratories and, hence, will contribute to the current uncertainty gathered around nanosafety data from the literature, supporting regulatory decision making and making a positive impact in technology development. This is certainly true not only for “traditional” nanomaterials but for 2D materials as well. The Graphene Flagship has addressed the need for the harmonization of OECD TGs and GDs with respect to the ecotoxicity and human toxicity testing of GBMs. Regarding ecotoxicity, data generated in the framework of the Graphene Flagship may be useful in the revision of the GD on Aquatic Toxicity Testing of Nanomaterials, generating annexes to explain the applicability of TG 201 (Algae and Cyanobacteria Growth Inhibition Test), TG 202 (Daphnia Acute Immobilization Test), and TG 203 (Fish Acute Toxicity Test) to nanomaterials and GBMs. Additional work has been done in relation to a more recent GD on assessing the apparent accumulation potential for nanomaterials, and although all the protocols and steps for the application of TG 305 (Bioaccumulation in Fish) to GBMs are evident, the lack of routine methodologies for the determination of GBM concentrations in biological tissues constitutes an essential limitation.

Regarding OECD TGs related to human toxicity, the work performed in the Graphene Flagship has focused mainly on skin safety. In general, OECD TGs predicting skin toxicity assess skin irritation, corrosion, and sensitization. These *in vitro* TGs employing a 3D model of artificial epidermis (OECD TG 439 and 431) can be adopted for GBMs without modification,^188,190^ with OECD TG 442B also demonstrated to be applicable to GBMs.^187^ However, technical limitations with regard to the TGs evaluating *in vitro* the initial three phases of skin sensitization AOPs, namely reactivity with peptides (OECD TG 442C), keratinocytes activation (OECD TG 442D), and dendritic cells activation (OECD TG 442E), were identified and relevant modifications of the procedures are needed before TG adoption for GBMs. Initial information has been included in an OECD report on the applicability of OECD TG 442D in nanomaterial testing.

Members of the Graphene Flagship have thus assessed skin irritation using the SkinEthic^TM^ reconstructed human epidermis, following OECD TG 439. Even though not validated for nanomaterials, the OECD TG 439 turned out to be applicable also for GBM testing, since no interference with the methylthiazolyldiphenyl-tetrazolium bromide reduction, used as a readout, was found.^188^ On the same model, skin corrosion of GBMs was very recently evaluated following the OECD TG 431.^190^ Furthermore, skin sensitization by FLG and GO was evaluated following the OECD TG 442B (Local Lymph Node Assay).^187^ This *in vivo* study following OECD TG 442B demonstrated the absence of skin sensitization properties for two representative GBMs, FLG and GO.

## Conclusions and Future Perspectives

The Graphene Flagship (2013–2023), a combined academic-industrial consortium funded by the European Commission, has succeeded in building a foundation for a graphene industry in Europe.^480,481^ Human health and environmental issues have always been a part of this endeavor. Hence, we prepared and published a “midterm report” in 2018 with a survey of the literature on safety assessment of graphene and related materials.^3^ Here, we have expanded this discussion to other 2D materials. Together, the two reports provide an overview of the state-of-the-art of the safety assessment of graphene and other 2D materials with respect to human health and the environment. We remarked in the previous review that all GBMs cannot be grouped together as one material. Indeed, GBMs may vary considerably in terms of intrinsic physicochemical properties,^6^ leading to differences in terms of their interactions with biological systems. This is also true for the ever-expanding universe of other 2D materials including MXenes, TMDs, etc.^482^ Since limited (eco) toxicological data are available on more recent advanced 2D materials like MXenes, 2D metal organic frameworks, perovskites, layered double hydroxides, and many others, efforts to assess their potential impact on health and the environment are warranted. Furthermore, as we have discussed in the present review, GBMs and other 2D materials may undergo biotransformation. It follows that it is not sufficient to characterize the properties of as-produced 2D materials; toxicologists must also consider the many transformations (e.g., agglomeration, dissolution/degradation, coronation) that may occur in the natural environment or in the human body, both in the extracellular and in the intracellular compartment. Thus, 2D materials may be regarded as dynamic entities, displaying an evolving synthetic identity as well as a biological identity that is determined, at least in part, by the adsorption of biomolecules. This is certainly exciting and worthy of exploration from an academic point of view, but the realization that 2D materials are not one single material, and the understanding that these materials interact in a dynamic fashion with biological systems, also has considerable implications for the regulation of 2D materials. Regulation could have positive and negative effects on the innovation process, but we ignore the safety assessment of 2D materials and other advanced materials at our peril.

Significant advances have been made in the development of 2D material-based sensors to detect biomarkers for various diseases. In certain cases, tissues from different organs might come into direct contact with the materials embedded into these devices. Although the surface contact of the organs to the 2D materials is likely very limited, we suggest the scientific community to carry out toxicity studies about the potential undesired effects on tissues beyond biomarker sensing.^483,484^

In the present review, we have also discussed “green” chemistry approaches to minimize the environmental footprint of 2D materials. This aligns well with the “safe-and-sustainable-by-design” (SSbD) concept that is embedded in the European Commission’s Chemicals Strategy for Sustainability (CSS). The so-called SSbD approach (OECD ENV/CBC/MONO(2022)30)^485^ requires “life cycle thinking” to ensure sustainability along the entire value chain. Toxicological assessment is, of course, an important element, but chemicals and, by extension, nanomaterials, should be safe and sustainable both at the manufacturing phase, at the use phase, and at end-of-life of the product.

We have also attempted a careful description not only of the test material, but also the test system, and to the extent that this is possible, the test method/assay/end point. The reason for this is very simple: to enable a comparison between different studies. Indeed, the research community has recently addressed the need for a “Minimum Information Reporting in Bio-Nano Experimental Literature” (MIRIBEL) for published accounts of so-called bionano research.^486,487^ This encourages researchers in the field of toxicology to adhere to certain reporting standards in order to enhance research quality and avoid unnecessary duplication of experimental work.

It is worth noting that the interactions between 2D materials and biological systems are reciprocal. Hence, 2D materials may have an impact on cells and tissues, causing toxicity, but biological systems can to a certain extent detoxify 2D materials through enzymatic degradation. Importantly, this has been demonstrated both in mammalian systems, and in the environment. Understanding and controlling these processes may suggest novel strategies by which to mitigate the toxicity of 2D materials. This is certainly relevant not only for inadvertent exposure to 2D materials, but may also have ramifications for the clinical translation of (selected) 2D materials.

Looking ahead, we expect to see increasing numbers of 2D materials and 2D material-based applications in the years to come, and safety assessment will thus remain a strong priority, and should be viewed as an integral component of every 2D material research or innovation project. To this end, we need to develop and deploy robust test protocols based on relevant model systems to ensure that emerging 2D materials are safe for human health and the environment.

## Vocabulary

**Two-dimensional materials**: a family of ultrathin crystalline materials wherein atoms are organized in single- or few-layers, for instance graphene (a crystalline allotrope of carbon)
**Green chemistry**: chemistry focusing on environmental impacts with the design of chemical products and use of processes that reduce the use or generation of hazardous substances
**Biocorona formation**: the coating of biomolecules (proteins, lipids, sugars, nucleic acids, etc.) onto the surface of materials, endowing the materials with a different biological “identity”
**Biodegradability**: the ability of a material to decompose into smaller fragments and/or ions after interactions with oxidative enzymes present in the human body or in the environment
**Biodistribution**: tracking where compounds of interest travel in an experimental animal or human subject the biodistribution of materials is controlled by many factors including size
**Life cycle assessment**: methodology for the assessment of environmental impacts associated with all stages of the life cycle of a product, process, or service (i.e., from “cradle” to “grave”)
**Safe and sustainable-by-design**: an approach to support design, development, production and use of chemicals and materials while minimizing impacts on health and the environment
